# What Does It Take to Synergistically Combine Sub-Potent Natural Products into Drug-Level Potent Combinations?

**DOI:** 10.1371/journal.pone.0049969

**Published:** 2012-11-28

**Authors:** Chu Qin, Kai Leng Tan, Cun Long Zhang, Chun Yan Tan, Yu Zong Chen, Yu Yang Jiang

**Affiliations:** 1 Department of Pharmacology and Pharmaceutical Sciences, School of Medicine, Tsinghua University, Beijing, P. R. China; 2 The Ministry-Province Jointly Constructed Base for State Key Lab-Shenzhen Key Laboratory of Chemical Biology, the Graduate School at Shenzhen, Tsinghua University, Shenzhen, P. R. China; 3 Bioinformatics and Drug Design Group, Department of Pharmacy, and Center for Computational Science and Engineering, National University of Singapore, Singapore,Singapore; 4 NUS Graduate School for Integrative Sciences and Engineering, Singapore, Singapore; Concordia University Wisconsin, United States of America

## Abstract

There have been renewed interests in natural products as drug discovery sources. In particular, natural product combinations have been extensively studied, clinically tested, and widely used in traditional, folk and alternative medicines. But opinions about their therapeutic efficacies vary from placebo to synergistic effects. The important questions are whether synergistic effects can sufficiently elevate therapeutic potencies to drug levels, and by what mechanisms and at what odds such combinations can be assembled. We studied these questions by analyzing literature-reported cell-based potencies of 190 approved anticancer and antimicrobial drugs, 1378 anticancer and antimicrobial natural products, 99 natural product extracts, 124 synergistic natural product combinations, and 122 molecular interaction profiles of the 19 natural product combinations with collective potency enhanced to drug level or by >10-fold. Most of the evaluated natural products and combinations are sub-potent to drugs. Sub-potent natural products can be assembled into combinations of drug level potency at low probabilities by distinguished multi-target modes modulating primary targets, their regulators and effectors, and intracellular bioavailability of the active natural products.

## Introduction

Natural products (NP) have been traditional sources of drug discovery and there are renewed interests in them for new drug discovery [1], [2], [3], [4]. In particular, NP combinations have been extensively studied [5], [6], tested in clinical trials [7], [8], [9], and widely used in traditional, folk and alternative medicines [10], [11]. Their novel multi-targeted mechanisms [8], [12], [13] or molecular scaffolds [14] may be valuable sources for developing multi-targeted therapeutics [15]. Opinions vary regarding to the therapeutic efficacies of NP combinations. One attributes the efficacies of NP combinations to placebo effects [16], [17], [18] based on indications from clinical trials [17], [18] and the findings that bioactive NPs are typically sub-potent to drugs [19], [20]. Another credits the efficacies of NP combinations to synergistic effects [6], [8], [19], [21], [22] based on the findings that some NP combinations produce significantly better effects than equivalent doses of their components [19], [22] and clinical outcomes are not necessarily influenced by positive beliefs [16].

The contribution of synergistic effects to therapeutic efficacies has been extensively studied [6], [8], [22]. While many studies have consistently suggested that therapeutic potency can be enhanced by synergistic effects, the levels of potency enhancement, particularly with respect to those of drugs, have not been sufficiently studied to quantitatively assess the contribution of synergism to the therapeutic efficacies of NP combinations. In particular, four important questions need to be answered: what are the gaps between the potencies of the typically studied bioactive NPs and those of drugs, whether synergistic combination of sub-potent NPs can sufficiently enhance their collective potencies to reach drug potency level, and at what odds and by what molecular modes such NP combinations can be assembled.

The first question was studied by analyzing the literature-reported cell-based potencies of 190 approved drugs and 1378 NPs of anticancer and antimicrobial classes. Potencies derived from cell-based assays were used instead of target-based and *in-vivo* assays for several reasons. To a certain extent, cell-based assays can predict *in-vivo* activities [23], [24] and these assays have been successfully used for discovering therapeutic agents that have entered advanced development stages [25]. Within the same disease classes, cell-based assays are more mutually comparable and better reflecting overall effects than target-based assays. The number of NPs with cell-based potency data is significantly higher than those with *in-vivo* data. The anticancer and antimicrobial classes were particularly focused because of the availability of statistically significant number of cell-based activity data, the relatively comparable bioassays than some other therapeutic classes, and the relevance to our NP combination studies (67% of our studied synergistic NP combinations are from these two classes).

The second question was addressed by evaluating 124 literature-reported synergistic combinations of 158 NPs with cell-based activity data available for all of the constituents both in individual and in the respective combination. These data are necessary for deriving combination index (CI) and dose reduction index (DRI) for rigorous evaluation of synergistic effects [26]. The third question was probed by analyzing 122 molecular interaction profiles (MIPs) in 19 NP combinations with potencies enhanced to drug level or by over 10-fold. These MIPs are linked to the potency-enhancing synergistic molecular modes involving collective modulation of the primary targets, their regulators and effectors, and the pharmacokinetics of the active NP ingredients [8], [12].

While these 122 MIPs have been individually reported in the literatures, few of them have been collectively analyzed for probing potency enhancing molecular modes in NP combinations. It is cautioned that, although connections can be made between these MIPs and the synergistic potency-enhancing modes, many of these interconnections are much more complicated than those analyzed here. Their activities are highly dynamic [27], [28], [29] influenced by genetic variations [30], environmental factors [31], host’s behavior [32], and therapeutic scheduling [33]. Their use should be more appropriately viewed as a start to a more comprehensive analysis of the potency-enhancing modes in NP combinations.

## Materials and Methods

Experimentally determined cell-based inhibitory activities of anticancer and antibacterial drugs and NPs were searched from the Pubmed database [34] by using keyword ‘drug’, ‘natural product’, ‘herb’, ‘medicinal plant’, ‘extract’, ‘ingredient’, ‘GI50’, ‘IC50’, ‘MIC’, “activity”, ‘cell-line’, and ‘in vitro’. Cell-based inhibitory activities of 88 anticancer and 102 antimicrobial drugs were obtained from the literatures and the NCI standard agent database (**Table S1** and **S2**). Their approval status was further checked against the drug data in the Therapeutic target database [35]. Cell-based inhibitory activities of 1378 anticancer and antimicrobial NPs (**Table S3** and **S4**) and 99 antimicrobial NP extracts (**Table S5**) were obtained from the literatures. These activities are typically given as GI50 or IC50 values against cancer cell-lines or MIC values against microbial cells. For drugs and NPs with multiple potency data, the best potency was selected.

Literature-reported synergistic NP combinations were searched from the Pubmed database [34] by keywords ‘natural product’, ‘herb’, ‘medicinal plant’, ‘extract’, ‘ingredient’, ‘synergistic’, ‘synergy’, ‘synergism’, ‘synergize’, and ‘potentiate’. Although many NP combinations are synergistic [6], [8], [22], only 124 synergistic combinations of 158 NPs are with sufficient cell-based data for computing CI and DRI values (**Table S6**). The cell-based activities of the constituent NPs in some of these combinations are given in terms of the percent inhibitory rates at particular concentrations. Their CI and DRI values were computed by using the median effect equation, the multiple drug effect equation, and the combination index theorem [26].

## Results and Discussion

### Comparison of the Potencies of Natural Products and Drugs in Cell-based Assays

Drug potency is context dependent, varying with assay, target and technology. Previous analysis has suggested that drugs in cell-based assays typically exhibit potencies of ≤1 µM [36]. Hence, we tentatively define drug potency level for anticancer and antimicrobial classes as GI50/IC50≤1 µM and MIC≤1 µg/mL respectively, which are satisfied by 76% anticancer and 86% antimicrobial drugs. In some cases, drug efficacy is not only determined by cell-based activities. A minority of drugs sub-potent in cell-based assays are nonetheless clinically efficacious by such additional mechanisms as immuo- and hormone modulations [37], [38]. While drug potency level can be more rigorously defined by considering these mechanisms, few drugs and NPs have been sufficiently studied for enabling such a consideration. It is more practically feasible to tentatively focus on cell-based activities that nonetheless reflect the potencies of most drugs and NPs.

**Figure 1:**
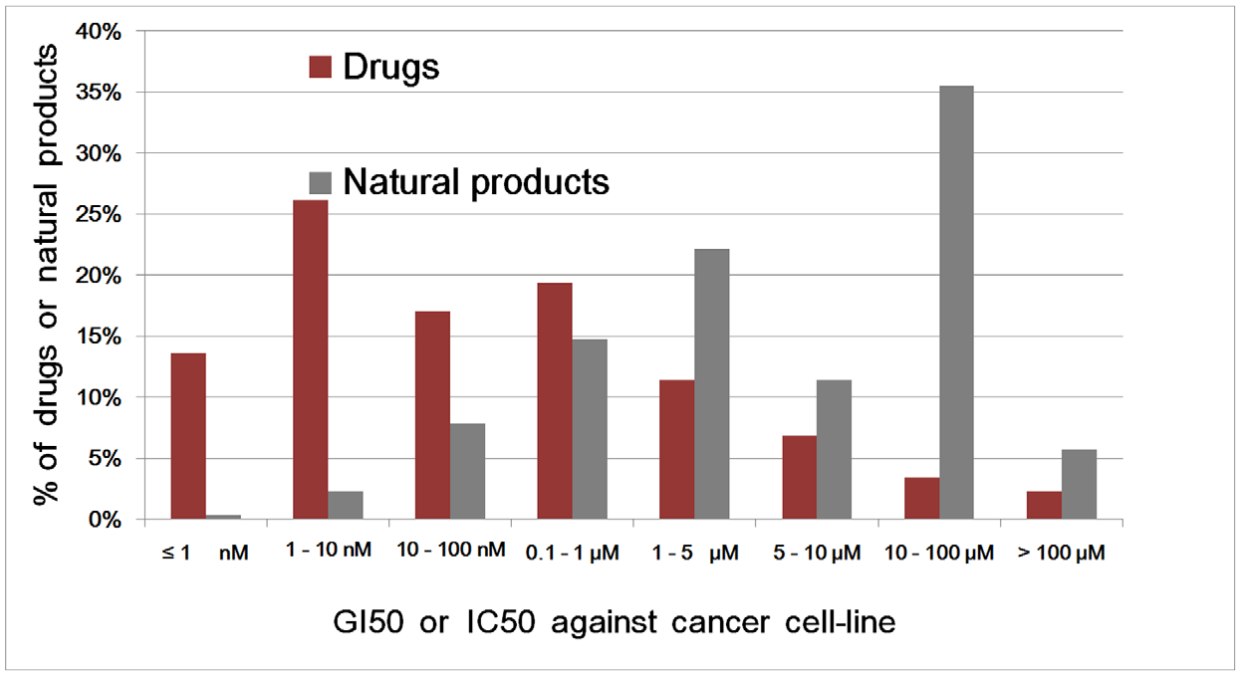
Potency distribution profiles of 88 and 650 anticancer drugs and natural products.

**Figure 2:**
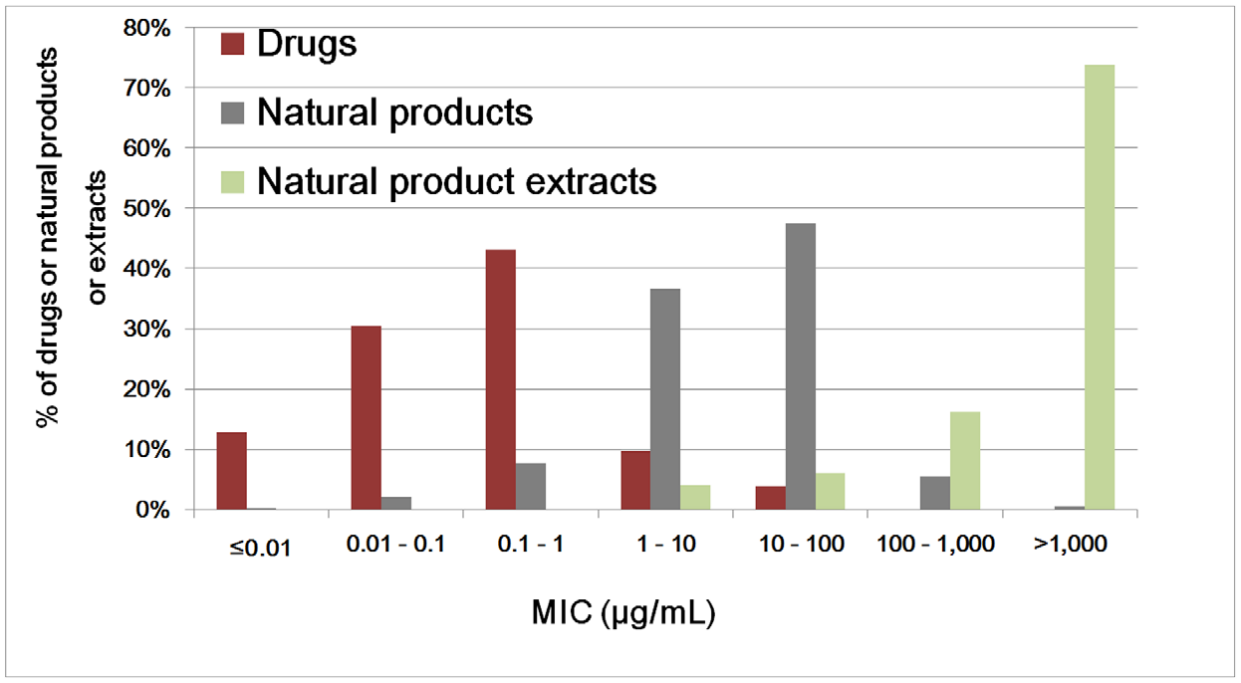
Potency distribution profiles of 102, 609 and 99 antimicrobial drugs, natural products (NPs) and NP extracts.

**Figure 1** and **2** show the potency distribution profiles of 88 and 650 anticancer drugs and NPs, and those of 102, 609 and 99 antimicrobial drugs, NPs and NP extracts respectively. The median potencies of anticancer (GI50/IC50 = 28 nM) and antimicrobial (MIC = 0.12 µg/mL) drugs are 214-fold and 104-fold higher than those of anticancer (GI50/IC50 = 6 µM) and antimicrobial (MIC = 12.5 µg/mL) NPs. Overall, 25% of the anticancer and 10% of the antimicrobial NPs reach drug potency level, and additional 33% of the anticancer and 37% of the antibacterial NPs are within 10-fold range of drug potency level (1 µM<GI50/IC50≤10 µM, 1 µg/mL<MIC≤10 µg/mL). The pool of potent NPs is relatively small (10–25%). A significantly expanded pool of active NPs (47–58%) may be explored if NP combinations of >10-fold potency enhancement can be assembled at reasonable probabilities. The potencies of the NP extracts are mostly 100–1,000 folds lower than those of individual NPs, as the active constituents only constitute a small portion of their contents [39]. Partly because of this gap, NP extracts have been typically prescribed in g/kg [40], [41] in contrast to the mg/kg ranges for drugs and NPs.

### Synergistic Natural Product Combinations

**Figure 3:**
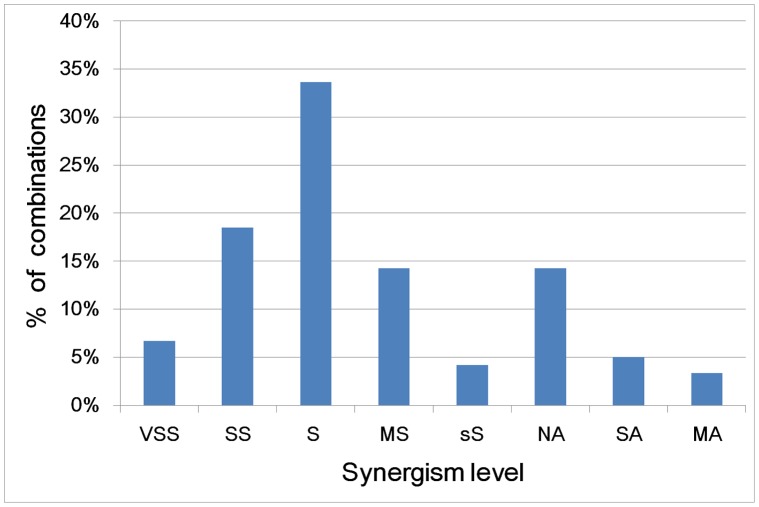
Synergism level of 124 synergistic NP combinations. VSS, SS, S, MS, sS: very strong, strong, normal, moderate, slight synergism, NA: nearly additive, SA, MA: slight, moderate antagonism.

**Figure 4:**
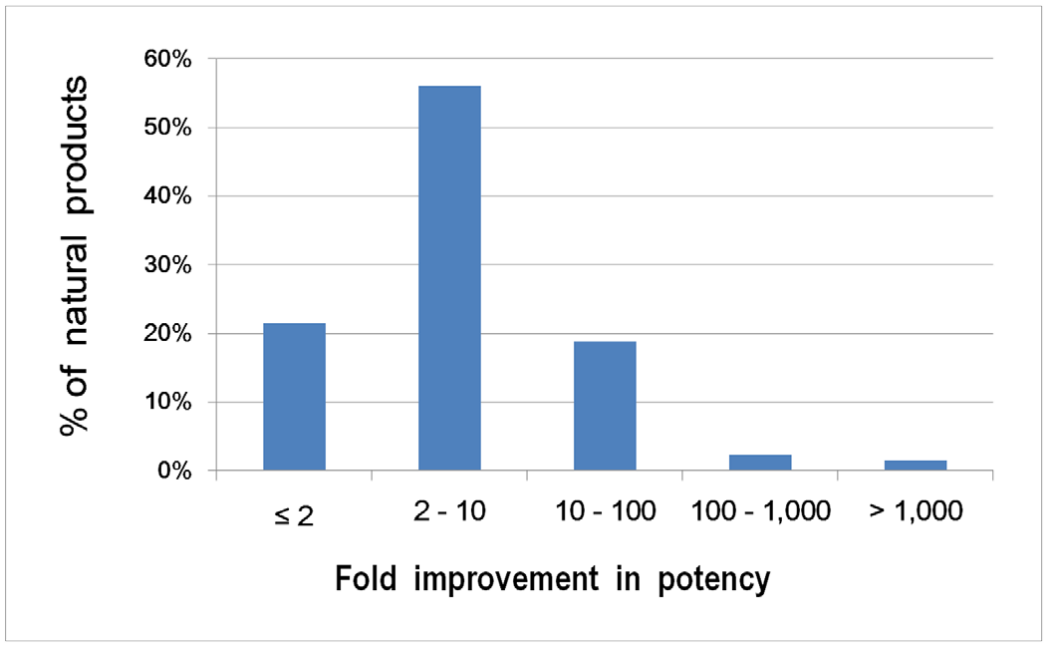
The potency improvement profile of the constituent NPs.

Based on Chou’s method [26], the levels of synergism in the NP combinations (**Figure 3**) were categorized into the levels of very strong synergism (CI<0.1), strong synergism (CI = 0.1–0.3), synergism (CI = 0.3–0.7), moderate synergism (CI = 0.7–0.85), slight synergism (CI = 0.85–0.90), nearly additive (CI = 0.90–1.10), slight antagonism (CI = 1.10–1.20), and moderate antagonism (CI = 1.20–1.45) respectively. Overall, 24% and 34% of the combinations are at the strong/very strong synergism and synergism levels, indicating that highly synergistic combinations can be formed at fair probabilities. **Figure 4** shows the potency improvement profile of the NPs in these combinations, in which 4% and 19% of the NPs exhibit >100-fold and 10–100 fold potency improvement respectively. This suggests that >10-fold potency improvement is achievable at moderate probabilities. These combinations are mostly composed of sub-potent NPs. There are only 6 potent NPs, and 1 and 3 combinations fully and partially composed of potent NPs. Synergism elevates the collective potencies of 5 fully sub-potent and 2 partially sub-potent combinations to drug level, and lifts the potency of 4 NPs in another 3 sub-potent combinations to drug level. Overall, the potencies of 22 (14.4%) sub-potent NPs and collective potencies of 7 (5.6%) sub-potent combinations are enhanced to drug level, suggesting that the individual and collective potencies of sub-potent NPs can be raised to drug level at moderate and low probabilities respectively.

### Potency Enhancing Molecular Modes of Natural Product Combinations

The molecular mechanisms of synergism of drug combinations [12] and NP combinations [8] can be studied from their MIPs. We conducted comprehensive literature search for identifying the targets and synergism-related MIPs of three NP combinations with collective potencies improved to drug levels, which identified 11 targets related to the reported therapeutic effects of these combinations and 72 MIPs likely contributing to the potency-enhancing modes (**Table S7)**. The targets and potency-enhancing MIPs of two of the NP combinations are also summarized in **Table 1** and **2**. Specific potency-enhancing molecular modes were identified. The potencies of the principal NP in these combinations are at or near drug potency level (IC50 = 0.8–1.1 µM, 0.94 µg/mL) probably due in part to the multi-target activities of each principal NPs (2, 4, 5 targets respectively). Network and activity analysis have shown that weak inhibition of multiple targets in related pathways may be more efficient than strong inhibition against a single target [42], [43]. The potencies of the companion NPs are substantially weaker (IC50 = 1.7–656 µM, 5.07–251 µg/mL). The potencies of all NPs in these combinations are significantly enhanced (mostly by >10-fold) by multi-target actions in modulating multiple regulators, partners and effectors of the primary targets of the active NPs (complementary actions), elevating intra-cellular bioavailability of the active NPs, and antagonizing the processes counteractive to the therapeutic effects of the active NPs (anti-counteractive actions).

**Table 1:** The targets and potency-enhancing synergistic molecular modes of the anticancer combination of Tetraarsenic tetrasulfide, Indirubin, and Tanshinone IIA (anticancer synergism reported).

Natural Product [Role in Combination] (Individual Potency){Dose ReductionIndex}	Target, Therapeutic Effect orResponse (reference inPubmed ID)	Effect type	Potency-Enhancing Synergistic Modes (reference in Pubmed ID)	Type of Synergism
Tetraarsenic tetrasulfide [Principal] (1.1 uM) {6.88}	Degraded PML-RAR to produce anticancer effect (18344322)	Growth inhibition,	Indirubin blocked RAR-STAT3 crosstalk (14959844) by reducing JAK/STAT3 signaling ((21207415). Tanshinone IIA reduced RAR (12069693) by hindering AR (22175694, 22281759, 21997969). These complement tetraarsenic tetrasulfide’s action on RAR	Complementary action
	Down-regulated CDK2 in NB4 and NB4-R2 cells (18344322)	Cell cycleregulation	Indirubin inhibited and reduced CDK2 (18344322) to complement tetraarsenic tetrasulfide’s action on CDK2	Complementary action
	Upregulated RING-type E3 ligase c-CBL and degraded BCR-ABL (21118980)	Growth inhibition		
	Transported into tumor cells by AQP9 (18344322)	Intracellular bioavailability	Indirubin and Tanshinone IIA upregulated APQ9 (18344322) to promote Tetraarsenic tetrasulfide’s cell entry	Intracellular bioavailability enhancement
	RARα reduction downregulated P53 and elevated Bcl-2 (10675490) to reduce apoptosis	Counteractiveaction	Tanshinone IIA activated p53 signaling (21997969) to reduce this counteractiveaction	Anti-counteractive action
Indirubin [Cooperative] (>3 uM) {>9.38}	Inhibited and reduced CDK2 to produce anticancer effect (18344322)	Cell cycleregulation	Tetraarsenic tetrasulfide reduced CDK2 (18344322) to complement indirubin’saction on CDK2	Complementary action
	Inhibited GSK3 to produce anticancereffect (21697283)	Growth inhibition		
	blocked VEGFR2 signaling (21207415) to reduce angiogenesis and apoptosis (14959844)	Growth,angiogenesisinhibition		
	Activated AhR (20951181) which activates RARα (16480812) to promote cancer	Counteractive action	Tetraarsenic tetrasulfide degraded PML-RAR (18344322) to alleviate this counteractive action	Anti-counteractive action
Tanshinone IIA [Cooperative ] (>3 uM) {>9.38}	Increased Bax/Bcl-2 ratio, caspase 3,reduced Bcl-2, mitochondrial membranepotential, MMPs, to promote apoptosis(21472292, 22002472, 22126901)	Apoptosis		
	Activated p53 signaling to promote anticancer effect (21997969)	Cell cycleregulation,apoptosis		
	Upregulated pP38 to enhance apoptosis (21165580)	Apoptosis		
	Reduced HER2, NF-κBp65, RARα activities (17451432) to promote anticancer effect (22246196),	Apoptosis, growth inhibition,		
	Reduced and antagonized AR andinduced apoptosis (22175694, 22281759,21997969)	Growth inhibition		
	pP38 upregulation (21165580) activated RARα (19078967, 20080953) to promote cancer	Counteractiveaction	Tetraarsenic tetrasulfide degraded PML-RAR (18344322) to alleviate this counteractive action	Anti-counteractive action
	Upregulated efflux transporters topromote Tanshinone IIA (a Pgp substrate)eflux (17504222, 20821829)	Intracellular bioavailability	Indirubin inhibit certain efflux pumps (20380543) which may reduce the efflux of Tanshinone IIA	Intracellular bioavailability enhancement

The detailed descriptions of the relevant molecular interaction profiles are in Table S7.

**Table 2:** The targets and potency-enhancing synergistic molecular modes of the anti-rotavirus combination of Theaflavin, Theaflavin-3-monogallate, Theaflavin-3′-monogallate, and Theaflavin-3,3′ digallate (anti-rotavirus synergism reported).

Natural Product [Role in Combination] (Individual Potency) { Dose Reduction Index}	Target, Therapeutic Effect or Response (reference in Pubmed ID)	Effect type	Potency-Enhancing SynergisticModes (reference in Pubmed ID)	Type of Synergism
Theaflavin [Principal] (0.943 ug/mL) {9.33}	Reduced JNK and P38 phosphorelation (21184129, 22111069) to block JNK andp38 mediated viral replication	Viral replication inhibition	Other 3 components block the redundant Cox2 and ERK viral replication pathwaysto complement Theaflavin’s activity	Complementary action
Theaflavin-3-monogallate [Cooperative ] (251.39 ug/mL) {2489}	Theaflavin-3-monogallate andtheaflavin-3′-monogallate mixturedownregulated Cox2 (11103814) to blockCox2 mediated viral replication andinfection (15331705, 17555580)	Viral replication inhibition	All 4 components collectively cover 4 redundant viral replication pathways to complement Theaflavin-3-monogallate’s activity	Complementary action
Theaflavin-3′-monogallate [Cooperative ] (5.07 ug/mL){50.2}	Theaflavin-3-monogallate andtheaflavin-3′-monogallate mixturedownregulated Cox2 (11103814) to blockCox2 mediated viral replicationand infection (15331705, 17555580),	Viral replication inhibition	All 4 components collectively cover 4 redundant viral replication pathways to complement Theaflavin-3′-monogallate’s activity	Complementary action
Theaflavin-3,3′ digallate [Cooperative] (5.51 ug/mL){54.6}	Reduced ERK phosphorelation(11511526) to block ERK mediatedviral replication (17689685),	Viral replication inhibition	Other 3 components block the redundant JNK, P38 and Cox2 viral replicationpathways to complement Theaflavin-3,3′digallate’s activity	Complementary action
	Blocked NFkB activation (16880762) to hinder NFkB and AkT mediated viralsurvival and growth (20392855)	Viral survival, growth inhibition		

The detailed descriptions of the relevant molecular interaction profiles are in Table S7.

Regulation of multiple regulators of the primary targets of principal NPs is important for elevating the collective potencies to drug level. In two combinations, 6 and 13 regulators of the primary targets of the principal NPs are modulated. In the third combination, each constituent NP targets one or two of the four redundant processes to collectively achieve therapeutic effects. These multi-target potency-enhancing modes are consistent with the reports that weak inhibition of multiple targets in related pathways may be more efficient than strong inhibition of a single target [42], [43]. In these combinations, complementary actions are achieved by modulating the expression, upstream regulators, crosstalk/redundant signaling, and substrates/effectors of the targets of individual NPs. Intra-cellular bioavailability of NPs are enhanced by inhibiting/downregulating efflux pumps and upregulating/activating cell-entry transporters. Anti-counteractive actions involve regulation of the pathways activated by the NPs that subsequently reduce the therapeutic effects of the NPs. Drug efficacies are reportedly reduced by network robustness [44], redundancy [45], crosstalk [46], and compensatory and neutralizing actions [47]. Our revealed potency-enhancing molecular modes provide useful clues for reducing these literature-reported negative effects by multi-targeted strategies.

Additional potency-enhancing mechanisms were studied by analyzing 8 and 26 MIPs in 2 and 9 combinations with the potency of the principal NP enhanced by >100-fold and 10–100 fold, and 16 MIPs of 5 combinations with the potency of a non-principal NP improved by >10-fold respectively (**Table S8, S9, 10**). The potency of individual NPs in 13 combinations is enhanced by a single mechanism: enhancement of the intra-cellular bioavailability of an active NP, which is an extensively-explored effective potency-enhancing strategy for those NPs with hindered intra-cellular bioavailability. In addition to actions on efflux and cell-entry transporters, intra-cellular bioavailability of NPs can be enhanced by regulating their metabolism, disrupting membrane structures, and the use of pro-drug NPs of better cell-entry abilities, The potency of individual NPs in the remaining 3 combination is enhanced by complementary and anti-counteractive modes similar to those of the three NP combinations with potencies improved to drug levels.

Although the potencies of some of the individual NPs in these combinations are significantly improved, none is elevated to drug level possibly due to low potencies of their principal NPs (44.6–800 µg/mL with one exception) and modulation of few regulators of the primary targets of the principal NPs. The success rate of assembling sub-potent NPs into drug-level potent combinations may be significantly improved by careful selection of principal NPs of sufficient potency (e.g. potency <10 µM) and the use of cooperative NPs that enhance the bioavailability and modulate the regulators, partners and effectors of the targets of the principal NPs.

### Influence of Individual Genetic Variations

Combinations of sub-potent NPs heavily rely on their synergistic actions for improved potencies, which typically involve collective modulation of a certain set of the primary targets and the corresponding secondary targets. Because of their heavy reliance on the modulation of the specific sets of secondary targets for achieving sufficiently improved potency, the level of potency improvement of synergistic NP combinations is expected to be sensitively influenced by the genetic variations that alter the expression and activity level of this set of targets [30]. **Table 3** shows the expression profiles of the primary targets and some of the potency-enhancing secondary targets of the selected NP combinations in specific patient groups. The primary targets are expressed in 42%–95% the patients and the secondary targets are expressed in 15%–100% of the patients in different patient groups. Significantly lower percentages of patients in each patient group are expected to have the right set of the targets co-expressed to make them responsive to a particular sub-potent NP combination. Multi-herb combinations have been frequently prescribed in personalized manner [48], [49] possibly out of the need for exploiting certain potency-enhancing modes active in specific patients.

**Table 3:** Expression profiles of the primary targets and some of the potency-enhancing secondary targets of the selected natural product combinations in specific patient groups.

Natural Product Combination	Target Type	Target	Target Expression Profile in Specific Patient Groups
Tetraarsenic tetrasulfide, Indirubin, and Tanshinone IIA	Primary target of the principal ingredient	PML-RAR	Present in 95% of APL patients (12506013)
	Secondary target for enhancingthe potency of the principalingredient	STAT3	Aberrantly activated in some APL patients (11929748), activated in 71% of AML patients (9679986)
Theaflavin, Theaflavin-3-monogallate, Theaflavin-3′-monogallate, andTheaflavin-3,3′ digallate	Primary target of the principal ingredient	JNK	Expressed in 100% of patients with chronic obstructive pulmonary disease (20699612), pJNK expressed in 100% of multiple trauma patients (22677613)
		P38	Expressed in 82% patients with sepsis-induced acute lung injury (17581740), pP38 expressed in 38% of multiple trauma patients (22677613)
	Secondary target involved in the alternative signaling thatsubstitute the targeted pathway ofthe principal ingredient	Cox2	Expressed in 100% of HBV (15218507) and 100% of HCV (17845691) patients, elevated in 100% of patients with HCV-induced chronic liver disease (18092051)
		ERK	pERK expressed in 15% of colorectal carcinoma (17149612), 39% of mucoepidermoid carcinomas (12937136), 70% of breast cancer (15928662), 79% of mucoepidermoid carcinoma (20664595) patients
Wedelolactone, indole-3-carboxylaldehyde, luteolin, apigenin	Primary target of the principal ingredient	AR	Expressed in 59% of prostate cancer (22500161), 56%–63% of breast cancer (18946753, 22471922), 80% of benign urothelium (22221549), 50% of benign stroma (22221549), 42%–71% of bladder cancer (22221549) patients
	Secondary target for enhancingthe potency of the principalingredient	c-Src	Expressed in 55% of metastatic breast cancer (22716210), 74% of bladder cancer (22353809), 28% of hormone refractory prostate cancer patients (19447874)
		FGF1R	Expressed in 69%–74% of prostate cancer (17607666), 99%–100% of breast cancer (9865904, 9756721) patients
		topoisomeraseII	Highly expressed and amplified in 50% and 5%–7% of breast cancer (22240029, 22555090), 31% and 26% of advanced prostate cancer (17363613), 20% and 1.5% of bladder cancer (11304849, 14566826) patients
		CK2	Expressed in the bone marrow of 28% of the patients with transitional cell carcinoma (17977715)
		EGFR	Expressed in 41% of prostate cancer (22500161), 25% of breast cancer (22562124), 33% of triple negative breast cancer (22481575), 66%–96% of bladder cancer (16685269, 19171060) patients
		HER2	Expressed in 1.5%–24% of prostate cancer (19207111, 22500161), 8%–31% breast cancer (10550311, 11344480, 22562124), 62%–98% of bladder cancer (15839918, 16685269) patients
		NF-kB	Expressed in 53% of prostate cancer (21156016), 79% of bladder urothelial carcinoma (18188593), active NF-kB present in 4.4%–43% of breast cancer (16740744) patients
		AkT	pAkT expressed in 45% prostate cancer (19389013) and 33% breast cancer (16464571), highly expressed in 2.6%–14.3% of patients with urothelial carcinoma of the urinary bladder (21707707)
		P53	Expressed in 22%–28% breast cancer (11344480), Overexpressed in 36% of bladder cancer (19171060) patients

### Concluding Remarks

Our analysis indicates the possibility of synergistically assembling sub-potent NPs into drug-level potent combinations, which can be achieved at low probabilities by the exploration of specific potency-enhancing modes that combine multi-target actions of the principal NPs of sufficient potency (typically within 10-fold range of drug potency levels) against specific disease processes with the enhancement of their bioavailability and/or the modulation of the regulators, effectors and counteractive elements of their targets. The low probabilities for assembling sub-potent NPs into drug-level potent combinations may arise from the difficulties in finding the right combination of NPs with sufficient potency and the appropriate and complementary potency-enhancing MIPs. Moreover, synergistic actions typically involve interactions with multiple sites, targets and pathways which are sensitively influenced by genetic [50], environmental [13], behavioral [51], and scheduling [52] profiles. NP combinations and related therapeutics may be better designed, applied and studied in personalized and environment-dependent manners [53], [54]. The efforts in the exploration of NP combinations can be facilitated by expanded knowledge in the activities of NPs [55], MIPs of NPs [8], disease regulations, and potency-enhancing molecular modes that synergistically target key positive [56] and negative [57] regulatory nodes of therapeutic efficacies, and collectively modulate anti-targets and counter-targets [58], compensatory and neutralizing actions [47], [59], and transporter and enzyme mediated pharmacokinetic activities [60].

## Supporting Information

Table S1 Cell-based inhibitory activity data of 88 anticancer drugs.
(PDF)

Table S2 Cell-based microbial inhibitory activity data of 102 antimicrobial drugs.
(PDF)

Table S3 Cell-based inhibitory activity values of 650 anticancer natural products.
(PDF)

Table S4 Cell-based microbial inhibitory activity values of 609 antimicrobial natural products.
(PDF)

Table S5 Cell-based microbial inhibitory activity values of 99 antimicrobial natural product extracts.
(PDF)

Table S6 List of 124 synergistic natural product combinations with available cell-based potency data.
(XLSX)

Table S7 Targets and potency-enhancing synergistic molecular modes in 3 fully or partially sub-potent natural product combinations with group potencies improved to drug levels.
(PDF)

Table S8 Targets and potency-enhancing molecular interaction modes in 2 fully sub-potent natural product combinations with potencies of the principal component increased by >100 fold.
(PDF)

Table S9 Targets and potency-enhancing molecular interaction modes in 9 fully sub-potent natural product combinations with potencies of the principal component increased by 10–100 fold.
(PDF)

Table S10 Targets and potency-enhancing molecular interaction modes in 5 fully sub-potent natural product combinations with potencies of a non-principal component increased by 10–100 fold.
(PDF)
