# Supplementary material for: What Does It Take to Synergistically Combine Sub-Potent Natural Products into Drug-Level Potent Combinations?
Source: PLoS One. 2012 Nov 28;7(11):e49969. doi: 10.1371/journal.pone.0049969 (PMC3509152; doi:10.1371/journal.pone.0049969)
Supplement: Table S1 — Cell-based inhibitory activity data of 88 anticancer drugs. (PDF) [file pone.0049969.s001.pdf]

Supplementary Table S1 Cell-based inhibitory activity data of 88 anticancer drugs. For drugs with multiple cancer cell-line inhibition data, the best activity is listed.

**Drug**	**Cell line**	**GI50/IC50 (nM)**	**Reference (Pubmed ID)**
5-azacytidine	NCI-H460	147.9	20442306
5-fluorouracil	OVCAR-3	21.4	20442306
6-Mercaptopurine	K-562	354.8	20442306
Actinomycin D	SR	0.0019	20442306
Altretamine	HOP-18	60534.1	NCI standard agent database
Anastrozole	SK-MEL-2	100.0	20442306
Arsenic trioxide	CCRF-CEM	512.9	20442306
Bendamustine	MOLT-4	1148.2	20442306
Bleomycin	MALME-3M	2.9	20442306
Bortezomib	RPMI-8226	0.2	20442306
Busulfan	LOX IMVI	6025.6	20442306
Capcitebine	SK-MEL-2	10.0	20442306
Carboplatin	KLE	240.0	8123477
Carmustine	SW 1783	1524.1	NCI standard agent database
Chlorambucil	SR	812.8	20442306
Cisplatin	SR	77.6	20442306
Cladribine	HL60	8.7	9845378
Clofarabine	CCRF-CEM	10.0	20442306
Cyclophosphamide	COLO 746	25.0	NCI standard agent database
Cytarabine HCl	CCRF-CEM	6.0	20442306
Dacarbazine	HL-60(TB)	4168.7	20442306
Dasatinib	K-562	10.0	20442306
Daunorubicin	MOLT-4	2.8	20442306
Delta-1-testololactone	HOP-19	18793.2	NCI standard agent database
Dimethyltestosterone	RPMI-8226	7585.8	20442306
Docetaxel	NCI-H522	0.1	20442306
Doxorubicin	MOLT-4	10.0	20442306
Dromostanolone propionate	HOP-92	407.4	20442306
Epirubicin	SR	10.0	20442306
Erlotinib	EKVX	53.7	20442306
Estramustine	CCRF-CEM	25.1	20442306
Ethacrynic acid	Primary Chronic Lymphocytic Leukemia cells	8560.0	20011538
Ethinyl estradiol	U251	7762.5	20442306
Etoposide	MOLT-4	195.0	20442306

Everolimus	RPMI-8226	10.0	20442306
Exemestane	SK-MEL-2	616.6	20442306
Floxuridine	HCC-2998	1.0	20442306
Fludarabine	CCRF-CEM	1584.9	20442306
Fulvestrant	MCF7	10.0	20442306
Gefitinib	MDA-MB-468	10.0	20442306
Gemcitabine	CCRF-CEM	10.0	20442306
Hydroxyurea	COLO 746	1977.0	NCI standard agent database
Idarubicin HCl	NCI-H460	0.56	20442306
Ifosfamide	Fibromatosis tumour cells	6230.0	18521267
Imatinib	K-562	23.4	20442306
Irinotecan	SR	234.4	20442306
Ixabepilone	NSC 747973	0.01	20442306
Lapatinib	MDA-MB-468	10.0	20442306
Lenalidomide	CCRF-CEM	100000.0	20442306
Letrozole	BT-549	12.0	20442306
Lomustine	SR	10.0	20442306
Megestrol acetate	RPMI-8226	1380.4	20442306
Melphalan	MCF7	300.0	3422442
Methotrexate	HCT-116	1.3	20442306
Mithramycin	NSC 24559	13.0	20442306
Mitomycin C	NCI-H460	17.8	20442306
Mitotane	BT-549	1584.9	20442306
Mitoxantrone	MCF7	4.3	20442306
Mitramycin	MCF7	0.011	20442306
Naldrolone	EKVX	2238.7	20442306
Nelarabine	SK-MEL-2	10.0	20442306
Nilotinib	K-562	10.0	20442306
Nitrogen mustard	CAKI-1	107.2	20442306
Oxaliplatin	NCI/ADR-RES	31.6	20442306
Paclitaxel	SK-MEL-2	0.93	20442306
Pemetrexed	HCC-2998	10.0	20442306
Pentostatin	SHP-77	134276.5	NCI standard agent database
Pipobroman	SR	8128.3	20442306
Quinacrine HCl	MOLT-4	512.9	20442306
Raloxifene	MCF7	87.1	20442306
Romidepsin	MDA-MB-435	0.01	20442306
Sorafenib	KM12	776.2	20442306
Streptozoticin	H727	125000.0	21239354
sunitinib	Human AML cell line	7.0	18483300
Tamoxifen	T47D	470.0	16323060

Temsirolimus	MALME-3M	0.20	20442306
Teniposide	SR	11.7	20442306
Thioguanine	CCRF-CEM	169.8	20442306
ThioTEPA	SR	549.5	20442306
Topotecan	SNB-75	2.5	20442306
Toremifene	MCF7	1584.9	20442306
Triethylenemelamine	NCI-H460	407.4	20442306
Uracil nitrogen mustard	HL-60(TB)	1445.4	20442306
Valrubicin	NCI-H460	21.4	20442306
Vinblastine sulfate	SR	0.0025	20442306
Vincristine sulfate	RPMI-8226	1.0	20442306
Vinorelbine tartrate	CCRF-CEM	10.0	20442306
Vorinostat	NCI/ADR-RES	93.3	20442306
