# Supplementary material for: What Does It Take to Synergistically Combine Sub-Potent Natural Products into Drug-Level Potent Combinations?
Source: PLoS One. 2012 Nov 28;7(11):e49969. doi: 10.1371/journal.pone.0049969 (PMC3509152; doi:10.1371/journal.pone.0049969)
Supplement: Table S2 — Cell-based microbial inhibitory activity data of 102 antimicrobial drugs. (PDF) [file pone.0049969.s002.pdf]

Supplementary Table S2 Cell-based microbial inhibitory activity data of 102 antimicrobial drugs. For drugs with multiple antimicrobial MIC data, the best activity is listed

**Drug**	**Against Species**	**MIC (μg/mL)**	**Reference (Pubmed ID)**
Amikacin	Escherichia coli ATCC10536	0.5	11168187
Amoxicillin	S. pneumoniae ATCC 49619	0.06	11420333
Ampicillin	Staphylococcus aureus ATCC9144	0.06	11168187
Azithromycin	S. pneumoniae ATCC 49619	0.12	11420333
Azlozillin	S. aureus NCTC 6571	0.25	11420333
Aztreonam	E. coli NCTC 10418	0.03	11420333
Balofloxacin	Mycobacteri uM tuberculosis MTB 09710 clinical isolate	16	20493593
Capreomycin	M. tuberculosis 07-10680	0.5	20237102
Carbenicillin	Staphylococcus aureus 209P	0.39	16141558
Cefaclor	Escherichia coli ATCC10536	1	11168187
Cefamandole	MSSA MLSB-S	0.25	10858332
Cefazolin	Staphylococcus aureus ATCC29213	0.5	11168187
Cefepime	Escherichia coli MC4100 strain	0.015	17470659
Cefixime	H. influenzae NCTC 11931	0.03	11420333
Cefonicid	Escherichia coli ATCC25922	0.5	11168187
Cefoperazone	Escherichia coli ATCC25922	0.25	11168187
Cefotaxime	Cefotaxime susceptible, erythromycin susceptible 1 (364.0344)	0.016	12604560
Cefotetan	Escherichia coli ATCC10536	0.06	11168187
Cefoxitin	Escherichia coli ATCC25922	2	11168187
Cefpirome	E. coli NCTC 10418	0.03	11420333
Cefpodoxime	Amps and AmprBLP isolates	0.08	2610499
Cefsulodin	Pseudomonas aeruginosa ATCC25668	2	11168187
Ceftazidime	Escherichia coli ATCC10536	0.06	11168187
Ceftibuten	Amps and AmprBLP isolates	0.08	2610499
Ceftizoxime	Escherichia coli ATCC10536	0.008	11168187
Ceftriaxone	Escherichia coli ATCC10536	0.03	11168187
Cefuroxime	S. pneumoniae ATCC 49619	0.25	11420333
Cephadroxil	S. aureus NCTC 6571	1	11420333
Cephalexin	Staphylococcus aureus ATCC9144	1	11168187
Cephaloridine	S. aureus NCTC 6571	0.06	11420333
Cephalothin	Staphylococcus aureus ATCC29213	0.25	11168187
Cephradine	Staphylococcus aureus ATCC9144	2	11168187
Cethromycin	Ent. Faecalis ATCC 29212	0.008	11420333

Chloramphenicol	Escherichia coli ATCC10536	2	11168187
Ciprofloxacin	N. gonorrhoeae ATCC 49226	0.004	11420333
Clarithromycin	S. pneumoniae ATCC 49619	0.03	11420333
Clindamycin	Staphylococcus aureus ATCC9144	0.06	11168187
Colistin	Escherichia coli ATCC10536	0.5	11168187
Dalfopristin	MSSA MLSB-S	1	10858332
Dipropofol	Staphylococcus aureus 209P	1.56	16141558
Dirythromycin	S. aureus NCTC 6571	1	11420333
Doxycycline monohydrate	Streptococcus pyogenes	0.12	217546627
Enoxacin	Escherichia coli ATCC25922	0.125	11168187
Ertapenem	E. coli NCTC 10418	0.008	11420333
Erythromycin	Penicillin susceptible, erythromycin susceptible 12 (1014)	0.062	12604560
Ethambutol	M. tuberculosis	2	19633001
Faropenem	S. pneumoniae ATCC 49619	0.06	11420333
Fleroxacin	Escherichia coli ATCC25922	0.06	11168187
Flucloxacillin	MSSA MLSB-S	0.06	10858332
Flumequine	E. coli NCTC 10418	2	11420333
Fosfomycin	E. coli NCTC 10418	4	11420333
Fusidic acid	S. aureus NCTC 6571	0.06	11420333
Garenoxacin	P. aeruginosa A22379	1	12127712
Gatifloxacin	E. coli NCTC 10418	0.015	11420333
Gatioxacin	Escherichia coli ATCC10536	0.015	11168187
Gemifloxacin	N. gonorrhoeae ATCC 49226	0.002	11420333
Gentamicin	MSSA C-MLSB-R	0.06	10858332
Grepafloxacin	H. influenzae ATCC 49247	0.004	11420333
Imipenem	MSSA MLSB-S	0.01	10858332
Isoniazid	M. tuberculosis	0.06	19633001
Kanamycin	M. tuberculosis 07-10680	0.125	20237102
Levofloxacin	H. influenzae ATCC 49247	0.015	11420333
Linezolid	Staphylococcus aureus ATCC9144	0.5	11168187
Lomefloxacin	S. aureus NCTC 6571	0.5	11420333
Loracarbef	Escherichia coli ATCC10536	0.5	11168187
Mecillinam	E. coli NCTC 10418	0.12	11420333
Meropenem	E. coli ATCC 25922	0.008	11420333
Methicillin	Staphylococcus aureus ATCC29213	1	11168187
Metronidazole	B. fragilis NCTC 9343	0.5	11420333
Mezlocillin	Staphylococcus aureus ATCC9144	0.5	11168187
Moxalactam	Escherichia coli ATCC10536	0.03	11168187
Moxifloxacin	N. gonorrhoeae ATCC 49226	0.004	11420333
Mupirocin	S. aureus ATCC 29213	0.12	11420333
Nalidixic acid	H. influenzae ATCC 49247	1	11420333

Neomycin	S. aureus NCTC 6571	0.12	11420333
Netilmicin	Staphylococcus aureus ATCC9144	0.25	11168187
Nitrofurantoin	Escherichia coli ATCC10536	4	11168187
Norfloxacin	Escherichia coli ATCC25922	0.06	11168187
Ofloxacin	Escherichia coli ATCC25922	0.03	11168187
Oxacillin	Staphylococcus aureus ATCC29213	0.25	11168187
Pefloxacin	Escherichia coli ATCC10536	0.06	11168187
Penicillin	Penicillin susceptible, erythromycin susceptible 1 (364.0344)	0.016	12604560
Piperacillin	Staphylococcus aureus ATCC9144	0.25	11168187
Quinupristin	MSSA MLSB-S	1	10858332
Rifampicin	Staphylococcus aureus ATCC9144	0.004	11168187
Roxithromycin	S. pneumoniae ATCC 49619	0.12	11420333
Rufloxacin	E. coli NCTC 10418	0.5	11420333
Sparfloxacin	H. influenzae ATCC 49247	0.002	11420333
Streptomycin	M. tuberculosis H37Rv	0.25	20237102
Sulfamethoxazole	Escherichia coli ATCC10536	16	11168187
Sulfisoxazole	Escherichia coli ATCC25922	16	11168187
Sulphonamide	E. coli NCTC 10418	16	11420333
Teicoplanin	Enterococcus faecalis ATCC29212	0.125	11168187
Telithromycin	Ent. Faecalis ATCC 29212	0.008	11420333
Temocillin	Escherichia coli ATCC10536	2	11168187
Tetracycline	S. aureus NCTC 6571	0.06	11420333
Ticarcillin	Staphylococcus aureus ATCC9144	0.5	11168187
Tobramycin	S. aureus NCTC 6571	0.12	11420333
Trimethoprim	E. coli NCTC 10418	0.12	11420333
Trovafloxacin	H. influenzae ATCC 49247	0.002	11420333
Vancomycin	S. pneumoniae ATCC 49619	0.25	11420333
Viomycin	M. tuberculosis 07-10680	0.5	20237102
