# Supplementary material for: What Does It Take to Synergistically Combine Sub-Potent Natural Products into Drug-Level Potent Combinations?
Source: PLoS One. 2012 Nov 28;7(11):e49969. doi: 10.1371/journal.pone.0049969 (PMC3509152; doi:10.1371/journal.pone.0049969)
Supplement: Table S3 — Cell-based inhibitory activity values of 650 anticancer natural products. (PDF) [file pone.0049969.s003.pdf]

Supplementary Table S3 Cell-based inhibitory activity values of 650 anticancer natural products

Species	Natural product	Cell line	GI50/IC50 (nM)	Reference (Pubmed ID)
green tea	(−)-Epigallocatechin-3-Gallate	cancer cells	9010	9525275
the root barks of some Rutaceae plants	Atalaphyllinine	TGBC-11TKB	1430	10217715
the root barks of some Rutaceae plants	Des-N-Methylnoracronycine	TGBC-11TKB	2070	10217715
	5-Hydroxy-N-Methylseverifoline	TGBC-11TKB	2130	10217715
	Atalaphyllidine	TGBC-11TKB	2830	10217715
the root barks of some Rutaceae plants	5-Hydroxynoracronycine	TGBC-11TKB	16200	10217715
	Grandisine I	TGBC-11TKB	19300	10217715
Tabebuia impetiginosa	2-Acetyl-8-Hydroxynaphtho[2,3-β]Furan-4,9-Dione	HaCaT	300	10479319
Tabebuia impetiginosa	Lapacho Compound 6	HaCaT	500	10479319
Tabebuia impetiginosa	β-Lapachone	HaCaT	700	10479319

Tabebuia impetiginosa	2-Ethyl-6-Hydroxynaphtho[1,2-β]Furan-4,5-Dione	HaCaT	900	10479319
Tabebuia impetiginosa	2-Ethyl-8-Hydroxynaphtho[2,3-β]Furan-4,9-Dione	HaCaT	3000	10479319
Tabebuia impetiginosa	Lapacho Compound 5	HaCaT	3700	10479319
Tabebuia impetiginosa	α-Lapachone	HaCaT	10000	10479319
	7-Substituted Diaminofluoroflavone	MFC-7	10	11562277
	Compound 22B	cytotoxicity	2000	11562277
Bufo gargarizans Cantor, Bufo melanostrictus Schneider	1β-Hydroxybufalin	SF268	11	11575946
Bufo gargarizans Cantor, Bufo melanostrictus Schneider	19-Oxobufalin	DU145	11.5	11575946
Bufo gargarizans Cantor, Bufo melanostrictus	12β-Hydroxycinobufagin	HL60	25	11575946

Schneider				
Bufo gargarizans Cantor, Bufo melanostrictus Schneider	Bufalin	HL60	25	11575946
Bufo gargarizans Cantor, Bufo melanostrictus Schneider	Bufotalin	HL60	25	11575946
Bufo gargarizans Cantor, Bufo melanostrictus Schneider	Cinobufagin	HL60	25	11575946
Bufo gargarizans Cantor, Bufo melanostrictus Schneider	Telocinobufagin	HL60	25	11575946
Bufo gargarizans Cantor, Bufo melanostrictus Schneider	12 β -Hydroxyresibufogenin	KM20L2	30	11575946

Bufo gargarizans Cantor, Bufo melanostrictus Schneider	Gamabufotalin	HL60	35	11575946
Bufo gargarizans Cantor, Bufo melanostrictus Schneider	5β-Hydroxybufotalin	DU145	60	11575946
Bufo gargarizans Cantor, Bufo melanostrictus Schneider	Desacetylbufotalin	HL60	62.5	11575946
Bufo gargarizans Cantor, Bufo melanostrictus Schneider	6α-Hydroxycinobufagin	HL60	95	11575946
Bufo gargarizans Cantor, Bufo melanostrictus Schneider	Cinobufotalin	HL60	117.5	11575946
Bufo gargarizans Cantor, Bufo	Resibufaginol	SF268	625	11575946

melanostrictus Schneider				
Bufo gargarizans Cantor, Bufo melanostrictus Schneider	3β-Formyloxyresibufogenin	SF268	950	11575946
Bufo gargarizans Cantor, Bufo melanostrictus Schneider	Desacetylcinobufagin	Kb	1100	11575946
Bufo gargarizans Cantor, Bufo melanostrictus Schneider	Desacetylcinobufaginol	HL60	1225	11575946
Bufo gargarizans Cantor, Bufo melanostrictus Schneider	Resibufogenin	HL60	1250	11575946
Bufo gargarizans Cantor, Bufo melanostrictus Schneider	19-Oxodesacetylcinobufagin	Kb	1625	11575946

Bufo gargarizans Cantor, Bufo melanostrictus Schneider	Desacetylciobufotalin	HL60	10750	11575946
Bufo bufo gargarizans	Digitoxin	prostate cancer cell lines LNCaP, DU145 and PC4	1000	11586264
Bufo bufo gargarizans	Digoxin	prostate cancer cell lines LNCaP, DU145 and PC3	1000	11586264
Bufo bufo gargarizans	Ouabain	prostate cancer cell lines LNCaP, DU145 and PC5	10000	11586264
Tithonia diversifolia	Sesquiterpenoid Compound 5	Col2	1750	11975495
Tithonia diversifolia	Sesquiterpenoid	Col2	4250	11975495

	Compound 6			
Tithonia diversifolia	Sesquiterpenoid Compound 4	Col2	14750	11975495
Tithonia diversifolia	Sesquiterpenoid Compound 1	HL-60	25000	11975495
Tithonia diversifolia	Sesquiterpenoid Compound 3	HL-60	25000	11975495
Tithonia diversifolia	Sesquiterpenoid Compound 7	Col2	47250	11975495
Tithonia diversifolia	Sesquiterpenoid Compound 2	HL-60	93500	11975495
C. vincetoxicum	6-O-Desmethylantofine	KB-3-1	7	12350151
C. vincetoxicum	Antofine	KB-V1	14	12350151
C. vincetoxicum	7-Desmethyltylophorine	KB-3-1	15	12350151
C. vincetoxicum	Isotylocrebrine	KB-3-1	43	12350151
C. vincetoxicum	Tylophorine	KB-V1	173	12350151
C. vincetoxicum	6-O-Desmethylsecoantofine	KB-3-1	403	12350151
C. vincetoxicum	Secoantofine	KB-V1	2440	12350151
Strychnos	Longicaudatine	HCT-116	4930	12398531
Strychnos	Ochrolifuanine E	HCT-116	5700	12398531

Strychnos	Sungucinec	HCT-116	6200	12398531
Strychnos	Isostrychnopentamine	HCT-116	7470	12398531
Strychnos	Dihydrousambarensinec	HCT-116	12000	12398531
Strychnos	Matopensine	HCT-116	12000	12398531
Strychnos	Strychnogucine Bc	HCT-116	15000	12398531
Strychnos	Ochrolifuanine A	HCT-116	16100	12398531
Strychnos	18-Hydroxyisosungucine c	HCT-116	16200	12398531
Strychnos	16-Methoxyisomatopensine	HCT-116	24500	12398531
Strychnos	Chloroquine	HCT-116	33700	12398531
Strychnos	18-Hydroxymatopensine	HCT-116	42500	12398531
soy	Genistein	human ovarian carcinoma cells	32000	12504192
soy	Quercetin	human ovarian carcinoma cells	66000	12504192
Vepris punctata	5-Methoxymaculine	A2780	7000	12713408
Vepris punctata	Flindersiamine	A2780	8250	12713408
Vepris punctata	4,5,6,7,8-Pentamethoxyf	A2780	8500	12713408

	uroquinoline			
Vepris punctata	5,8-Dimethoxymaculine	A2780	8750	12713408
Vepris punctata	Kokusaginine	A2780	8750	12713408
Vepris punctata	Maculine	A2780	10000	12713408
Vepris punctata	Skimmianine	A2780	10500	12713408
plant species	Plant-Derived Triterpenoid Compound 96	P-388	4	12871157
plant species	Plant-Derived Triterpenoid Compound 85	KB	14	12871157
plant species	Plant-Derived Triterpenoid Compound 95	P-388	20	12871157
plant species	Plant-Derived Triterpenoid Compound 94	P-388	45	12871157
plant species	Plant-Derived Triterpenoid Compound 97	P-388	53	12871157
plant species	Plant-Derived	LNCaP	71	12871157

	Triterpenoid Compound 86			
plant species	Plant-Derived Triterpenoid Compound 54	P-388	98	12871157
plant species	Plant-Derived Triterpenoid Compound 98	KB	160	12871157
plant species	Plant-Derived Triterpenoid Compound 109	K562 cell	170	12871157
plant species	Plant-Derived Triterpenoid Compound 104	K562 cell	200	12871157
plant species	Plant-Derived Triterpenoid Compound 62	HeLa	200	12871157
plant species	Plant-Derived Triterpenoid Compound 108	K562 cell	270	12871157
plant species	Plant-Derived Triterpenoid Compound	P-388	270	12871157

	59			
plant species	Plant-Derived Triterpenoid Compound 50	A549 lung adenocarcinoma	460	12871157
plant species	Plant-Derived Triterpenoid Compound 89	PC-3	574	12871157
plant species	Plant-Derived Triterpenoid Compound 63	Hep-2	600	12871157
plant species	Plant-Derived Triterpenoid Compound 107	K562 cell	700	12871157
plant species	Plant-Derived Triterpenoid Compound 56	LNCaP	700	12871157
plant species	Plant-Derived Triterpenoid Compound 106	K562 cell	720	12871157
plant species	Plant-Derived Triterpenoid Compound	PC-3	760	12871157

	88			
plant species	Plant-Derived Triterpenoid Compound 105	K562 cell	770	12871157
plant species	Plant-Derived Triterpenoid Compound 55	L-1210	780	12871157
plant species	Plant-Derived Triterpenoid Compound 47	HTC-15	800	12871157
plant species	Plant-Derived Triterpenoid Compound 11	Co-115	821	12871157
plant species	Plant-Derived Triterpenoid Compound 30	MCF-7	900	12871157
plant species	Plant-Derived Triterpenoid Compound 31	MCF-8	900	12871157
plant species	Plant-Derived Triterpenoid Compound	HT-29	940	12871157

	90			
plant species	Plant-Derived Triterpenoid Compound 46	A549 lung adenocarcinoma	1000	12871157
plant species	Plant-Derived Triterpenoid Compound 7	KB	1100	12871157
plant species	Plant-Derived Triterpenoid Compound 83	KB	1200	12871157
plant species	Plant-Derived Triterpenoid Compound 67	MDA-MB-231	1230	12871157
plant species	Plant-Derived Triterpenoid Compound 49	HTC-15	1300	12871157
plant species	Plant-Derived Triterpenoid Compound 52	A549 lung adenocarcinoma	1400	12871157
plant species	Plant-Derived Triterpenoid Compound	HepG2	1430	12871157

	27			
plant species	Plant-Derived Triterpenoid Compound 28	P-388	1500	12871157
plant species	Plant-Derived Triterpenoid Compound 57	HeLa	1500	12871157
plant species	Plant-Derived Triterpenoid Compound 41	MDA-MB-231	1600	12871157
plant species	Plant-Derived Triterpenoid Compound 92	PC-3	1780	12871157
plant species	Plant-Derived Triterpenoid Compound 60	U373	1800	12871157
plant species	Plant-Derived Triterpenoid Compound 100	CCRF-CEM	1980	12871157
plant species	Plant-Derived Triterpenoid Compound	HeLa	2000	12871157

	61			
plant species	Plant-Derived Triterpenoid Compound 20	HeLa	2100	12871157
plant species	Plant-Derived Triterpenoid Compound 48	HTC-15	2100	12871157
plant species	Plant-Derived Triterpenoid Compound 93	P-388	2200	12871157
plant species	Plant-Derived Triterpenoid Compound 87	P-388	2500	12871157
plant species	Plant-Derived Triterpenoid Compound 51	A549 lung adenocarcinoma	2590	12871157
plant species	Plant-Derived Triterpenoid Compound 42	A549 lung adenocarcinoma	2600	12871157
plant species	Plant-Derived Triterpenoid Compound	P-388	3100	12871157

	73			
plant species	Plant-Derived Triterpenoid Compound 17	HeLa	3300	12871157
plant species	Plant-Derived Triterpenoid Compound 45	KB	3740	12871157
plant species	Plant-Derived Triterpenoid Compound 12	human large-cell lung carcinoma	4000	12871157
plant species	Plant-Derived Triterpenoid Compound 80	P-388	4600	12871157
plant species	Plant-Derived Triterpenoid Compound 91	MCF-7	4720	12871157
plant species	Plant-Derived Triterpenoid Compound 53	HEPA-2B	4900	12871157
plant species	Plant-Derived Triterpenoid Compound	KB	4910	12871157

	44			
plant species	Plant-Derived Triterpenoid Compound 64	HT-29	5310	12871157
plant species	Plant-Derived Triterpenoid Compound 2	26-L5	5420	12871157
plant species	Plant-Derived Triterpenoid Compound 65	P-388	5700	12871157
plant species	Plant-Derived Triterpenoid Compound 69	P-388	6000	12871157
plant species	Plant-Derived Triterpenoid Compound 84	P-388	6500	12871157
plant species	Plant-Derived Triterpenoid Compound 40	MDA-MB-231	6600	12871157
plant species	Plant-Derived Triterpenoid Compound	P-388	7200	12871157

	82			
plant species	Plant-Derived Triterpenoid Compound 4	Erlich ascites	7500	12871157
plant species	Plant-Derived Triterpenoid Compound 34	HCT-15	7900	12871157
plant species	Plant-Derived Triterpenoid Compound 13	U373	8000	12871157
plant species	Plant-Derived Triterpenoid Compound 72	P-388	8900	12871157
plant species	Plant-Derived Triterpenoid Compound 79	P-388	9200	12871157
plant species	Plant-Derived Triterpenoid Compound 1	26-L5	9540	12871157
plant species	Plant-Derived Triterpenoid Compound	P-388	10000	12871157

	81			
plant species	Plant-Derived Triterpenoid Compound 66	P-388, A549, HT-29, MEL-28	11400	12871157
plant species	Plant-Derived Triterpenoid Compound 70	P-388, A549	11400	12871157
plant species	Plant-Derived Triterpenoid Compound 102	HepG2	11700	12871157
plant species	Plant-Derived Triterpenoid Compound 68	HeLa	11700	12871157
plant species	Plant-Derived Triterpenoid Compound 29	KB	12000	12871157
plant species	Plant-Derived Triterpenoid Compound 16	UMR106	14000	12871157
plant species	Plant-Derived Triterpenoid Compound	M-14	14600	12871157

	21			
plant species	Plant-Derived Triterpenoid Compound 3	Erlich ascites	17000	12871157
plant species	Plant-Derived Triterpenoid Compound 74	KB	20800	12871157
plant species	Plant-Derived Triterpenoid Compound 71	KB	24400	12871157
plant species	Plant-Derived Triterpenoid Compound 32	HCT-15	26500	12871157
plant species	Plant-Derived Triterpenoid Compound 24	HL-60 and CCRF-CEM	31000	12871157
plant species	Plant-Derived Triterpenoid Compound 35	HCT-15	32400	12871157
plant species	Plant-Derived Triterpenoid Compound	B16	33000	12871157

	99			
plant species	Plant-Derived Triterpenoid Compound 101	HepG2	35500	12871157
plant species	Plant-Derived Triterpenoid Compound 33	KB	35700	12871157
plant species	Plant-Derived Triterpenoid Compound 22	SK-OV-3	37600	12871157
plant species	Plant-Derived Triterpenoid Compound 8	MEL-2	39400	12871157
plant species	Plant-Derived Triterpenoid Compound 6	CCL-81	90100	12871157
plant species	Plant-Derived Triterpenoid Compound 5	CCL-81	163000	12871157
green tea	(-)-Epicatechin Gallate	HeLa cells	153000	12906756
Streptocaulon	Cardenolide Compound	HT-1080	54	14640513

juventas	13			
Streptocaulon juventas	Cardenolide Compound 15	HT-1080	55	14640513
Streptocaulon juventas	Cardenolide Compound 17	HT-1080	92	14640513
Streptocaulon juventas	Cardenolide Compound 9	HT-1080	93	14640513
Streptocaulon juventas	Cardenolide Compound 10	HT-1080	96	14640513
Streptocaulon juventas	Cardenolide Compound 16	HT-1080	160	14640513
Streptocaulon juventas	Cardenolide Compound 18	HT-1080	180	14640513
Streptocaulon juventas	Cardenolide Compound 4	HT-1080	220	14640513
Streptocaulon juventas	Cardenolide Compound 14	HT-1080	460	14640513
Streptocaulon juventas	Cardenolide Compound 3	HT-1080	590	14640513
Streptocaulon juventas	Cardenolide Compound 5	HT-1080	670	14640513
Streptocaulon	Cardenolide Compound	HT-1080	810	14640513

juventas	11			
Streptocaulon juventas	Cardenolide Compound 1	HT-1080	1100	14640513
Streptocaulon juventas	Cardenolide Compound 8	HT-1080	1200	14640513
Streptocaulon juventas	Cardenolide Compound 12	HT-1080	1500	14640513
Streptocaulon juventas	Cardenolide Compound 2	HT-1080	1600	14640513
Aspergillus terreus	Asterriquinone C-1	MCF-7	4100	14695798
Aspergillus terreus	(+)-5(6)-Dihydro-6-Hydroxyterrecyclic Acid A	SF-268	7800	14695798
Aspergillus terreus	Betulinan A	MCF-7	8700	14695798
Aspergillus terreus	(+)-Terrecyclic Acid A Methyl Ester	NCI-H460	9400	14695798
Aspergillus terreus	(+)-Terrecyclic Acid A	NCI-H460	10600	14695798
Aspergillus terreus	(-)-Quadrone	NCI-H460	12400	14695798
Aspergillus terreus	Asterriquinone D	SF-268	15800	14695798
Aspergillus terreus	Asterredione	NCI-H460	17400	14695798
Aspergillus terreus	(+)-5(6)-Dihydro-6-Methoxyterrecyclic Acid A	NCI-H460	19400	14695798

Ludwigia octovalvis	(3Z)-Coumaroylhederagenin	KB	1200	14738395
Ludwigia octovalvis	(23E)-Coumaroylhederagenin	KB	1300	14738395
Ludwigia octovalvis	(23Z)-Coumaroylhederagenin	KB	1600	14738395
Brucea javanica (L.) Merr.	Bruceoside	KB and RPMI-7951 cell lines	250	14987069
Bucida buceras L.	Bucidarasin A	human tumor cellines	500	14987069
Bucida buceras L.	Bucidarasin B	human tumor cellines	1900	14987069
Bucida buceras L.	Bucidarasin C	human tumor cellines	1900	14987069
Mundulea chapelieri	Rotenolone	A2780	1250	15043430
Mundulea chapelieri	Rotenone	A2780	1750	15043430
Mundulea chapelieri	Tephrosin	A2780	22750	15043430
Mundulea chapelieri	Mundulone	A2780	32500	15043430
Mundulea chapelieri	8R-Acetoxyelemol	A2780	42500	15043430
Mundulea chapelieri	Isomundulinol	A2780	45000	15043430
Mundulea chapelieri	Ms-Ii	A2780	50000	15043430

Mundulea chapelieri	Munetone	A2780	50000	15043430
Mundulea chapelieri	8-(3,3-Dimethylallyl)-5,7-Dimethoxyflavanone	A2780	57500	15043430
Mundulea chapelieri	Mundulinol	A2780	62500	15043430
Mundulea chapelieri	3-Deoxy-Ms-Ii	A2780	82500	15043430
Dendrolobium lanceolatum	Flavanone Compound 1	NCI-H187	1500	15217275
Dendrolobium lanceolatum	Flavanone Compound 2	NCI-H187	20250	15217275
Acridocarpus vivy	Acridocarpusic Acid C	A2780	1750	15217279
Acridocarpus vivy	Acridocarpusic Acid D	A2780	14750	15217279
Acridocarpus vivy	Oleanolic Acid	A2780	20000	15217279
Acridocarpus vivy	Ursolic Acid	A2780	23000	15217279
Acridocarpus vivy	Acridocarpusic Acid B	A2780	29000	15217279
Acridocarpus vivy	Moronic Acid	A2780	32750	15217279
Acridocarpus vivy	Acridocarpusic Acid E	A2780	38250	15217279
Acridocarpus vivy	Acridocarpusic Acid A	A2780	39750	15217279
Lantana involucrata	Lantalucratin B	KB-CPT, MCF-7	1000	15217280
Lantana involucrata	Dehydroiso- A -Lapachone	MCF-7	1200	15217280

Lantana involucrata	Dehydroiso- B -Lapachone	MCF-7	1200	15217280
Lantana involucrata	Lantalucratin E	1A9	1300	15217280
Lantana involucrata	Lantalucratin A	MCF-7	1600	15217280
Lantana involucrata	Lantalucratin C	HOS	4700	15217280
Garcinia xanthochymus	Guttiferone E	SW-480	7500	15787435
Garcinia xanthochymus	Xanthochymol	SW-480	8300	15787435
Garcinia xanthochymus	Guttiferone H	SW-480	12400	15787435
Garcinia xanthochymus	Cycloxanthochymol	SW-480	16600	15787435
Garcinia xanthochymus	Aristophenone A	SW-480	33300	15787435
Garcinia xanthochymus	Gambogenone	DPPH	38700	15787435
Garcinia xanthochymus	Fukugetin	DPPH	62000	15787435
Garcinia xanthochymus	Amentoflavone	SW-480	111000	15787435
Garcinia	Fukugiside	DPPH	116000	15787435

xanthochymus				
Garcinia xanthochymus	Alloathyriol	SW-480	117000	15787435
Garcinia xanthochymus	3,8′′-Biapigenin	SW-480	185000	15787435
Garcinia xanthochymus	Volkensiflavone	SW-480	185000	15787435
Schizolaena hystrix	Nymphaeol A	A2780	13750	15787448
Schizolaena hystrix	Schizolaenone A	A2780	25000	15787448
Schizolaena hystrix	Schizolaenone B	A2780	27500	15787448
Schizolaena hystrix	Macarangaflavanone B	A2780	40000	15787448
Schizolaena hystrix	4′-O-Methylbonannione A	A2780	42500	15787448
Thelypteris torresiana	Protoapigenone	MDA-MB-231	675	16206043
Peperomia duclouxii	Bioactive Dibenzylbutyrolactone And Dibenzylbutanediol Lignan Compound 7	VA-13	23200	16499322
Peperomia duclouxii	Bioactive Dibenzylbutyrolactone And Dibenzylbutanediol Lignan Compound 2	HepG2	42800	16499322

Peperomia duclouxii	Bioactive Dibenzylbutyrolactone And Dibenzylbutanediol Lignan Compound 11	VA-13	126900	16499322
Peperomia duclouxii	Bioactive Dibenzylbutyrolactone And Dibenzylbutanediol Lignan Compound 1	VA-13	130300	16499322
Peperomia duclouxii	Bioactive Dibenzylbutyrolactone And Dibenzylbutanediol Lignan Compound 4	VA-13	130400	16499322
Peperomia duclouxii	Bioactive Dibenzylbutyrolactone And Dibenzylbutanediol Lignan Compound 8	WI-38	141400	16499322
Peperomia duclouxii	Bioactive Dibenzylbutyrolactone And Dibenzylbutanediol Lignan Compound 13	HepG2	151400	16499322

Peperomia duclouxii	Bioactive Dibenzylbutyrolactone And Dibenzylbutanediol Lignan Compound 9	VA-13	156600	16499322
Peperomia duclouxii	Bioactive Dibenzylbutyrolactone And Dibenzylbutanediol Lignan Compound 5	VA-13	183300	16499322
Peperomia duclouxii	Bioactive Dibenzylbutyrolactone And Dibenzylbutanediol Lignan Compound 3	HepG2	200500	16499322
Peperomia duclouxii	Bioactive Dibenzylbutyrolactone And Dibenzylbutanediol Lignan Compound 10	VA-13	228600	16499322
Peperomia pellucida	Compound 1	HL-60	1400	16499324
Peperomia pellucida	Peperomin E	HL-60	1800	16499324
Peperomia pellucida	Compound 2	HL-60	10800	16499324
Peperomia pellucida Kunth (Piperaceae)	Secolignan Compound 1	HL-60	1400	16499324
Peperomia pellucida	Peperomin E	HL-60	1800	16499324

Kunth (Piperaceae)				
Peperomia pellucida Kunth (Piperaceae)	Secolignan Compound 2	HL-60	10800	16499324
Goniothalamus marcanii	5-Hydroxy-3-Amino-2-Aceto-1,4-Naphthaquinone	A-549, HT-29, MCF7, RPMI and U251	40	17017852
Croton lechleri	Taspine	KB and V-79 cells	975	17017852
Croton cajucara	Trans-Crotonin	Ehrlich carcinoma	16000	17017852
Croton cajucara	Trans-Dehydrocrotonin	Ehrlich carcinoma	16000	17017852
Albizia gummifera	Gummiferaoside C	A2780	260	17263578
Albizia gummifera	Gummiferaoside A	A2780	370	17263578
Albizia gummifera	Gummiferaoside B	A2780	700	17263578
Macaranga alnifolia	Vedelianin	A2780	130	17326683
Macaranga alnifolia	Schweinfurthin E	A2780	260	17326683
Macaranga alnifolia	Schweinfurthin G	A2780	390	17326683
Macaranga alnifolia	Schweinfurthin H	A2780	4500	17326683
Macaranga alnifolia	Schweinfurthin F	A2780	5000	17326683
Macaranga alnifolia	Diplacone	A2780	10500	17326683
Macaranga alnifolia	Diplacol	A2780	11500	17326683

Macaranga alnifolia	Bonanniol A	A2780	23500	17326683
Macaranga alnifolia	Bonannione A	A2780	24500	17326683
Macaranga alnifolia	Alnifoliol	A2780	27300	17326683
Peperomia duclouxii	Bioactive Lignans Compound 3	VA-13	5300	17358082
Peperomia duclouxii	Bioactive Lignans Compound 6	WI-38	41300	17358082
Peperomia duclouxii	Zhepiresinol	VA-13	50700	17358082
Peperomia duclouxii	Bioactive Lignans Compound 5	WI-38	74300	17358082
Peperomia duclouxii	Medioresinol	WI-38	76900	17358082
Peperomia duclouxii	Bioactive Lignans Compound 2	WI-38	96600	17358082
Peperomia duclouxii	(1S,2R,5S,6R)-2-(5-Methoxy-3,4-Methylenedioxyphenyl)-6-(3,4-Di-Hydroxy-5-Methoxyphenyl)-3,7-Dioxabicyclo[3.3.0]Octane	VA-13	13250	17358082

Peperomia duclouxii	(1R,2R,3S)-1-(4-Hydroxy-3,5-Dimethoxyphenyl)-6,7-Methylene-Dioxy-8-Methoxy-1,2,3,4-Tetrahydronaphthalene-2,3-Dimethanol Di-Acetate	WI-38	103250	17358082
Peperomia duclouxii	(2S,3R,4R)-2-(5-Methoxy-3,4-Methylenedioxyphenyl)-4-(5-Meth-Oxy-3,4-Methylenedioxybenzyl)-3-Furanmethanol	Hep G2	180000	17358082
Peperomia duclouxii	(1S,2R,5S,6R)-2-(5-Methoxy-3,4-Methylenedioxyphenyl)-6-(4-Hydroxy-3,5-Dimethoxyphenyl)-3,7-Dioxabicyclo[3.3.0]Octane	WI-38	241500	17358082
	2-Acetonyl-8-(2-Furoylmethyl)-7-Hydroxy-5-Methyl-Chromone	ehrlich ascites tumor cells	250000	17485848
Elaeodendron sp.	Elaeodendroside B	A2780	19	17547460

Elaeodendron sp.	Elaeodendroside T	A2780	85	17547460
Elaeodendron sp.	Elaeodendroside G	A2780	100	17547460
Elaeodendron sp.	Elaeodendroside F	A2780	190	17547460
Elaeodendron sp.	(2 A ,3 B ,14 B)-Trihydroxy-3-O-(4-Deoxy-3-O-Methyl-R-L-Erythro pentopyranosyl)Card-4, 20(22)-Dienolide	A2780	2500	17547460
Elaeodendron sp.	Elaeodendroside U	A2780	30000	17547460
Nerium oleander	Cardenolide Compound 1	WI-38	20000	17595134
Nerium oleander	Cardenolide Compound 6	WI-38	70000	17595134
Nerium oleander	Cardenolide Compound 11	WI-38	80000	17595134
Nerium oleander	Cardenolide Compound 13	WI-38	90000	17595134
Nerium oleander	Cardenolide Compound 12	VA-13	160000	17595134
Nerium oleander	Cardenolide Compound 7	WI-38	370000	17595134

Nerium oleander	Cardenolide Compound 4	VA-13	720000	17595134
Nerium oleander	Cardenolide Compound 10	VA-13	1600000	17595134
Nerium oleander	Cardenolide Compound 5	VA-13	1900000	17595134
Nerium oleander	Cardenolide Compound 9	VA-13	13400000	17595134
Nerium oleander	Cardenolide Compound 2	WI-38	16300000	17595134
Nerium oleander	Cardenolide Compound 3	WI-38	40900000	17595134
Nerium oleander	Cardenolide Compound 8	WI-38	102000000	17595134
Saussurea stella Maxim.	3-O-β-D-Xylopyranosylstrophanthidin	BGC-823	16	17844995
Saussurea stella Maxim.	Convallatoxin	BGC-823	16	17844995
Saussurea stella Maxim.	3-O-β-D-Quinovopyranosylstrophanthidin	Bel-7402	20	17844995

Saussurea stella Maxim.	3-O- α -L-Rhamnopyranosylcannogenol	Bel-7402	28	17844995
Saussurea stella Maxim.	3-O- α -L-Rhamnopyranosylacovenosigenin α	BGC-823	56	17844995
Saussurea stella Maxim.	3-O- β -D-Quinovopyranosylperiplogenin	BGC-823	70	17844995
Saussurea stella Maxim.	3-O- β -D-Fucopyranosylperiplogenin	BGC-823	120	17844995
Saussurea stella Maxim.	3-O- β -D-Xylopyranosylperiplogenin	BGC-823	230	17844995
Saussurea stella Maxim.	3-O- β -D-Glucopyranosyl-(1F4)-R-L-Rhamnopyranosylcannogenin	BGC-823	290	17844995
	Wedelolactone	prostate cancer AR dependent Pca	800	17942463

		cell 22Rv1		
	Luteolin	prostate cancer AR dependent Pca cell 22Rv1	1720	17942463
	Apigenin	prostate cancer AR dependent Pca cell 22Rv1	3020	17942463
	Indole-3-Carboxylaldehyde	prostate cancer AR dependent Pca cell 22Rv1	656000	17942463
Roupellina (Strophanthus) boivinii	Corotoxigenin 3-O- β -D-Boivinoside	A2780	150	17988099
Roupellina (Strophanthus) boivinii	Digitoxigenin 3-O-[β -D-Glucopyrananosyl-(1 F4)-R-L-Acofriopyra-Noside]	A2780	150	17988099
Roupellina (Strophanthus) boivinii	Uzarigenin 3-O- α -L-Rhamnoside	A2780	150	17988099

Roupellina (Strophanthus) boivinii	Boivinide A	A2780	170	17988099
Roupellina (Strophanthus) boivinii	Boivinide E	A2780	280	17988099
Roupellina (Strophanthus) boivinii	Boivinide D	A2780	290	17988099
Roupellina (Strophanthus) boivinii	Boivinide F	A2780	540	17988099
Roupellina (Strophanthus) boivinii	Boivinide B	A2780	660	17988099
Roupellina (Strophanthus) boivinii	Boivinide C	A2780	2900	17988099
Roupellina (Strophanthus) boivinii	17α-Corotoxigenin 3-O-β-D-Sarmentoside	A2780	3700	17988099
Croton alnifolius	12-O-Tetradecanoylphorbol-13-Acetate	K562 cell	0.3	18163590
Strychnos mitscherlichii	Bisnordihydrotoxiferine	HT-29 and K562 cell lines	2500	18163590
Picramnia sellowii	Nataloe-Emodin	H460 cell line	12000	18163590
Iryanthera juruensis	2′,4′-Dihydroxy-4,6′-Dimethoxydihydrochalcone	K562 cell	18500	18163590

Iryanthera juruensis	2′,4′-Dihydroxy-6′-Methoxy-3,4-Methylene dioxydihydrochalcone	K562 cell	21000	18163590
Malleastrum sp. (Meliaceae)	Malleastrone B	U937	190	18177014
Malleastrum sp. (Meliaceae)	Malleastrone A	U937	200	18177014
Malleastrum sp. (Meliaceae)	Malleastrone C	A2780	18000	18177014
S. miltiorrhiza	Miltirone	CCRF-CEM	600	18202018
S. miltiorrhiza	Tanshinone I	CEM/ADR5000	3100	18202018
S. miltiorrhiza	Tanshinone Iia	CCRF-CEM	5400	18202018
Bridelia ferruginea	B-Peltatin	SF-295	1	18327911
Bridelia ferruginea	Deoxypodophyllotoxin	BXPC-3	1.1	18327911
Dorypleres splendens	Bengamide A	NCI-H460	1.35	18327911
Streptomyces annulatus	Actinomycin D	P388	2.5	18327911
Dolabella auricularia	Majusculamide C	KM20L2	3.25	18327911
Streptomyces sp.	Echinomycin (Quinomycin A)	P388	3.675	18327911
Jaspis spp. ,	Toyocamycin	P388	5.75	18327911

Streptomyces sp.				
Alangium Villosum	Cephaeline	P388	6.75	18327911
Dorypleres splendens	Bengazole A	KM20L2	7.75	18327911
Petrosia sp.	Manzamine A	P388	16.75	18327911
Jaspis spp.	Jaspamide	P388	20	18327911
Bridelia ferruginea	Isopicrodeoxypodophyllotoxin	P388	25	18327911
Stelletta globostellata	Stellettin A	P388	30	18327911
Stelletta globostellata	Stellettin B	P388	92.5	18327911
Dorypleres splendens	Bengazole B	P388	132.5	18327911
Dorypleres splendens	Bengazole E	P388	185	18327911
Penicillium rugulosum	4-Hydroxy-6-Methoxy-Γ ,7-Dimethyl-3-Oxo-Phthalansorbic Acid	P388	275	18327911
Bridelia ferruginea	Neoechinulin A	KM20L2	475	18327911
Bridelia ferruginea	β -Peltatin 5-O- β -D-Glucopyranoside	P388	525	18327911
Stylissa flabelliformis	Axinastatin 5	KM20L2	700	18327911
Agelas sp.	Agelasphin-9B	NCI-H460	1425	18327911
Phoma glomerata	Genistein	NCI-H460	3750	18327911
Pseudaxinella sp.,	Hymenialdisine	P388	6000	18327911

Phakellia dendyi				
Phoma glomerata	Daidzein	SF-268	11000	18327911
Pseudaxinella sp.	Debromohymenialdisine	P388	12500	18327911
Realgar	Tetraarsenic Tetrasulfide	NB4 cells	1100	18344322
	Curcumin	BxPC-3 cells	3100	18640131
	Isoflavone	BxPC-3 cells	183000	18640131
red grapes	Resveratrol	colon cancer cells DLD-1	10000	18678136
cinnamon	Cinnamaldehyde	colon cancer cells DLD-2	100000	18678136
black pepper	Piperine	colon cancer cells DLD-3	100000	18678136
	Senkyunolide A	HT-29	26000	18718517
	Z-Ligustilide	HT-29	28800	18718517
	N-Butylidenephthalide	HT-29	111475	18718517
Cedrus deodara	(-)-Wikstromal	Molt-4 cell	37500	18781909
Schefflera heptaphylla (L.) Frodin	(-)-Beta-Pinene And (+)-Beta-Pinene	MCF-7, A375 and HepG2	147000	18814213
	5-Hydroxy-6-Isobutyryl-7-Methoxy-2,2-Dimethyl-	U-373 MG glioblastoma	48500	19003947

	Benzopyran	cell-line		
	7-Hydroxy-6-Isobutyryl-5-Methoxy-2,2-Dimethylbenzopyran	U-373 MG glioblastoma cell-line	48500	19003947
	6-Isobutyryl-5,7-Dimethoxy-2,2-Dimethyl-Benzopyran	U-373 MG glioblastoma cell-line	69500	19003947
Allium stipitatum	2-(Methyldithio)Pyridine-N-Oxide	A549	220	19093848
Allium stipitatum	2-[(Methylthiomethyl)Dithio]Pyridine-N-Oxide	MCF7	390	19093848
Aglaia foveolata	Silvestrol	cancer cells	1.5	19333864
Pseudomonas	Spiruchostatin A	breast, prostate and lung cancer cells	16	19333864
Synoicum adareanum	Palmerolide A	melanoma cell UACC-62	18	19333864
Pancratium littorale Jacq.	Pancratistatin 3,4-O-Cyclic Phosphate Sodium Salt	P338 murine cancer cell line (ED50 0.01 μ	25	19333864

		g/ml) and		
Symploca	Symplocamide A	neuro-2A neuroblastoma cells	29	19333864
taxonomically uncharacterized sponge of the family Neopeltidae	Neopeltolide	A549 lung adenocarcinoma	100	19333864
Periconia byssoides	Pericosine A	P388 lymphocytic leukemia cell	300	19333864
Alvaradoa haitiensis Urb.	Alvaradoin E	LNCaP	1120	19333864
Periconia byssoides	Pericosine B	P388 lymphocytic leukemia cell	10000	19333864
Aceriphyllum rossii	23-Hydroxy-3-Oxoolean-12-En-27-Oic Acid	LLC	6560	19618898
Aceriphyllum rossii	β-Peltoboykinolic Acid	LLC	7630	19618898

Aceriphyllum rossii	Aceriphyllic Acid A	LLC	8360	19618898
Aceriphyllum rossii	3β-Hydroxyolean-12-En-29-Oic Acid	LLC	16620	19618898
Aceriphyllum rossii	Aceriphyllum Acid I	LLC	39250	19618898
Aceriphyllum rossii	Aceriphyllic Acid C	LLC	47820	19618898
Aceriphyllum rossii	3-Oxoolean-12-En-27-Oic Acid	MCF-7	53570	19618898
Aceriphyllum rossii	Aceriphyllic Acid F	MCF-7	58440	19618898
Aceriphyllum rossii	Aceriphyllic Acid E	MCF-7	107220	19618898
Soymida febrifuga	Stilbene Compound 6	colon 26-L5	2960	19689125
Soymida febrifuga	Chalcone Compound 8	colon 26-L5	4710	19689125
Soymida febrifuga	Dihydrochalcone Compound 22	A549	12000	19689125
Soymida febrifuga	Chalcone Compound 26	colon 26-L5	14100	19689125
Soymida febrifuga	Homoisoflavan Compound 11	HT-1080	17500	19689125
Soymida febrifuga	Flavan Compound 12	HT-1080	24100	19689125
Soymida febrifuga	Chalcone Compound 21	colon 26-L5	28900	19689125
Soymida febrifuga	Homoisoflavanone Compound 9	colon 26-L5	30600	19689125
Soymida febrifuga	Dihydrochalcone	colon 26-L5	32200	19689125

	Compound 10			
Soymida febrifuga	Stilbene Compound 15	colon 26-L5	33300	19689125
Soymida febrifuga	Flavan Compound 2	A549	35500	19689125
Soymida febrifuga	Stilbene Compound 17	colon 26-L5	35800	19689125
Soymida febrifuga	Dihydrochalcone Compound 24	colon 26-L5	40200	19689125
Soymida febrifuga	Coumarin Compound 3	HT-1080	40300	19689125
Soymida febrifuga	Flavan Compound 5	B16-BL6	40800	19689125
Soymida febrifuga	Flavan Compound 14	colon 26-L5	43900	19689125
Soymida febrifuga	Homoisoflavan Compound 13	colon 26-L5	44500	19689125
Soymida febrifuga	Homoisoflavan Compound 1	colon 26-L5	49500	19689125
Soymida febrifuga	Homoisoflavanone Compound 18	colon 26-L5	57800	19689125
Soymida febrifuga	Dihydrochalcone Compound 19	colon 26-L5	68100	19689125
Soymida febrifuga	Homoisoflavanone Compound 20	colon 26-L5	69300	19689125
Soymida febrifuga	Flavan Compound 7	colon 26-L5	71000	19689125
Soymida febrifuga	Homoisoflavanone Compound 16	colon 26-L5	89500	19689125

Soymida febrifuga	Stilbene Compound 25	colon 26-L5	96900	19689125
	Quercetin 3- β -D-Glucoside	MCF-7	46400	19694432
Dodonaea Wiscosa	Dodonaeaside B	A2780	700	19719093
Dodonaea Wiscosa	Dodonaeaside A	A2780	790	19719093
Garcinia mangostana	A-Mangostin	HT-29	1700	19839614
Garcinia mangostana	B-Mangostin	HT-29	1700	19839614
Garcinia mangostana	Garcinone D	HT-29	2300	19839614
Garcinia mangostana	3-Isomangostin	HT-29	4900	19839614
Garcinia mangostana	9-Hydroxycalabaxanthone	HT-29	9100	19839614
Cimicifuga foetida	3′,25-O-Diacetylcimigenol-3-O- β -D-Xylopyranoside	HepG2	710	20121210
Cimicifuga foetida	4′-O-(E)-2-Butenoyl-25-O-Acetylcimigenol-3-O- β -D-Xylopyrano- Side	HepG2	1290	20121210
Cimicifuga foetida	3′-O-Acetyl-23-Epi-26-Deoxyactein	HepG2	1410	20121210
Cimicifuga foetida	Acteinol	HepG2	2560	20121210

Cimicifuga foetida	4′,25-O-Diacetylcimigenol-3-O-β-D-Xylopyranoside	HepG2	2800	20121210
Cimicifuga foetida	Asiaticoside A	HepG2	4020	20121210
Cimicifuga foetida	Actrin-3-One	HepG2	5510	20121210
Cimicifuga foetida	Norcimifugin	HepG2	5550	20121210
Cimicifuga foetida	2′-O-(E)-2-Butenoyl-25-O-Acetylcimigenol-3-O-β-Xylopyanoside	HepG2	6370	20121210
Cimicifuga foetida	25-O-Acetylcimigenol	HepG2	20300	20121210
Cimicifuga foetida	Cimigenol-3-O-α-L-Arabinoside	HepG2	20420	20121210
Cimicifuga foetida	26-Deoxyacteinol	HepG2	27730	20121210
Cimicifuga foetida	3′-O-Acetylactein	HepG2	32080	20121210
Cimicifuga foetida	12-β-Acetoxycimigenol	HepG2	43060	20121210
Cimicifuga foetida	12,25-O-Diacetylcimigenol-3-O-β-D-Xylopyranoside	MCF7	47540	20121210
Alkanna cappadocica	5-O-Methyl-11-O-Acetyl alkannin	MDA-MB-231	90	20405844

Alkanna cappadocica	5-O-Methyl- Β , Β -Dimethylacrylalkannin	MDA-MB-231	260	20405844
Alkanna cappadocica	β , β -Dimethylacrylalkannin	MDA-MB-231	320	20405844
Alkanna cappadocica	11-Deoxyalkannin	SK-BR-3	690	20405844
Alkanna cappadocica	Alkannin	SK-BR-3	780	20405844
Alkanna cappadocica	11-O-Acetylalkannin	AU565	880	20405844
Alkanna cappadocica	5-O-Methyl-11-Deoxyalkannin	AU565	880	20405844
Alkanna cappadocica	8-O-Methyl-11-Deoxyalkannin	MDA-MB-231	5110	20405844
Amaryllidaceae	Narciclasine	the six cancer cell lines under study	50	20415482
Amaryllidaceae	Lycorine	the six cancer cell lines under study	2000	20415482
phytopathogenic and toxigenic fungi	Bislongiquinolide	B16F10	3000	20415482
phytopathogenic and toxigenic fungi	Dihydrotrichodimerol	B16F10	3000	20415482
phytopathogenic and	Scytolide	two cancer	31000	20415482

toxigenic fungi		cell lines		
phytopathogenic and toxigenic fungi	Verrucarin E	two cancer cell lines	44000	20415482
phytopathogenic and toxigenic fungi	Flufuran	two cancer cell lines	73000	20415482
phytopathogenic and toxigenic fungi	Seiricuprolide	two cancer cell lines	100000	20415482
Melodinus tenuicaudatus	Melodinine K	HL-60	100	20462230
Melodinus tenuicaudatus	11-Methoxytabersonine	HL-60	200	20462230
Melodinus tenuicaudatus	Melodinine H	HL-60	1100	20462230
Melodinus tenuicaudatus	Melodinine J	HL-60	3000	20462230
Melodinus tenuicaudatus	Vindolinine	HL-60	6800	20462230
Melodinus tenuicaudatus	O-Methyl-?-Epivin-Canol	HL-60	15900	20462230
Chinese herb named Wu-Chu-Yu	Evodiamine	thyroid cancer cell line ARO	10000	20503248
Pancratium maritimum	Haemanthidine	B16F10	3100	20550100

L.				
S. lutea	Lycorine	A549	4200	20550100
Pancratium maritimum L.	Haemanthamine	A549	4500	20550100
Amaryllis belladonna L.	Amarbellisine	OE21, B16F10	6700	20550100
	Pseudolycorine	A549	7400	20550100
Narcissus tazetta L.	Tazettine	OE21	78000	20550100
Pancratium maritimum L.	Ungeremine	U373	83000	20550100
	Ambelline	OE21	86000	20550100
	Buphanisine	OE21	97000	20550100
	Carnosic Acid	HL60 cells	16800	20661831
Smilax china L.	Compound 2	MCF-7	5250	20669365
Smilax china L.	Compound 3	MCF-7	11250	20669365
Smilax china L.	Compound 5	MCF-7	21000	20669365
Smilax china L.	Compound 1	MCF-7	37750	20669365
Smilax china L.	Compound 6	MCF-7	45000	20669365
Smilax china L.	Compound 4	MCF-7	75500	20669365
Euscaphis japonica	Euscaphic Acid C	NCI-H460	2540	20873721
Euscaphis japonica	2A-Hydroxyursolic Acid	CEM	3270	20873721

Euscaphis japonica	Euscaphic Acid D	MCF-7	3610	20873721
Melia toosendan	Triterpenoid 14	MCF-7	1025	20961091
Melia toosendan	Triterpenoid 2	MCF-7	2750	20961091
Melia toosendan	Triterpenoid 7	MCF-7	5750	20961091
Melia toosendan	Triterpenoid 3	MCF-7	6000	20961091
Melia toosendan	Triterpenoid 6	MCF-7	7500	20961091
Melia toosendan	Triterpenoid 9	U20S	9750	20961091
Melia toosendan	Triterpenoid 8	MCF-7	16000	20961091
Melia toosendan	Triterpenoid 13	U20S	17250	20961091
Melia toosendan	Steroid 20	U20S	23500	20961091
Melia toosendan	Triterpenoid 1	U20S	23750	20961091
Melia toosendan	Triterpenoid 15	U20S	23750	20961091
Melia toosendan	Triterpenoid 4	U20S	23750	20961091
Melia toosendan	Triterpenoid 5	MCF-7	41000	20961091
Melia toosendan	Steroid 11	U20S	58000	20961091
Melia toosendan	Triterpenoid 10	MCF-7	70250	20961091
Melia toosendan	Triterpenoid 12	U20S	77750	20961091
Streptocarpus dunnii	(3R)-Dunnione	BT474	1000	21174407
Streptocarpus dunnii	(3R)-A-Dunnione	BT474	4500	21174407
Streptocarpus dunnii	(3R)-7-Hydroxy-A-Dunnione	BT474	4900	21174407
Poria cocos	Triterpene Acid	A549	4000	21250700

	Compound 5A			
Poria cocos	Triterpene Acid Compound 6A	PANC-1	5500	21250700
Poria cocos	Triterpene Acid Compound 2A	DU145	14500	21250700
Poria cocos	Triterpene Acid Compound 4A	HL60	28400	21250700
Poria cocos	Triterpene Acid Compound 3A	HL60	32600	21250700
Poria cocos	Triterpene Acid Compound 1A	HL60	38000	21250700
Phomopsis archeri	Phomoarcherin B	KKU-M139	250	21341709
Phomopsis archeri	Phomoarcherin C	KKU-100, KKU-M139	22250	21341709
Phomopsis archeri	Phomoarcherin A	KKU-M156	41500	21341709
Phomopsis archeri	Kampanol A	KKU-M213	49000	21341709
Azadiracta indica A. Juss.	28-Deoxonimbolide	SK-BR-3	1700	21381696
Azadiracta indica A. Juss.	7-Deacetyl-7-Benzoylgedunin	HL60	2900	21381696
Azadiracta indica A. Juss.	7-Deacetyl-7-Benzoylepoxyazadiradione	HL60	3100	21381696

Azadiracta indica A. Juss.	7-Benzoylnimbocinol	CRL1579	4000	21381696
Azadiracta indica A. Juss.	Gedunin	HL60	5900	21381696
Azadiracta indica A. Juss.	Epoxyazadiradione	HL60	9300	21381696
Azadiracta indica A. Juss.	Ohchininacetate	HL60	9900	21381696
Azadiracta indica A. Juss.	Azadiradionolide	SK-BR-3	11500	21381696
Azadiracta indica A. Juss.	6-Deacetylnimbin	HL60	11600	21381696
Azadiracta indica A. Juss.	7-Deacetylgedunin	SK-BR-3	12000	21381696
Azadiracta indica A. Juss.	17-Hydroxyazadiradione	HL60	12400	21381696
Azadiracta indica A. Juss.	17-Epiazadiradione	HL60	12600	21381696
Azadiracta indica A. Juss.	Azadiradione	HL60	14700	21381696
Azadiracta indica A. Juss.	1,3-Diacetylvilasinin	AZ521	14800	21381696

Azadiracta indica A. Juss.	Nimbin	CRL1579	15900	21381696
Azadiracta indica A. Juss.	20,30-Dihydrosalannin	HL60	17100	21381696
Azadiracta indica A. Juss.	17-Epi-17-Hydroxyazadiradione	CRL1579	19100	21381696
Azadiracta indica A. Juss.	Desfuranoazadiradione	HL60	19800	21381696
Chaetomium globosum	Globosumone A	NCI-H460	6500	21455524
Chaetomium globosum	Globosumone B	WI-38	14200	21455524
Chaetomium globosum	Chaetopyranin	HMEC	38500	21455524
Penicillium sp.	Penicillenone	P388	1380	21455524
Penicillium sp.	Leptosphaerone C	A549	1450	21455524
Emericella nidulans	Emindole Da	36 human tumor	13750	21455524
Emericella nidulans	Arugosin A	7 out of 36	25000	21455524
Chaetomium globosum	Chaetoglobosin U	KB cell line	16000	21455524
Chaetomium	Chaetoglobosin C	KB cell line	34000	21455524

globosum				
Chaetomium globosum	Penochalasin A	KB cell line	40000	21455524
Chaetomium globosum	Chaetoglobosin E	KB cell line	48000	21455524
Chaetomium globosum	Chaetoglobosin F	KB cell line	52000	21455524
Aspergillus fumigatus	9-Deacetoxyfumigaclavine	K562	3100	21455524
Rhinocladiella sp.	Cytochalasin E	A2780S	37.5	21455524
Rhinocladiella sp.	Cytochalasin 1	A2780S, SW-620	9775	21455524
Rhinocladiella sp.	Cytochalasin 3	A2780S	9775	21455524
Rhinocladiella sp.	Cytochalasin 2	A2780S, SW-620	39000	21455524
Chaetomium globosum	Cytoglobosin C	A549	2260	21455524
Chaetomium globosum	Cytoglobosin D	A549	2550	21455524
Chaetomium sp. IFB-E015	Chaetominine	K562	21000000	21455524
Hypoxylon truncatum	Daldinone D	SW1116	41000	21455524

Hypoxylon truncatum	Daldinone C	SW1116	49500	21455524
Pestalotiopsis sp.	Pestalotiopsone F	L5178Y	22325	21455524
Apiospora montagnei	Epiepoxydon 27	HM02	1750	21455524
Pestalotiopsis fici	Pestalofíciol L	HeLa	8700	21455524
Pestalotiopsis fici	Pestalofíciol J	HeLa	21200	21455524
Pestalotiopsis fici	Pestalofíciol K	HeLa	99300	21455524
Pestalotiopsis fici	Pestalofíciol I	MCF7	136100	21455524
BCC 8616	Depsidone 1	BC	10250	21455524
Fusarium oxysporum	Bikaverin	MIA Pa Ca-2	260	21455524
Fusarium oxysporum	Beauvericin	NCI-H460	1410	21455524
PM0651480	Ergoflavin	ACHN	1200	21455524
Phomopsis longicolla	Dicerandrol B	A549	4500	21455524
Phomopsis longicolla	Dicerandrol C	A549	4500	21455524
Phomopsis longicolla	Dicerandrol A	A549, HCT-116	17500	21455524
ZSU44	Secalonic Acid D	HL-60	380	21455524
Aspergillus clavatus & Paecilomyces sp.	Brefeldin A	Spc-A-1	2.5	21455524
KLAR 5	8-Deoxy-Trichothecin	BC-1	880	21455524
KLAR 5	7A-Hydroxytrichodermol	NCI-H187	1730	21455524
KLAR 5	7A-Hydroxyscirpene	KB, BC-1	8470	21455524

KLAR 5	Trichothecolone	BC-1	10060	21455524
Chaetomium chiversii	Radicicol	MCF-7	30	21455524
Pestalotiopsis photiniae	Photinides A–F	MDA-MB-231	25000	21455524
Eutypella sp.	Ent-4(15)-Eudesmen-11-Ol-1-One	NCI-H187	11000	21455524
Eutypella sp.	Eutypellin A	NCI-H187	12000	21455524
Acremonium sp.	Leucinostatin A	BT-20	2	21455524
Phomopsis sp.	Oblongolide Z	BC	26000	21455524
Phomopsis sp.	Oblongolide Y	BC	48000	21455524
Alternaria sp.	Alternariol	L5178Y	4250	21455524
Alternaria sp.	Alternariol 5-O-Sulfate	L5178Y	11250	21455524
Alternaria sp.	Desmethylaltenusin	L5178Y	15500	21455524
Alternaria sp.	Altenusin	L5178Y	17000	21455524
Alternaria sp.	Alternariol 5-O-Methyl Ether	L5178Y	19500	21455524
Phomopsis sp	2-(7'-Hydroxyoxooctyl)-3-Hydroxy-5-Methoxybenzene Acetic Acid Ethyl Ester	HEp-2	62500	21455524
Aspergillus parasiticus	Sequoiatones A	BC	4000	21455524

Aspergillus parasiticus	Sequoiatones B	BC	4000	21455524
Talaromyces sp.	Kasanosin A	DNA pol b	27300	21455524
Talaromyces sp.	Kasanosin B	DNA pol b	60100	21455524
Aspergillus niger	Rubrofusarin B	SW1116	11250	21455524
Pestalotiopsis microspora	Torreyanic Acid	NEC	8750	21455524
Stemphylium globuliferum	Mixture Of Alterporriol G & Alterporriol H	L5178Y	6750	21455524
Stemphylium globuliferum	6-O-Methylalaternin	L5178Y	10500	21455524
Chaetomium sp.	Cochliodinol	L5178Y	17500	21455524
Chaetomium sp.	Isocochliodinol	L5178Y	178750	21455524
Xylaria sp.	2-Chloro-5-Methoxy-3-Methylcyclohexa-2,5-Diene-1,4- Dione	Vero cells	1350	21455524
Halorosellinia sp. & Guignardia sp.	Anthracenedione 6	KB	3170	21455524
Halorosellinia sp. & Guignardia sp.	Anthracenedione 9	KBv200	34640	21455524
Halorosellinia sp. & Guignardia sp.	Anthracenedione 7	KB	56560	21455524
Halorosellinia sp. &	Anthracenedione 1	KB	57320	21455524

Guignardia sp.				
Halorosellinia sp. & Guignardia sp.	Anthracenedione 14	KB	68390	21455524
Halorosellinia sp. & Guignardia sp.	Anthracenedione 5	KBv200	86450	21455524
Mycelia sterilia	Spiromamakone A	P388	330	21455524
Preussia sp.	Spiropreussione A	A2780	2400	21455524
Periconia atropurpurea	Periconicin B	HeLa and CHO	8000	21455524
Pestalotiopsis terminaliae	Paclitaxel	BT220, H116, INT-407, HL251, HLK210	5	21455524
Phyllosticta spinarum	Tauranin	MCF-7	1500	21455524
XG8D (a basidiomycete)	Merulin C	BT474	3925	21455524
XG8D (a basidiomycete)	Merulin A	SW620	12100	21455524
Xylaria sp.	Eremophilanolides 1 – 3 96–98A	KB, MCF-7, NCI-H187	3800	21455524
Phomopsis sp.	Phomoxanthone A 99A	BC-1	1275	21455524
Phomopsis sp.	Phomoxanthone B 100A	BC-1	1750	21455524

Talaromyces flavus	Talaperoxide D	PC-3	1750	21545109
Talaromyces flavus	Talaperoxide B	PC-3	2225	21545109
Talaromyces flavus	Steperoxide B	PC-3	4550	21545109
Talaromyces flavus	Talaperoxide C	MDA-MB-435	6600	21545109
Talaromyces flavus	Talaperoxide A	PC-3	14250	21545109
	Dibenzoylmethane	SK-N-AS neuroblastoma cell	15900	21704149
	Andrographolide	SK-N-AS neuroblastoma cell	26200	21704149
	Tanshinone IIa	SK-N-AS neuroblastoma cell	53500	21704149
Lysimachia clethroides	Compound 11	HepG2	750	21928797
Lysimachia clethroides	Compound 12	HepG2	990	21928797
Lysimachia clethroides	Compound 13	HepG2	1000	21928797
Lysimachia clethroides	Compound 6	HepG2	1260	21928797
Lysimachia clethroides	Compound 3	HepG2	1480	21928797
Lysimachia clethroides	Compound 4	HepG2	1730	21928797
Lysimachia clethroides	Compound 5	HepG2	6260	21928797
Acorus gramineus	Ligraminol A	SK-MEL-2	4530	21936523

Acorus gramineus	Ligraminol D	A549	8280	21936523
Acorus gramineus	Ligraminol C	A549	9540	21936523
Klyxum simplex	Klysimplexin B	Ca9-22	3900	21970540
	Hirsutalin E	Hep G2	4700	21970540
Klyxum simplex	Klysimplexin H	A549	5100	21970540
Cladiella australis	Australin B	Hep G2	5400	21970540
Klyxum simplex	Simplexin E	MCF-7	11000	21970540
Klyxum simplex	Simplexin A	Daoy	23000	21970540
Klyxum simplex	Simplexin D	MCF-7	23000	21970540
	Hirsutalin A	A549	28000	21970540
Athroisma proteiforme	Athrolide D	A2780	380	21995542
Athroisma proteiforme	Athrolide C	A2780	570	21995542
Athroisma proteiforme	Athrolide E	A2780	1900	21995542
Athroisma proteiforme	Athrolide A	A2780	2100	21995542
Athroisma proteiforme	Athrolide B	A2780	2500	21995542
Uvaria sp.	Uvaricin A	A2780	6400	22136523
Uvaria sp.	Uvaricin B	A2058	7200	22136523
Micromonospora sp.	10 β -Carbomethoxy-7,8,9,10-Tetrahydro-4,6,7 α ,9 α ,11-Pentahydroxy-9-Propyltetra-	HCT-8	6200	22250891

	Cene-5,12-Dione			
Micromonospora sp.	4,6,11-Trihydroxy-9-Propyltetracene-5,12-Dione	HCT-8	12700	22250891
