# Supplementary material for: What Does It Take to Synergistically Combine Sub-Potent Natural Products into Drug-Level Potent Combinations?
Source: PLoS One. 2012 Nov 28;7(11):e49969. doi: 10.1371/journal.pone.0049969 (PMC3509152; doi:10.1371/journal.pone.0049969)
Supplement: Table S4 — Cell-based microbial inhibitory activity values of 609 antimicrobial natural products. (PDF) [file pone.0049969.s004.pdf]

Supplementary Table S4 Cell-based microbial inhibitory activity values of 609 antimicrobial natural products

**Derived From Species**	**Natural Product**	**Against Microbial Species**	**MIC (μg/mL)**	**Reference (Pubmed ID PMID)**
Helichrysum Pedunculatum	Oleic Acid	S. aureus and M. kristinae	1000	10925024
Melia Azedarach L.	4-Hydroxy-3-Methoxycinnamaldehyde		400	12696928
Melia Azedarach L.	(+/-)-Pinoresinol		1000	12696928
Artemisia Annua L. (Asteraceae)	Chrysosplenol-D		250	12842327
Artemisia Annua L. (Asteraceae)	Chrysoplenetin		250	12842327
	Gamma-Linolenic Acid	B. megaterium	50	12952418
	Alpha-Linolenic Acid	B. megaterium	100	12952418
	Genistein	S. aureus	100	12952418
	Pinoresinol	F. verticillioides	1000	15826040
	Scopoletin	F. verticillioides	1500	15826040
	Dehydrocostuslactone	M. tuberculosis H37Rv	6.25	17218447
	Costunolide	M. tuberculosis H37Rv	12.5	17218447
	Salicylaldehyde	Fungus Aspergillus niger	141	17590530
	Linalool	Fungus Aspergillus niger	281	17590530
	Eriodictyol	Salmonella enterica	800	18045389
	Hesperetin	Salmonella enterica	1000	18045389
	Naringenin	Salmonella enterica	1000	18045389
	Carvacrol	Klebsiella pneumoniae ssp. Pneumoniae	50	19347798
	Thymol	Klebsiella pneumoniae ssp.	80	19347798

		Pneumoniae		
Streptomyces Strain	Celastramycin A	M. smegmatis, M. aurum, M. vaccae and M. fortuitum	0.05	14700198
Ecteinascidia Thurstoni	Ecteinascidin 770	M. tuberculosis H37Rv	0.1	14700198
Nocardia Sp.	Tubelactomicin A	M. smegmatis, M. vaccae	0.1	14700198
Didemnum Sp.	Ascididemin	M. aurum A	0.25	14700198
Sargassum Ringgoldianum	Saringosterol	M. tuberculosis H37Rv	0.25	14700198
Nocardia Brasiliensis	Brasiliquinone A	M. smegmatis	0.39	14700198
Salvia Multicaulis	New Norditerpenoid And Diterpenoid 212	M. tuberculosis H37Rv	0.46	14700198
An Endophytic Fungus Of The Genus Phomopsis	Phomoxanthone A	M. tuberculosis H37Rv	0.5	14700198
	Berberine	M. intracellulare	0.78	14700198
Nocardia Brasiliensis	Brasiliquinone B	M. smegmatis	0.78	14700198
Salvia Multicaulis	New Norditerpenoid And Diterpenoid 216	M. tuberculosis H37Rv	0.89	14700198
Marine Sponges	Compound 191	M. tuberculosis H37Rv	0.91	14700198
Ajuga Remota	Ergosterol-5,8-Endoperoxide	M. tuberculosis H37Rv	1	14700198
Strobilanthes Cusia	Tryptanthrin	M. tuberculosis	1	14700198
Salvia Multicaulis	New Norditerpenoid And Diterpenoid 214	M. tuberculosis H37Rv	1.2	14700198
Salvia Multicaulis	New Norditerpenoid And Diterpenoid 215	M. tuberculosis H37Rv	1.2	14700198
Ferula Communis	Ferulenol	M. intracellulare, M. xenopi, M. chelonei and M. smegmatis	1.25	14700198
Pseudomonas Syringae Pv. Syringae	Syringomycin E	M. smegmatis	1.5	14700198
Marine Sponges	Compound 190	M. tuberculosis H37Rv	1.53	14700198
Hypericum Drummondii	Drummondin Family Of Antibiotics 72	M. smegmatis	1.56	14700198

Hypericum Drummondii	Drummondin Family Of Antibiotics 73	M. smegmatis	1.56	14700198
Hypericum Drummondii	Drummondin Family Of Antibiotics 75	M. smegmatis	1.56	14700198
Hypericum Drummondii	Drummondin Family Of Antibiotics 76	M. smegmatis	1.56	14700198
Hypericum Drummondii	Drummondin Family Of Antibiotics 78	M. smegmatis	1.56	14700198
Nocardia Pseudobrasiliensis	Nocardicyclin A	M. smegmatis	1.56	14700198
A Species Of Nocardia	Nothramicin	M. smegmatis	1.56	14700198
Microsphaeropsis Sp.	Preussomerin And Related Compound 113	M. tuberculosis H37Rv	1.56	14700198
Ecteinascidia Thurstoni	Ecteinascidin 786	M. tuberculosis H37Rv	1.6	14700198
Marine Sponges	Compound 196	M. tuberculosis H37Rv	1.77	14700198
Xestospongia Exigua	(+)-Araguspongine C	M. tuberculosis H37Rv	1.9	14700198
Marine Sponges	Compound 198	M. tuberculosis H37Rv	1.93	14700198
Leucas Volkensii	(E)-Phytol	Mycobacterial species	2	14700198
Axinella Cannabina	Axisonitrile-3	M. tuberculosis	2	14700198
Salvia Multicaulis	New Norditerpenoid And Diterpenoid 213	M. tuberculosis H37Rv	2	14700198
Streptomyces Alboniger	Pamamycin-607	M. tuberculosis	2	14700198
	Sesquiterpene Dehydrocostus Lactone 238	M. tuberculosis H37Rv	2	14700198
Marine Sponges	Compound 193	M. tuberculosis H37Rv	2.56	14700198
An Unidentified Species Of Streptomyces	Celastramycin B	M. vaccae	3.1	14700198
Streptomyces Lavendofoliae	Depsidomycin	M. vaccae	3.12	14700198
Hypericum Drummondii	Drummondin Family Of Antibiotics 77	M. smegmatis	3.12	14700198
Microsphaeropsis Sp.	Preussomerin And Related Compound 108	M. tuberculosis H37Rv	3.12	14700198
Microsphaeropsis Sp.	Preussomerin And Related Compound 109	M. tuberculosis H37Rv	3.12	14700198
Philippine Sponge	Agelasine F	M. tuberculosis	3.13	14700198

Agelas Species		H37Rv		
Marine Sponges	Compound 194	M. tuberculosis H37Rv	3.13	14700198
Nocardia Pseudobrasiliensis	Nocardicyclin B	M. smegmatis	3.13	14700198
Marine Sponges	Compound 192	M. tuberculosis H37Rv	3.76	14700198
Melia Volkensii	6β-Hydroxykulactone	M. tuberculosis H37Rv	4	14700198
	Fusidic Acid	M. tuberculosis	4	14700198
J. Excelsa	Ferruginol	M. smegmatis, M. intracellulare, M. xenopi, and M. chelonei	5	14700198
Marine-Derived Pseudomonas Species	Massetolide A	M. tuberculosis	5	14700198
Salvia Multicaulis	New Norditerpenoid And Diterpenoid 211	M. tuberculosis H37Rv	5.6	14700198
Hirsutella Kobayasii	Hirsutellide A	M. tuberculosis H37Rv	6	14700198
	Sclareol	M. tuberculosis H37Rv	6	14700198
Galipea Officinalis	Alkaloid 153	M. tuberculosis	6.25	14700198
Mitracarpus Scaber	Azaanthraquinone 159	M. intracellulare	6.25	14700198
Hypericum Drummondii	Drummondin Family Of Antibiotics 74	M. smegmatis	6.25	14700198
Mitrephora Celebica	Ent-Trachyloban-19-Oic Acid	M. smegmatis	6.25	14700198
Heteronema Erecta	Heteronemin	M. tuberculosis H37Rv	6.25	14700198
Actinomycete Nocardia Brasiliensis	Phenol 63	M. smegmatis	6.25	14700198
Actinomycete Nocardia Brasiliensis	Phenol 64	M. smegmatis	6.25	14700198
An Endophytic Fungus Of The Genus Phomopsis	Phomoxanthone B	M. tuberculosis H37Rv	6.25	14700198
Microsphaeropsis Sp.	Preussomerin And Related Compound 110	M. tuberculosis H37Rv	6.25	14700198
Salvia Multicaulis	New Norditerpenoid And Diterpenoid 217	M. tuberculosis H37Rv	7.3	14700198
Borrichia Frutescens	Cycloartane Triterpenoid 300	M. tuberculosis H37Rv	8	14700198

Borrichia Frutescens	Cycloartane Triterpenoid 301	M. tuberculosis H37Rv	8	14700198
L. Obtusa	Deschloroelatol	M. avium	8	14700198
Marine Species	Diterpene Isonitriles And Isothiocyanates 231	M. tuberculosis H37Rv	8	14700198
Marine Species	Diterpene Isonitriles And Isothiocyanates 232	M. tuberculosis H37Rv	8	14700198
Marine Species	Diterpene Isonitriles And Isothiocyanates 233	M. tuberculosis H37Rv	8	14700198
Marine Species	Diterpene Isonitriles And Isothiocyanates 234	M. tuberculosis H37Rv	8	14700198
Marine Species	Diterpene Isonitriles And Isothiocyanates 235	M. tuberculosis H37Rv	8	14700198
Marine Species	Diterpene Isonitriles And Isothiocyanates 236	M. tuberculosis H37Rv	8	14700198
Aphanizomenon Flos-Aquae	Mueggelone	M. tuberculosis H37Rv	8	14700198
Sarmienta Scandens	Zeorin	M. tuberculosis H37Rv	8	14700198
Aframomum Melegueta	6-Paradol	M. chelonei, M. smegmatis, M. intracellulare and M. xenopi	10	14700198
Colubrina Retusa	Jujubogenin Saponin 342	M. intracellulare	10	14700198
Streptomyces Nrrl 30562	Munumbicin B	M. tuberculosis	10	14700198
Solanum Sodomaeum	Solsodomine A	M. intracellulare	10	14700198
Mitrephora Celebica	13,14-Dihydrooropheic Acid	M. smegmatis	12.5	14700198
Aschersonia Tubulata	15α,22-Dihydroxyhopane Dustanin	M. tuberculosis H37Rv	12.5	14700198
Aschersonia Tubulata	3β-Acetoxy-15α,22-Dihydroxyhopane	M. tuberculosis H37Rv	12.5	14700198
Paecilomyces Tenuipes Bcc 1614	Beauvericin	M. tuberculosis H37Rv	12.5	14700198
Nocardia Brasiliensis	Brasiliquinone C	M. smegmatis	12.5	14700198
Nocardia Brasiliensis	Brasiliquinone D	M. smegmatis	12.5	14700198
	Chelerythrine	M. tuberculosis H37Rv	12.5	14700198
	Chelirubine	M. tuberculosis H37Rv	12.5	14700198
Mitracarpus Scaber	Cleistopholine	M. intracellulare	12.5	14700198
Marine Sponges	Compound 195	M. tuberculosis H37Rv	12.5	14700198

Pseudopterogorgia Elisabethae	Elisapterosin B	M. tuberculosis H37Rv	12.5	14700198
	Macarpine	M. tuberculosis H37Rv	12.5	14700198
Sorangium Cellulosum	Maracen	M. tuberculosis H37Rv	12.5	14700198
Sorangium Cellulosum	Maracin	M. tuberculosis H37Rv	12.5	14700198
Erigeron Philadelphicus	Matricaria Lactone 32	M. tuberculosis H37Rv	12.5	14700198
	Nitidine	M. tuberculosis H37Rv	12.5	14700198
Mitrephora Celebica	Oropheic Acid	M. smegmatis	12.5	14700198
Microsphaeropsis Sp.	Preussomerin And Related Compound 111	M. tuberculosis H37Rv	12.5	14700198
Pseudopterogorgia Elisabethae	Pseudopteroxazole	M. tuberculosis H37Rv	12.5	14700198
Chondrosia Chucalla	Puupehenone	M. tuberculosis H37Rv	12.5	14700198
	Sanguinarine	M. tuberculosis H37Rv	12.5	14700198
Pseudopterogorgia Elisabethae	Serrulatane Diterpene 226	M. tuberculosis H37Rv	12.5	14700198
	Juniperexcelsic Acid	M. tuberculosis H37Rv	14.4	14700198
Aframomum Melegueta	6-Shogaol	M. chelonei, M. smegmatis, M. intracellulare and M. xenopi	15	14700198
J. Excelsa	Sandaracopimaric Acid	M. tuberculosis	15	14700198
Psoralea Corylifolia	Bakuchiol	M. aurum	15.8	14700198
Chamaecyparis Nootkatensis	(+)-Totarol	M. tuberculosis H37Rv	16	14700198
Melia Volkensii	12β-Hydroxy	M. tuberculosis H37Rv	16	14700198
	6-Epi-Deacetyllaurenobiolide	M. tuberculosis H37Rv	16	14700198
	Allolaurinterol	M. tuberculosis	16	14700198
	Aromaticin	Mycobacterial species	16	14700198
Montanoa Speciosa	Encelin	M. tuberculosis H37Rv	16	14700198
Junella Tridens	Epioleanolic Acid 327	M. tuberculosis	16	14700198

		H37Rv		
Melia Volkensii	Methyl Ester 307	M. tuberculosis H37Rv	16	14700198
Junella Tridens	Oleanonic Compound 326	M. tuberculosis H37Rv	16	14700198
	Parthenolide	M. tuberculosis H37Rv	16	14700198
Lippia Turbinata	Rehmannic Acid	M. tuberculosis H37Rv	18	14700198
Erythrina Indica	Indicanine B	M. smegmatis	18.5	14700198
Azorella Madreporica	A New Mulinane Skeleton Diterpene 209	M. tuberculosis H37Rv	20	14700198
Zingiber Officinale	10-Gingerol	M. tuberculosis H37Rv	25	14700198
Zingiber Officinale	8-Gingerol	M. tuberculosis H37Rv	25	14700198
Paecilomyces Tenuipes Bcc 1614	Beauvericin A	M. tuberculosis H37Rv	25	14700198
Solidago Canadensis	C-10-O-Acylated Derivative 29	M. tuberculosis H37Rv	25	14700198
Tetradenia Riparia	Diterpenediol	M. tuberculosis	25	14700198
Serpula Himantoides	Himanimides C	M. phlei	25	14700198
Elysia Rufescens	Kahalalide F	M. intracellulare	25	14700198
Glycyrrhiza Glabra	Licoisoflavanone	M. tuberculosis	25	14700198
Microsphaeropsis Sp.	Preussomerin And Related Compound 107	M. tuberculosis H37Rv	25	14700198
Microsphaeropsis Sp.	Preussomerin And Related Compound 112	M. tuberculosis H37Rv	25	14700198
Amyris Elemifera	Texalin	M. tuberculosis, M. avium and M. kansasii	25	14700198
Solidago Canadensis	Triyne	M. tuberculosis H37Rv	25	14700198
Vatica Oblongifolia Ssp. Oblongifolia	Vaticaphenol A	M. smegmatis	25	14700198
Sarmienta Scandens	Lupeol	M. tuberculosis H37Rv	27	14700198
Baccharis Patagonica	Erythrodiol	M. tuberculosis H37Rv	28	14700198
Monttea Aphylla	Epi-Betulinic Acid	M. tuberculosis H37Rv	29	14700198
Marine Sponges	Compound 197	M. tuberculosis H37Rv	30.2	14700198

Cryptolepis Sanguinolenta	Neocryptolepine	M. fortuitum	31	14700198
I. Helenium	Alantolactone	M. tuberculosis H37Rv	32	14700198
Montanoa Speciosa	Compound 260	M. tuberculosis H37Rv	32	14700198
	Damsin	Mycobacterial species	32	14700198
L. Elata	Elatol	M. tuberculosis H37Rv	32	14700198
Plocamium Cartilagineum	Halogenated Monoterpene 199	M. tuberculosis H37Rv	32	14700198
I. Helenium	Isoalantolactone	M. tuberculosis H37Rv	32	14700198
Chrysanthemum Cinerariaefolium	Pyrethrin Ii	M. tuberculosis H37Rv	32	14700198
Lippia Turbinata	Camaric Acid	M. tuberculosis H37Rv	36	14700198
Lippia Turbinata	Compound 330	M. tuberculosis H37Rv	38	14700198
Agrocybe Sp.	Agrocybolactone	M. smegmatis	50	14700198
Galipea Officinalis	Alkaloid 148	M. tuberculosis	50	14700198
Ferula Communis	Ferchromone	M. intracellulare, M. xenopi, M. chelonei and M. smegmatis	50	14700198
Potamogeton Malaianus	Furanoid Labdane Diterpene 223	M. tuberculosis H37Rv	50	14700198
Vatica Oblongifolia Ssp. Oblongifolia	Hopeaphenol A	M. smegmatis	50	14700198
Tabernaemontana Citrifolia	Ibogaine	M. tuberculosis, M. avium and M. kansasii	50	14700198
Colubrina Retusa	Jujubogenin Saponin 341	M. intracellulare	50	14700198
Chrysoma Pauciflosculosa	Matricaria Ester 26	M. tuberculosis H37Rv	50	14700198
Chrysoma Pauciflosculosa	Matricaria Ester 27	M. tuberculosis H37Rv	50	14700198
Neuropogon Sp.	Neuropogonine A	M. vaccae	50	14700198
Neuropogon Sp.	Neuropogonine B	M. vaccae	50	14700198
Neuropogon Sp.	Neuropogonine C	M. vaccae	50	14700198
Microsphaeropsis Sp.	Preussomerin And Related Compound	M. tuberculosis	50	14700198

	114	H37Rv		
	1,10-Epoxycostunolide	M. tuberculosis H37Rv	64	14700198
A Chilean Propolis	14-Acetylviscidone	M. tuberculosis	64	14700198
Fischerella Ambigua	Ambigol A	M. tuberculosis H37Rv	64	14700198
A Chilean Propolis	Coniferyl Aldehyde	M. tuberculosis	64	14700198
	Debromolaurinterol	M. tuberculosis	64	14700198
A Chilean Propolis	Dihydrobenzofuran Derivative	M. tuberculosis	64	14700198
Sarcophyton Sp.	Isosarcophytoxide	M. tuberculosis H37Rv	64	14700198
Iva Imbricata	Ivalin	M. tuberculosis H37Rv	64	14700198
Buddleja Cordata Subsp. Cordata	Long Chain Fatty Acid Ester 66 Of 2-(4 -Hydroxyphenyl)Ethanol	M. tuberculosis H37Rv	64	14700198
	Parthenin	Mycobacterial species	64	14700198
Chrysanthemum Cinerariaefolium	Pyrethrin I	M. tuberculosis H37Rv	64	14700198
	Reynosin	M. tuberculosis H37Rv	64	14700198
	Santamarine	M. tuberculosis H37Rv	64	14700198
Junella Tridens	Triterpene 328	M. tuberculosis H37Rv	64	14700198
Junella Tridens	Triterpene 329	M. tuberculosis H37Rv	64	14700198
Adhatoda Vasica	Vasicine	M. tuberculosis	64	14700198
Letharia Columbiana	Vulpinic Acid	M. tuberculosis H37Rv	64	14700198
Potamogeton Malaianus	Furanoid Labdane Diterpene 220	M. tuberculosis H37Rv	100	14700198
Potamogeton Malaianus	Furanoid Labdane Diterpene 221	M. tuberculosis H37Rv	100	14700198
Potamogeton Malaianus	Furanoid Labdane Diterpene 222	M. tuberculosis H37Rv	100	14700198
Tabernaemontana Citrifolia	Voacangine	M. tuberculosis, M. avium and M. kansasii	100	14700198
Engelhardia Roxburghiana	3-Methoxyjuglone	M. tuberculosis H37Rv	0.2	17389998
	Engelhardione	M. tuberculosis	0.2	17389998

		H37Rv		
Phomopsis Sp. Strain Usia5	3-Nitropropionic Acid	M. tuberculosis H37Ra	0.4	17389998
Acanthostrongylophora Sp.	6-Hydroxymanzamine E	M. tuberculosis H37Rv	0.4	17389998
Acanthostrongylophora Sp.	8-Hydroxymanzamine J	M. intracellulare	0.45	17389998
Ferula Communis	Ferulenol	M. smegmatis	0.5	17389998
Peucedanum Ostruthium	Ostruthin	M. abscessus, M. aurum, M. fortuitum, M. phlei and M. smegmatis	1	17389998
Pimpinella Sp.	Phenylpropanoid 30	M. intracellulare, M. smegmatis, M. aurum and M. phlei	1.25	17389998
Acanthostrongylophora Sp.	Manadomanzamine B	M. tuberculosis H37Rv	1.5	17389998
Micromelum Hirsutum	Micromolide	M. tuberculosis H37Rv	1.5	17389998
Combretum Imberbe	Imberbic Acid	M. fortuitum	1.56	17389998
Acanthostrongylophora Sp.	Manadomanzamine A	M. tuberculosis H37Rv	1.9	17389998
Cryptolepis Sanguinolenta	Cryptolepine	M. aurum	2	17389998
Ruprechtia Triflora	New Acylated Endoperoxide	Mycobacterial species	2	17389998
Zopfiella Latipes	Zopfiellamides A	M. phlei	2	17389998
Verticillium Hemipterigenum	Enniatin H	M. tuberculosis H37Ra	3.12	17389998
Calophyllum Lanigerum	(+)-Calanolide A	M. tuberculosis	3.13	17389998
Streptomyces Sp.	Caprazamycin B353	M. tuberculosis, M. avium and M. intracellulare	3.13	17389998
Engelhardia Roxburghiana.	(?)-4-Hydroxy-1-Tetralone	M. tuberculosis	4	17389998
Calceolaria Pinnifolia	Diterpene 260	M. tuberculosis H37Rv	4	17389998
Calceolaria Pinnifolia	Diterpene 262	M. tuberculosis H37Rv	4	17389998
Cinnamomum Kotoense	Lauric Acid	Mycobacterial species	4	17389998

Sapium Haematospermum	Lecheronol A	M. tuberculosis	4	17389998
Ruprechtia Triflora	Peroxide 334	Mycobacterial species	4	17389998
Ruprechtia Triflora	Peroxide 335	Mycobacterial species	4	17389998
Piper Sanctum	Pyrone 55	M. tuberculosis H37Rv	4	17389998
Ruprechtia Triflora	Sterol Derivative 341	Mycobacterial species	4	17389998
Clathria (Thalysias) Abietina	Microcionamides A	M. tuberculosis H37Ra	5.7	17389998
Clathria (Thalysias) Abietina	Microcionamides B	M. tuberculosis H37Ra	5.7	17389998
Croton Kongensis	8,9-Secokaurane Diterpene 272	M. tuberculosis H37Ra	6.25	17389998
Croton Kongensis	8,9-Secokaurane Diterpene Derivative 273	M. tuberculosis H37Ra	6.25	17389998
Aplysina Gerardogreeni	Aerothionin	M. tuberculosis H37Rv	6.25	17389998
Piper Sanctum	Aryl Ketone 21	M. tuberculosis	6.25	17389998
Piper Sanctum	Aryl Ketone 22	M. tuberculosis	6.25	17389998
Bauhinia Saccocalyx	Bauhinoxepin A	M. tuberculosis H37Ra	6.25	17389998
Diospyros Ehretioides	Deoxypreussomerin Derivative 149	M. tuberculosis H37Ra	6.25	17389998
Verticillium Hemipterigenum	Enniatin I	M. tuberculosis H37Ra	6.25	17389998
An Unidentified Thai Fungus	Hydroxyl Analogues Enniatin L	Mycobacterial species	6.25	17389998
Machaerium Multiflorum	Phenol 137	M. intracellulare	7	17389998
Angelica Dahurica	3r,8s Stereoisomer Of Falcarindiol	Methicillin-resistant Staphylococcus aureus	8	17389998
Sapium Haematospermum	Cycloartanol	M. tuberculosis H37Rv	8	17389998
Calceolaria Pinnifolia	Diterpene 265	M. tuberculosis H37Rv	8	17389998
Pachychalina Sp.	Ingenamine G	M. tuberculosis H37Rv	8	17389998
Piper Argyrophylum	Piperolactam A	M. tuberculosis H37Rv	8	17389998

Calceolaria Pinnifolia	Triterpene 303	Mycobacterial species	8	17389998
Machaerium Multiflorum	Phenol 138	M. intracellulare	10	17389998
Pimpinella Sp.	Phenylpropanoid 31	M. intracellulare, M. smegmatis, M. aurum and M. phlei	10	17389998
Zopfiella Latipes	Zopfiellamides B	M. phlei	10	17389998
Piper Argyrophylum	Cepharanone B	M. tuberculosis H37Rv	12	17389998
Senna Oblique	Quinquangulin	M. tuberculosis (ATCC 27294)	12	17389998
Senna Oblique	Rubrofasarin	M. tuberculosis (ATCC 27294)	12	17389998
Sapium Haematospermum	Oleanolic Acid Analogue 316	M. tuberculosis	12.2	17389998
Abrus Precatorius	Abruquinone B	M. tuberculosis H37Ra	12.5	17389998
Bauhinia Saccocalyx	Bauhinoxepin B	M. tuberculosis H37Ra	12.5	17389998
Casearia Grewiifolia Vent.	Caseargrewiin A	Mycobacterial species	12.5	17389998
Casearia Grewiifolia Vent.	Caseargrewiin B	Mycobacterial species	12.5	17389998
Casearia Grewiifolia Vent.	Caseargrewiin D	Mycobacterial species	12.5	17389998
Casearia Grewiifolia Vent.	Congener 278	Mycobacterial species	12.5	17389998
Casearia Grewiifolia Vent.	Congener 279	Mycobacterial species	12.5	17389998
P. Elisabethae	Homopseudopteroxazole	M. tuberculosis H37Rv	12.5	17389998
Combretum Imberbe	Hydroxyimberbic Glycosiden 310	M. fortuitum	12.5	17389998
An Unidentified Thai Fungus	Hydroxyl Analogues Enniatin M	Mycobacterial species	12.5	17389998
Artocarpus Lakoocha	Lakoochins A	M. tuberculosis H37Ra	12.5	17389998
Cinnamomum Kotoense	Vanillin	M. tuberculosis strain 90-221388	12.5	17389998
Sapium	Compound 307	M. tuberculosis	13.4	17389998

Haematospermum				
Clausena Excavata	Substituted Carbazole 232	Mycobacterial species	14.3	17389998
Valeriana Laxiflora	24-Hydroxyursolic Acid	M. tuberculosis H37Rv	15.5	17389998
Clausena Excavata	Substituted Carbazole 236	Mycobacterial species	15.6	17389998
Calceolaria Pinnifolia	Diterpene 264	M. tuberculosis H37Rv	16	17389998
Warburgia Ugandensis	Muzigadial	M. aurum, M. fortuitum, M. phlei and M. smegmatis	16	17389998
Iostephane Heterophylla	Phenol 50	M. tuberculosis H37Rv	16	17389998
Cosmos Pringlei	Sesquiterpene Lactone 301	M. tuberculosis H37Rv	16	17389998
Calceolaria Pinnifolia	Triterpene 304	Mycobacterial species	16	17389998
Machaerium Multiflorum	Benzopyran	M. intracellulare	20	17389998
Diospyros Maritima	Chitranone	M. smegmatis	20	17389998
Piperaff. Pedicellatum	(+)-Bornyl Piperate	M. tuberculosis H37Ra	25	17389998
Croton Kongensis	8,9-Secokaurane Diterpene 271	M. tuberculosis H37Ra	25	17389998
Casearia Grewiifolia Vent.	Caseargrewiin C	Mycobacterial species	25	17389998
Prismatomeris Fragrans	Compound 160	M. tuberculosis H37Ra	25	17389998
Geotrichum Sp.	Dihydroisocoumarin 79	M. tuberculosis H37Ra	25	17389998
Combretum Imberbe	Hydroxyimberbic Glycosiden 311	M. fortuitum	25	17389998
Combretum Imberbe	Hydroxyimberbic Glycosiden 312	M. fortuitum	25	17389998
Cinnamomum Kotoense	Syringaldehyde	M. tuberculosis strain 90-221387	25	17389998
Valeriana Laxiflora	Betulin	M. tuberculosis	30	17389998
Mitrephora Glabra	New Diterpene 270	M. smegmatis	31	17389998
Clausena Excavata	Substituted Carbazole 231	Mycobacterial species	31.5	17389998
Morinda Citrifolia	(E)-Phytol	M. tuberculosis	32	17389998

		H37Rv		
Piper Sanctum	Aryl Ketone 25	M. tuberculosis	32	17389998
Piper Argyrophylum	Cepharadione B	M. tuberculosis H37Rv	32	17389998
Morinda Citrifolia	Compound 330	M. tuberculosis H37Rv	32	17389998
Calceolaria Pinnifolia	Diterpene 259	M. tuberculosis H37Rv	32	17389998
Piper Sanctum	Pyrone 54	M. tuberculosis H37Rv	32	17389998
Cosmos Pringlei	Sesquiterpene Lactone 300	M. tuberculosis H37Rv	32	17389998
Amphipteryngium Adstringens	Tirucallane-Skeleton Sterol 346	M. tuberculosis H37Rv	32	17389998
Euphorbia Peplis	Cerebroside	M. tuberculosis	40	17389998
Clausena Excavata	Substituted Carbazole 235	Mycobacterial species	42.3	17389998
Valeriana Laxiflora	Flavone 97	Mycobacterial species	46.2	17389998
Prismatomeris Fragrans	3-Acetyloleanolic Acid Derivative 320	M. tuberculosis H37Ra	50	17389998
Prismatomeris Fragrans	Compound 163	M. tuberculosis H37Ra	50	17389998
Larrea Divaricata	Compound 53	M. tuberculosis H37Rv	50	17389998
Pterocaulon Redolens	Coumarin 64	M. tuberculosis H37Rv	50	17389998
Geotrichum Sp.	Dihydroisocoumarin 80	M. tuberculosis H37Ra	50	17389998
Larrea Divaricata	Ermanin 94	M. tuberculosis H37Rv	50	17389998
Larrea Divaricata	Ermanin 96	M. tuberculosis H37Rv	50	17389998
Kaempferia Parviflora	Flavonoid 107	Mycobacterial species	50	17389998
Hirsutella Nivea Bcc 2594	Hirsutatins A	M. tuberculosis H37Ra	50	17389998
Hirsutella Nivea Bcc 2595	Hirsutatins B	M. tuberculosis H37Ra	50	17389998
Artocarpus Lakoocha	Lakoochins B	M. tuberculosis H37Ra	50	17389998
Menisporopsis Theobromae	Menisporopsin A	M. tuberculosis H37Ra	50	17389998

Clausena Excavata	Substituted Carbazole 227	Mycobacterial species	50	17389998
Valeriana Laxiflora	Flavone 98	Mycobacterial species	58.5	17389998
Valeriana Laxiflora	Betulinic Acid	M. tuberculosis	62.1	17389998
Mitrephora Glabra	New Diterpene 268	M. smegmatis	63	17389998
Celaenodendron Mexicanum	Biflavonoid 112	M. tuberculosis H37Rv	64	17389998
Calceolaria Pinnifolia	Diterpene 258	M. tuberculosis H37Rv	64	17389998
Calceolaria Pinnifolia	Diterpene 261	M. tuberculosis H37Rv	64	17389998
Artemisia Monosperma	Eriodyctiol-7-Methyl Ether 110	M. aurum	64	17389998
Fischerella Ambigua	Hexachlorodiphenylether Metabolite	M. tuberculosis	64	17389998
Sapium Haematospermum	Oleanolic Acid Analogue 317	M. tuberculosis	64	17389998
Ducrosia Anethifolia	Pangelin	Mycobacterial species	64	17389998
Amphipteryngium Adstringens	Tirucallane-Skeleton Sterol 345	M. tuberculosis H37Rv	64	17389998
Piper Sanctum	Z-Piperolide 29	M. tuberculosis	64	17389998
Diospyros Maritima	Maritinone	M. smegmatis	80	17389998
Mitrephora Glabra	New Diterpene 269	M. smegmatis	88	17389998
Pterocaulon Redolens	Coumarin 63	M. tuberculosis H37Rv	100	17389998
Pterocaulon Redolens	Coumarin 65	M. tuberculosis H37Rv	100	17389998
Pterocaulon Redolens	Coumarin 66	M. tuberculosis H37Rv	100	17389998
Pterocaulon Redolens	Coumarin 67	M. tuberculosis H37Rv	100	17389998
Pterocaulon Redolens	Coumarin 68	M. tuberculosis H37Rv	100	17389998
Euclea Natalensis	Diospyrin	M. tuberculosis	100	17389998
Chamaedora Tepejilote	Hydrocarbon Squalene	M. tuberculosis	100	17389998
Morinda Citrifolia	Compound 329	M. tuberculosis H37Rv	128	17389998
Rumex Hymenosepalus	Compound 51	M. tuberculosis H37Rv	128	17389998
Rumex Hymenosepalus	Compound 52	M. tuberculosis H37Rv	128	17389998
Calceolaria Pinnifolia	Diterpene 263	M. tuberculosis	128	17389998

		H37Rv		
Sapium Haematospermum	Lecheronol B	M. tuberculosis	128	17389998
Ruprechtia Triflora	Triterpene 302	Mycobacterial species	128	17389998
Newbouldia Laevis	Canthic Acid	Bacillus subtilis and B. cerus	0.038	20111803
Streptomyces Platensis	Platensimycin	Methicillin-resistant Staphylococcus aureus	0.1	20111803
Dorstenia Barteri	Isobavachalcone	Enterobacter cloacae, Streptococcus faecalis, Staphylococcus aureus, Bacillus stearothermophilus, Candida albicans and C. glabrata	0.3	20111803
Newbouldia Laevis	Newbouldiaquinone A	Gram-negative bacteria	0.31	20111803
Erythrina Subumbrans	Erycristagallin	S. aureus	0.39	20111803
Lysobacter Sp.	Tripropeptin C	Methicillin-resistant Staphylococcus aureus	0.39	20111803
Lysobacter Sp.	Tripropeptin D	Methicillin-resistant Staphylococcus aureus	0.39	20111803
Ficus Chlamydocarpa	Laburnetin	M. smegmatis	0.61	20111803
Erythrina Subumbrans	Erybraedin A	Streptococcus strains	0.78	20111803
Anthyllis Vulneraria	Pleosporone	Haemophilus influenzae	1	20111803
Newbouldia Laevis	Chrysoeriol	Four Gram-positive and ten Gram-negative bacterial species	1.2	20111803
Vismia Laurentii	Xanthones 139	Bacillus subtilis and Candida	1.2	20111803

		glabrata		
Rheedia Brasiliensis	7-Epi-Clusianone	Streptococcus mutans	1.25	20111803
Xenia Novaebrittanniae	Xeniolide I	Escherichia coli and Bacillus subtilis	1.25	20111803
Erythrina Subumbrans	Erythrabyssin Ii	Streptococcus strains	1.56	20111803
Piper Regnelli	Eupomatenoid-6	S. aureus	1.56	20111803
Vitis Sp.	Heyneanol A	Methicillin-resis tant Staphylococcus aureus	2	20111803
Cratoxylum Formosum	Formoxanthone C	S. aureus	2.3	20111803
Vismia Laurentii	Fridelin	Six Gramnegative, four Gram-positive and two fungal strains	2.4	20111803
Vismia Laurentii	Kaempferol	Two Gram-negative and four Gram-positive pathogens	2.4	20111803
Lycium Chinense	(+)-Lyoniresinol-3a-O-B-D-Glucopyrano side	S. aureus	2.5	20111803
Merulius Incarnatus	5-Alkylresorcinol 68	Methicillin-resis tant Staphylococcus aureus	2.5	20111803
Bionectra Byssicola F120	Verticillin G	S. aureus	3	20111803
Piper Regnelli	Eupomatenoid-5	S. aureus	3.12	20111803
Scutellaria Barbata	Apigenin	Methicillin-resis tant Staphylococcus aureus	3.9	20111803
A Leaf-Litter Fungus Of The Phoma Genus	Phomallenic Acid A	S. aureus	3.9	20111803
Garcinia Cowa	Mangostanin	S. aureus	4	20111803
Cratoxylum Formosum	Gerontoxanthone I	S. aureus	4.6	20111803
Cratoxylum Formosum	Macluraxanthone	S. aureus	4.6	20111803

Vismia Laurentii	Compound 100	Streptococcus faecalis and Morganella morgani	4.8	20111803
Dorstenia Barteri	Kanzonol C	E. aerogens, E. cloacae, M. morganii, S. flexneri, S. faecalis, B. megaterium, B. stearothermophilus, C. albicans and C. glabrata	4.9	20111803
Saccharothrix Sp.	Mutactimycin C	Microccocus leteus and Klebsiella pneumoniae	5	20111803
Phomopsis Sp. Hzla01-1	Phomolides A	E. Coli	5	20111803
Eremophila Serrulata	9-Methyl-3-(4-Methyl-3-Pentenyl)-2,3-Dihydronaphtho[1,8-Bc]Pyran-7,8-Dione	Staphylococcus aureus ATCC 25923, Streptococcus pneumoniae ATCC 49619 and Streptococcus pyogenes ATCC 10389	7.8	20111803
A Leaf-Litter Fungus Of The Phoma Genus	Phomallenic Acid B	S. aureus	7.8	20111803
A Leaf-Litter Fungus Of The Phoma Genus	Phomallenic Acid C	S. aureus	7.8	20111803
Garcinia Cowa	Amangostin	S. aureus	8	20111803
Anthyllis Vulneraria	Phaeosphenone	Gram-positive bacterial species	8	20111803
Salvia Officinalis	Ursolic Acid	Methicillin-resistant Staphylococcus aureus	8	20111803
Newbouldia Laevis	2-Acetylfuro-1,4-Naphthoquinone	Six Gram-positive and twelve Gram-negative bacterial species	9.76	20111803

Newbouldia Laevis	2-Hydroxy-3-Methoxy-9,10-Dioxo-9,10-Dihydroanthracene-1-Carbaldehyde	Six Gram-positive and twelve Gram-negative bacterial species	9.76	20111803
Irvingia Gabonensis	3,30,40-Tri-Omethylellagic Acid	E. coli, Proteus vulgaris and B. subtilis	9.76	20111803
Irvingia Gabonensis	3,4-Di-O-Methylellagic Acid	E. coli, Proteus vulgaris and B. subtilis	9.76	20111803
Newbouldia Laevis	Lapachol	Six Gram-positive and twelve Gram-negative bacterial species	9.76	20111803
Ginkgo Biloba	7-Amino-4-Methylcoumarin	S. aureus, E. coli	10	20111803
Bionectra Byssicola F120	Bionectins A	S. aureus	10	20111803
Aspilia Foliacea	Ent-Kaur-16(17)-En-19-Oic Acid	Streptococcus sobrinus, S. mutans, S. mitis, S. sanguinis and Lactobacillus casei	10	20111803
Phomopsis Sp. Hzla01-1	Phomolides B	E. Coli	10	20111803
Chondromyces Crocatus	Ajudazols A	Micrococcus luteus	12.5	20111803
Chondromyces Crocatus	Ajudazols B	Micrococcus luteus	12.5	20111803
Psoralea Corylifolia	Psoracorylifols A	Helicobacter pylori (SS1 and ATCC 43504)	12.5	20111803
Merulius Incarnatus	5-Alkylresorcinol 69	Methicillin-resistant Staphylococcus aureus	15	20111803
Salvia Officinalis	Oleanolic Acid	Methicillin-resistant Staphylococcus aureus	16	20111803
Curcuma Xanthorrhiza	Xanthorrhizol	S. aureus	16	20111803

Psoralea Corylifolia	Psoracorylifols B	Helicobacter pylori (SS1 and ATCC 43504)	25	20111803
Bionectra Byssicola F120	Bionectins B	S. aureus	30	20111803
Nuraria Picta	Compound 1	P. vulgaris	12.5	M. Shahid, A. Shahzad, F. Sobia, A. Sahai, T. Tripathi, A. Singh, H.M. Khan and Umesh. Plant Natural Products as a Potential Source for Antibacterial Agents: Recent Trends. Anti-Infective Agents in Medicinal Chemistry, 2009, 8, 211-225
Nuraria Picta	Compound 2	S. aureus	12.5	M. Shahid, A. Shahzad, F. Sobia, A. Sahai, T. Tripathi, A. Singh, H.M. Khan and Umesh. Plant Natural Products as a Potential Source for Antibacterial Agents: Recent Trends. Anti-Infectiv

				e Agents in Medicinal Chemistry, 2009, 8, 211-225
Nuraria Picta	Compound 4	S. aureus	12.5	M. Shahid, A. Shahzad, F. Sobia, A. Sahai, T. Tripathi, A. Singh, H.M. Khan and Umesh. Plant Natural Products as a Potential Source for Antibacterial Agents: Recent Trends. Anti-Infective Agents in Medicinal Chemistry, 2009, 8, 211-225

Stemona Tuberosa	Compound 8	B. pumilis	12.5	M. Shahid, A. Shahzad, F. Sobia, A. Sahai, T. Tripathi, A. Singh, H.M. Khan and Umesh. Plant Natural Products as a Potential Source for Antibacterial Agents: Recent Trends. Anti-Infective Agents in Medicinal Chemistry, 2009, 8, 211-225
Nuraria Picta	Compound 6	E coli, C. albicans	12.5	M. Shahid, A. Shahzad, F. Sobia, A. Sahai, T. Tripathi, A. Singh, H.M. Khan and Umesh. Plant Natural Products as a Potential Source for Antibacterial Agents: Recent Trends. Anti-Infective Agents in Medicinal Chemistry, 2009, 8, 211-225

Eremophila Serrulata	Compound 2	S. aureus	15.6	M. Shahid, A. Shahzad, F. Sobia, A. Sahai, T. Tripathi, A. Singh, H.M. Khan and Umesh. Plant Natural Products as a Potential Source for Antibacterial Agents: Recent Trends. Anti-Infective Agents in Medicinal Chemistry, 2009, 8, 211-225
Nuraria Picta	Compound 7	S. aureus	200	M. Shahid, A. Shahzad, F. Sobia, A. Sahai, T. Tripathi, A. Singh, H.M. Khan and Umesh. Plant Natural Products as a Potential Source for Antibacterial Agents: Recent Trends. Anti-Infective Agents in Medicinal Chemistry, 2009, 8, 211-225

Eremophila Serrulata	Compound 5	S. aureus	250	M. Shahid, A. Shahzad, F. Sobia, A. Sahai, T. Tripathi, A. Singh, H.M. Khan and Umesh. Plant Natural Products as a Potential Source for Antibacterial Agents: Recent Trends. Anti-Infective Agents in Medicinal Chemistry, 2009, 8, 211-225
Bionectra Byssicola F120	Verticillin D	S. aureus	3	17190469
Bionectra Byssicola F120	Bionectin A	S. aureus	10	17190469
Bionectra Byssicola F120	Bionectin B	S. aureus	30	17190469
Cannabis Satiwa	Cannabidiol	S. aureus	0.5	18681481
Cannabis Satiwa	Tetrahydrocannabinol	S. aureus	0.5	18681481
Cannabis Satiwa	Cannabichromene	S. aureus	1	18681481
Cannabis Satiwa	Cannabigerol	S. aureus	1	18681481
Cannabis Satiwa	Cannabinol	S. aureus	1	18681481
Cannabis Satiwa	Pre-Cannabidiol	S. aureus	2	18681481
Cannabis Satiwa	Pre-Cannabigerol	S. aureus	2	18681481
Cannabis Satiwa	Pre-Tetrahydrocannabinol	S. aureus	4	18681481
Cannabis Satiwa	Carmagerol	S. aureus	16	18681481
Cannabis Satiwa	Compound 3f	S. aureus	64	18681481
Cannabis Satiwa	Olivetol	S. aureus	64	18681481
Marine Actinomycete Strain Nps008920, A Member Of The New Genus Marinispora	Lipoxazolidinone A	Staphylococcus aureus ATCC 29213 (MSSA)	0.9	17845000

Marine Actinomycete Strain Nps008920, A Member Of The New Genus Marinispora	Lipoxazolidinone B	Staphylococcus aureus ATCC 43300 (MRSA)	1.5	17845000
Marine Actinomycete Strain Nps008920, A Member Of The New Genus Marinispora	Lipoxazolidinone C	Staphylococcus aureus ATCC 43300 (MRSA)	3	17845000
Marine Actinomycete Strain Nps008920, A Member Of The New Genus Marinispora	Hydrolysis Product Compound 4	Staphylococcus aureus ATCC 29213 (MSSA)	24	17845000
Streptomyces Vinaceus	Citreamicin ε	S. aureus	0.12	19053507
Streptomyces Vinaceus	Citreamicin δ	S. aureus	0.5	19053507
Myrtus Communis L.	Gallomyrtucommulone B	S. aureus	64	16499325
Myrtus Communis L.	Gallomyrtucommulone A	S. aureus	128	16499325
Amycolatopsis Fastidiosa	Thiazomycin	S. aureus	0.003	19334707
Amycolatopsis Fastidiosa	Thiazomycin B	S. aureus	0.02	19334707
Amycolatopsis Fastidiosa	Thiazomycin C	S. aureus	0.03	19334707
Amycolatopsis Fastidiosa	Thiazomycin D	S. aureus	0.03	19334707
Amycolatopsis Fastidiosa	Thiazomycin E1	S. aureus	100	19334707
Amycolatopsis Fastidiosa	Thiazomycins E3	E. faecalis, S. pneumoniae	200	19334707
Santalum Album	(Z)-α-Santalol	H. pylori	7.8	15974602
Santalum Album	(Z)-β-Santalol	H. pylori	7.8	15974602
Santalum Album	(Z)-Lanceol	H. pylori	31.3	15974602
Streptomyces Sp. Amri-33844	Neopyrrolomycin B	S. aureus	0.06	19191549
Streptomyces Sp. Amri-33844	Neopyrrolomycin C	S. aureus	1	19191549
Streptomyces Sp. Amri-33844	Neopyrrolomycin D	S. aureus	2	19191549
Elaphoglossum Yungense	Yungensin A	S. aureus	10	21043474
Elaphoglossum Yungense	Yungensin B	S. aureus	10	21043474
Elaphoglossum Yungense	Yungensin C	S. aureus	50	21043474

Elaphoglossum Yungense	Yungensin F	S. aureus	50	21043474
Elaphoglossum Yungense	Yungensin D	S. aureus	100	21043474
Elaphoglossum Yungense	Yungensin E	S. aureus	100	21043474
Stemodia Foliosa	6α-Malonoyloxymanoyl Oxide	S. aureus	15	18582112
Kitasatospora Spp.	Sch-725424	S. aureus	1	16503759
Amycolatopsis Orientalis	Eco-0501	S. aureus	4	16503759
Sorangium Cellulosum	Etnangien	S. aureus	1	17547459
Pacific Propolis	Propolin C	Methicillin-resistant Staphylococcus aureus	8000	20077439
Pacific Propolis	Propolin D	Methicillin-resistant Staphylococcus aureus	8000	20077439
Cryptomeria Japonica	Ferruginol	S. aureus	6.3	18780250
Cryptomeria Japonica	Isopimaric Acid	S. aureus	15.6	18780250
Cryptomeria Japonica	Sandaracopimarinol	S. aureus	31.3	18780250
Cryptomeria Japonica	Sugiol	E. faecalis, S. epidermidis	31.3	18780250
Cryptomeria Japonica	Iguestol	E. faecalis, S. epidermidis	250	18780250
Cryptomeria Japonica	Isopimarol	S. aureus	250	18780250
Magydaris Tomentosa	Citropten	S. aureus	16	17128388
Magydaris Tomentosa	Imperatorin	S. aureus	32	17128388
Magydaris Tomentosa	(+)-Meranzin Hydrate	S. aureus	128	17128388
Magydaris Tomentosa	Umbelliprenin	S. aureus	128	17128388
Angelica Gigas	Decursin	Bacillus subtilis	12.5	12877552
Angelica Gigas	Decursinol Angelate	Bacillus subtilis	50	12877552
Erythrina Poeppigiana	3,9-Dihyroxy-10-Gamma,Gamma-Dimethylallyl-6a,11a-Dehydropterocarpan	Methicillin-resistant Staphylococcus aureus	125	15185847
Erythrina Variegata	Erycristagallin	Methicillin-resistant Staphylococcus aureus	6.25	12460431
Erythrina Variegata	Orientanol B	Methicillin-resistant	6.25	12460431

		Staphylococcus aureus		
Erythrina Variegata	2-(γ,γ-Dimethylallyl)-6a-Hydroxyphaseollidin	Methicillin-resistant Staphylococcus aureus	12.5	12460431
Erythrina Variegata	Orientanol C	Methicillin-resistant Staphylococcus aureus	12.5	12460431
Erythrina Variegata	Orientanol F	Methicillin-resistant Staphylococcus aureus	12.5	12460431
Erythrina Variegata	Erystagallin A	Methicillin-resistant Staphylococcus aureus	25	12460431
Erythrina Variegata	Eryvarin C	Methicillin-resistant Staphylococcus aureus	25	12460431
Erythrina Variegata	Eryvarin D	Methicillin-resistant Staphylococcus aureus	25	12460431
Erythrina Variegata	Phaseollin	Methicillin-resistant Staphylococcus aureus	25	12460431
Erythrina Variegata	Folitenol	Methicillin-resistant Staphylococcus aureus	50	12460431
Erythrina Variegata	Phaseollidin	Methicillin-resistant Staphylococcus aureus	50	12460431
Erythrina Variegata	Cristacarpin	Methicillin-resistant Staphylococcus aureus	100	12460431
Erythrina Variegata	Erysubin F	Methicillin-resistant	100	12460431

		Staphylococcus aureus		
	Terthiophenes	S. aureus	0.022	15042149
Hypericum Perforatum	Hyperforin	S. aureus	0.1	15042149
Garcinia Dioica	Rubraxanthone	S. aureus	0.313	15042149
Ulmus Davidiana Var. Japonica	Mansinone F	S. aureus	0.39	15042149
Allium Sativum	Diallyl Tetrasulfide	S. aureus	0.5	15042149
Myrtus Communis	Myrtucommulone A	S. aureus	0.5	15042149
Crossopetalum Gaumeri	Nor-Friedelane Triterpenes	S. aureus	0.54	15042149
	Bornyl Coumarate	S. aureus	0.6	15042149
Trees Belonging To The Burseraceae Family	Curzerenone	S. aureus	0.7	15042149
	Compound 116	S. aureus	1	15042149
Crossopetalum Gaumeri	Nor-Friedelane Triterpenes	S. aureus	1.11	15042149
Salvia Blepharochlaena	Horminone	S. aureus	1.5	15042149
	Compound 74	S. aureus	1.56	15042149
	Flavanone	S. aureus	1.8	15042149
Artemisia Gilvescens	Guaianolide	S. aureus	1.95	15042149
Artemisia Asiatica	1,8-Cineole	S. aureus	2	15042149
Guatteria Multivenia	Aporphine	S. aureus	2	15042149
Piper Gibbilimbum	Gibbilimbol B	S. aureus	2	15042149
	Isopimarane	S. aureus	2	15042149
Clausena Heptaphylla	Compound 87	S. aureus	3	15042149
	Licochalcone A	S. aureus	3	15042149
Cassia Quinquangulata	Naphthopyrone	S. aureus	3.125	15042149
	Alopecurone B	S. aureus	3.13	15042149
Erythrina Species	Compound 81	S. aureus	3.13	15042149
	Compound 82	S. aureus	3.13	15042149
Erythrina Zeyheri	Pterocarpans 83	S. aureus	3.13	15042149
Erythrina Zeyheri	Pterocarpans 84	S. aureus	3.13	15042149
	Sophoraflavanone G	S. aureus	3.13	15042149
Zanthoxylum Clava-Herculis	Chelerythrine	S. aureus	4	15042149
	Full Lignans Of The Aryl Tetralin Class 58	S. aureus	4	15042149
Calophyllum Moonii	Calozeyloxanthone	S. aureus	4.1	15042149
	Compound 70	S. aureus	4.5	15042149
	Globulixanthones E	S. aureus	4.5	15042149
Helichrysum Caespititium	Compound 115	S. aureus	5	15042149

	Compound 76	S. aureus	5	15042149
Copaifera Paupera	Diterpene	S. aureus	5	15042149
Calycodendron Milnei	Isopsychotridine	S. aureus	5	15042149
	Acetylated Rhamnoside Oleanene	S. aureus	6.25	15042149
Ferula Species	Daucane Sesquiterpenes With Various Aromatic Ester Moieties	F. hermonis	6.25	15042149
Amomum Aculeatum	Aculeatin D	S. aureus	8	15042149
Ochna Macrocalyx	Compound 86	S. aureus	8	15042149
	Falcarindiol	S. aureus	8	15042149
	Globulixanthones D	S. aureus	8	15042149
Erythrina Species	Compound 79	S. aureus	8.3	15042149
Salvia Viridis	Isopimarane Type Diterpenes	S. aureus	9	15042149
Erythrina Species	3-Phenylcoumarin	S. aureus	9.7	15042149
Salvia Viridis	1-Oxoferruginol	S. aureus	10	15042149
Salvia Blepharochlaena	7-Acetylhorminone	S. aureus	10	15042149
Fabiana Densa Var. Ramulosa	Succinate Derivative	S. aureus	10	15042149
Styrax Ferrugineus	The Nor-Lignan	S. aureus	10	15042149
Viguiera Hypargyrea	Beyerenoic Acid	S. aureus	12	15042149
	Esters Of A Skeleton Possessing A Trans-P-Coumarate	S. aureus	12.5	15042149
Maytenus Undata	Koetjapic Acid	S. aureus	12.5	15042149
	Mahanine	S. aureus	12.5	15042149
Mitrephora Celebica	The Polyynes 101	S. aureus	12.5	15042149
Salvia Prionitis	Sanigerone	S. aureus	13	15042149
Cephalotaxus	Abietane Type Diterpenes	S. aureus	15	15042149
Chamaecyparis	Abietane Type Diterpenes	S. aureus	15	15042149
	Compound 85	S. aureus	15.3	15042149
Allium Species	Ajoene	S. aureus	16	15042149
	Xanthorrizol	S. aureus	16	15042149
Ballota Saxatilis Subsp.	Ballonigrine	S. aureus	25	15042149
Mitrephora Celebica	The Polyynes 100	S. aureus	25	15042149
Balsamorhiza Sagittata	Thiophene-Polyyne	S. aureus	25	15042149
Plectranthus Hereroensis	An Acetylated Abietane Quinone	S. aureus	31.2	15042149
Prangos Pabularia	Osthol	S. aureus	31.25	15042149
Hypericum Papuanum	Compounds 112 And 113	S. aureus	32	15042149
Acalypha Communis	Cycloartane Type Triterpenes	S. aureus	32	15042149
	Full Lignans Of The Aryl Tetralin Class 59	S. aureus	32	15042149
A Nepalese Medicinal Plant	Arnicolide C	S. aureus	38	15042149

Maytenus Undata	An 11-Ethoxy Derivative	S. aureus	50	15042149
	Compound 73	S. aureus	50	15042149
Helichrysum Species	Cyclised Labdane Diterpene Manoyl Oxide	S. aureus	50	15042149
	Luteolin	S. aureus	62.5	15042149
Verbascum Undulatum	Verbalactone	S. aureus	62.5	15042149
Green Tea	Compound P	S. aureus	280	15042149
Helichrysum Aureonitens	3,5,7-Trihydroxyflavone	S. aureus	0.1	18582553
Helichrysum Trilineatum	5,7-Dihydroxyflavanone	S. aureus	0.1	18582553
Helichrysum Caespititium	2-Methyl-4-[2 ,4 ,6 -Trihydroxy-3 -(2-Methylpropanoyl)Phenyl]But-2-Enyl Acetate	S. aureus	0.5	18582553
Helichrysum Trilineatum	2 ,4 ,6 -Trihydroxychalcone	S. aureus	1	18582553
Salvia Chamelaeagnea	7-O-Methyl-Epirosmanol	S. aureus	10	18582553
Helichrysum Kraussii	Kaurenoic Acid	S. aureus	10	18582553
Osmundaria Serrata	Lanosol Ethyl Ether	A. alternate	10	18582553
Helichrysum Pedunculatum	Linoleic Acid	Bacillus cereus and B. pumulis	10	18582553
Warburgia Salutaris	Muzigadial	S. aureus	12.5	18582553
Salvia Chamelaeagnea	Carnosol	S. aureus	20	18582553
Combretum Erythrophyllum	5-Hydroxy-7,4 -Dimethoxyflavone	Escherichia coli, S. sonnei, V. cholerae and Pseudomonas aeruginosa	25	18582553
Combretum Erythrophyllum	Genkwanin	Escherichia coli, S. sonnei, V. cholerae and Pseudomonas aeruginosa	25	18582553
Helichrysum Cymosum	Helihumulone	S. aureus	31	18582553
Aloe Ferox	Chrysophanol	S. aureus	31.3	18582553
Spirostachys Africana	D-Friedoolean-14-En-Oic Acid	S. aureus	50	18582553
Spirostachys Africana	Ent-3 -Hydroxy-Beyer-15-Ene-2-One	S. aureus	50	18582553
Aloe Ferox	Aloe Emodin	S. aureus	62.5	18582553
Aloe Ferox	Aloin	S. aureus	62.5	18582553
Combretum Erythrophyllum	Nidoanomalin	Staphylococcus epidermidis, Bacillus cereus and	63	18582553

		Pseudomonas aeruginosa		
Spirostachys Africana	Ent-2,6 -Dihydroxy-Norbeyer-1,4,15-Trien-3-One	S. aureus	100	18582553
Helichrysum Kraussii	Prenyl-Butrylphloroglucinol	S. aureus	100	18582553
Veronia Colorata	Vernodalol	S. aureus	100	18582553
Veronia Colorata	Vernolide	S. aureus	100	18582553
Schotia Latifolia	Catechin	S. aureus	125	18582553
Schotia Latifolia	Epicatechin	S. aureus	125	18582553
Ipomoea Tricolor Cav. , I. Orizabensis (Pelletan) Ledebour Ex Steudel	Orizabin X	S. aureus	4	16562846
Ipomoea Tricolor Cav. , I. Orizabensis (Pelletan) Ledebour Ex Steudel	Orizabin Xi	S. aureus	4	16562846
Ipomoea Tricolor Cav. , I. Orizabensis (Pelletan) Ledebour Ex Steudel	Orizabin Xiii	S. aureus	4	16562846
Ipomoea Tricolor Cav. , I. Orizabensis (Pelletan) Ledebour Ex Steudel	Orizabin Xvii	S. aureus	4	16562846
Ipomoea Tricolor Cav. , I. Orizabensis (Pelletan) Ledebour Ex Steudel	Orizabin Xiv	S. aureus	8	16562846
Ipomoea Tricolor Cav. , I. Orizabensis (Pelletan) Ledebour Ex Steudel	Orizabin Xvi	S. aureus	8	16562846
Ipomoea Tricolor Cav. , I. Orizabensis (Pelletan) Ledebour Ex Steudel	Orizabin Xx	S. aureus	8	16562846
Ipomoea Tricolor Cav. , I. Orizabensis (Pelletan) Ledebour Ex Steudel	Orizabin Xxi	S. aureus	8	16562846
Ipomoea Tricolor Cav. , I. Orizabensis (Pelletan) Ledebour Ex Steudel	Tricolorin A	S. aureus	8	16562846
Ipomoea Tricolor Cav. , I. Orizabensis (Pelletan) Ledebour Ex Steudel	Tricolorin E	S. aureus	8	16562846
Ipomoea Tricolor Cav. , I. Orizabensis (Pelletan)	Orizabin Xv	S. aureus	16	16562846

Ledebour Ex Steudel				
Ipomoea Tricolor Cav. , I. Orizabensis (Pelletan) Ledebour Ex Steudel	Tricolorin B	S. aureus	16	16562846
Ipomoea Tricolor Cav. , I. Orizabensis (Pelletan) Ledebour Ex Steudel	Tricolorin C	S. aureus	16	16562846
Ipomoea Tricolor Cav. , I. Orizabensis (Pelletan) Ledebour Ex Steudel	Tricolorin D	S. aureus	16	16562846
Ipomoea Tricolor Cav. , I. Orizabensis (Pelletan) Ledebour Ex Steudel	Orizabin Xviii	S. aureus	32	16562846
Ipomoea Tricolor Cav. , I. Orizabensis (Pelletan) Ledebour Ex Steudel	Scammonin I	S. aureus	32	16562846
Ipomoea Tricolor Cav. , I. Orizabensis (Pelletan) Ledebour Ex Steudel	Orizabin Xix	S. aureus	128	16562846
Ipomoea Tricolor Cav. , I. Orizabensis (Pelletan) Ledebour Ex Steudel	Scammonin Ii	S. aureus	128	16562846
Ipomoea Tricolor Cav. , I. Orizabensis (Pelletan) Ledebour Ex Steudel	Orizabin Ix	S. aureus	256	16562846
Ipomoea Tricolor Cav. , I. Orizabensis (Pelletan) Ledebour Ex Steudel	Orizabin Xii	S. aureus	256	16562846
Actinomycete Strain Cnq-525	Terpenoid Chloro-Dihydroquinone Compound 4	Methicillin-resistant Staphylococcus aureus	1.9	15974616
Actinomycete Strain Cnq-525	Terpenoid Chloro-Dihydroquinone Compound 1	Methicillin-resistant Staphylococcus aureus	1.95	15974616
Actinomycete Strain Cnq-525	Terpenoid Chloro-Dihydroquinone Compound 3	Methicillin-resistant Staphylococcus aureus	1.95	15974616
Actinomycete Strain Cnq-525	Terpenoid Chloro-Dihydroquinone Compound 2	Methicillin-resistant	15.6	15974616

		Staphylococcus aureus		
Hypericum Chinense	Aspidinol C	S. aureus	2	21043475
Hypericum Chinense	Aspidinol D	S. aureus	4	21043475
Calceolaria Pinifolia	Diterpene Compound 8	S. aureus	2	12608857
Calceolaria Pinifolia	Diterpene Compound 2	S. aureus	4	12608857
Calceolaria Pinifolia	Diterpene Compound 10	S. aureus	8	12608857
Calceolaria Pinifolia	Diterpene Compound 6	S. aureus	8	12608857
Calceolaria Pinifolia	Diterpene Compound 1	S. aureus	16	12608857
Calceolaria Pinifolia	Diterpene Compound 7	S. aureus	16	12608857
Calceolaria Pinifolia	Diterpene Compound 4	S. aureus	32	12608857
Calceolaria Pinifolia	Diterpene Compound 11	Bacillus subtilis	64	12608857
Calceolaria Pinifolia	Diterpene Compound 12	Bacillus subtilis	64	12608857
Acalypha Communis	7b,16b-Dihydroxy-1,23-Dideoxyjessic Acid	S. aureus	16	12088430
Acalypha Communis	15a-Hydroxymollic Acid	S. aureus	32	12088430
Acalypha Communis	16a-Hydroxymollic Acid	S. aureus	32	12088430
