# Supplementary material for: What Does It Take to Synergistically Combine Sub-Potent Natural Products into Drug-Level Potent Combinations?
Source: PLoS One. 2012 Nov 28;7(11):e49969. doi: 10.1371/journal.pone.0049969 (PMC3509152; doi:10.1371/journal.pone.0049969)
Supplement: Table S5 — Cell-based microbial inhibitory activity values of 99 antimicrobial natural product extracts. (PDF) [file pone.0049969.s005.pdf]

Supplementary Table S5 Cell-based microbial inhibitory activity values of 99 antimicrobial natural product extracts. References are provided below this Table.

**Natual Product Extracts**	**Against microbial species**	**MIC (μg/mL)**
Abuta grandifolia (Mard.) Sandwith.	S. aureus	8000
Achyrocline alata (Kunth) DC	S. aureus	8000
Adiantum concinnum Willd.	S. aureus	8000
Airpotato Yam Rhizome	Non resistant Staphylococcus aureus	25000
Alternanthera porrigens (Jacq.) Kuntze	S. aureus	16000
Baccharis sp	E Coli	2000
Baical Skullcap Root	Staphylococcus aureus Rosenbach	750
Banisteriopsis caapi (Spruce ex Grieseb.) Morton	E Coli	62.5
Bersama abyssinica Fresen.	S. aureus	1000
Bird cherry Prunus padus	Staphylococcus aureus, Lactobacillus plantarum, Proteus mirabilis	100
Borago officinalis L.	S. aureus	8000
Brosimum rubescens Taub.	S. aureus	4000
C. 6iridis Leaves	S. aureus	4000
Caesalpinia paipai Ruiz. & Pav.	S. aureus	1000
Caesalpinia sappan	Staphylococcus aureus	1600
Capparis scabrida Kunth	S. aureus	8000
Cassia fistula L.	S. aureus	1000
Catha edulis	Staphylococcus aureus Rosenbach, Bacillus subtilis	12
Chenopodium ambrosioides L.	S. aureus	8000
Chinese Liquorice	Staphylococcus aureus	3100
Chinese Taxillus Twig	Staphylococcus aureus	12500
Chrysanthemum indicum L.	Staphylococcus aureus Rosenbach	3120
Cineraria grandiflora	S. aureus	4550
Citrus limetta Risso	S. aureus	2000
Clutia abyssinixa var. usambarica	S. aureus	4000
Combertum molle R.Br.ex G.Don	S. aureus	1000
Coptis Chinensis	Staphylococcus aureus	8000
Corynaea crassa Hook.f.	S. aureus	2000
Croton lechleri Müll. Arg.	S. aureus	2000
Cuphea sp.	S. aureus	8000
D. sylatica Tuber bark	S. epidermis B. subtilis	2000
Dahurian Patrinia Herb	Not resistant Staphylococcus aureus	50000
Dioscorea trifida L.f.	S. aureus	4000
Diplostephium sagasteguii Cuatrec.	S. aureus, E Coli	8000

Discopodium peninervum Hochst	S. aureus	2000
Dracocephalum heterophyllum Benth	Staphylococcus aureus, Staphylococcus epidermidis, Enterococcus faecalis	1560
Eclipta prostrata	Not resistant Staphylococcus aureus	25000
Eucalyptus globulus Labill	S. aureus	8000
Eugenia obtusifolia Cambess.	Staphylococcus aureus Rosenbach	8
Evodia rutaecarpa (Juss.) Benth.	Not resistant Staphylococcus aureus	50000
Ferula communis L	S. aureus	250
Forsythia suspensa	Staphylococcus aureus Rosenbach	3900
Fructus broussonetiae	Not resistant Staphylococcus aureus	50000
Gamochaeta sp.	S. aureus	8000
Gansu aconite	Staphylococcus aureus	6250
Garden Burnet	Staphylococcus aureus	3100
Gardenia lutea Fres.	S. aureus	2000
Gentianella bicolor (Wedd.) J.S. Pringle	S. aureus	8000
H. odoratissimum	Gram-negative bacteria	10
Hedyosmum racemosum (Ruiz. & Pav.) G. Don.	S. aureus	8000
Herba Agrimoniae	Methicillin resistant Staphylococcus aureus	6250
Herba Violae	Not resistant Staphylococcus aureus	50000
Hura crepitans L.	S. aureus	1000
Hypericum laricifolium Juss.	S. aureus	160
Hyptis sidifolia (L’Her.) Briq.	S. aureus	1000
Ilex guayusa Loes.	S. aureus	16000
Japanese Honeysuckle	Staphylococcus aureus Rosenbach	50000
Japnanese St. John’sawort Herb	Not resistant Staphylococcus aureus	50000
Lippia adoensis Hochst. Ex Schau.	S. aureus	2000
M. comosus Leaves	S. aureus	2000
Malva parviflora L.	S. aureus	2000
Micronia salicifolia (Bonpl. ex Naud.) Naud.	S. aureus	62.5
Musk mallow	Staphylococcus aureus, Escherichia coli	10
Myrica salcifolia A. Rich	S. aureus	1000
Ochroma pyramidale (Cav. ex Lam.) Urb.	E Coli	1000
Olea europea subsp. Cuspidate	S. aureus	2000
Oreocallis grandiflora R. Br.	S. aureus	2000
Origanum oil	Staphylococcus aureus, Bacillus anthracis, Escherichia coli, Klebsiella pneumoniae, Helicobacter pylori	250
Otholobium mexicanum (L.f.) Grimes	S. aureus	8000
Pelargonium odoratissimum Soland. cf.	S. aureus	2000
Phoradendron cf.	S. aureus	2000

Picrorhiza	Not resistant Staphylococcus aureus	50000
Polygonum hydropiperoides Michaux cf.	S. aureus	1000
Polylepis racemosa Ruiz. & Pav.	S. aureus	8000
Porophyllum ruderale Less.	S. aureus	4000
Portulaca grandiflora Hook.	Methicillin resistant Staphylococcus aureus	6250
Portulaca grandiflora Hook.	Not resistant Staphylococcus aureus	25000
Radix sophorae tonkinensis	Not resistant Staphylococcus aureus	50000
Ramulus uncariae cum uncis	Not resistant Staphylococcus aureus	50000
Rhubarb	Staphylococcus aureus	6200
Salvia sp.	S. aureus	1000
Sanguisorba minor Scop.	S. aureus	4000
Satureja pulchella (Kunth.) Briq.	S. aureus	2000
Seheciocannabifolius less	Staphylococcus aureus Rosenbach, Bacillus subtilis	10
Semen litchi	Staphylococcus aureus	12500
Senecio sp.	S. aureus	2000
Senna bicapsularis (L.) Roxb.	Escherichia coli	16
Senna monilifera H.S. Irwin & Barnaby	S. aureus	4000
Sophora Flavescens	Staphylococcus aureus	6000
Spartium junceumL.	S. aureus	4000
Syzygium aromaticum (L) Merr. & L.M. Perry	S. aureus	2000
Syzygium jambos (L.) Alston	S. aureus	8000
Trichila emetica Vahl	S. aureus	1000
V. colorata Leaves	S. aureus	500
Valeriana sp. cf.	S. aureus	8000
Vallesia glabra (Cav.) Link	S. aureus	16000
Vangueria infausta	S. aureus	1800
Verbena litoralis Kunth.	S. aureus	2000
Wild mignonette	Staphylococcus aureus, Bacillus prodigiosus	100

References
Preuss HG, Echard B, Enig M, Brook I, Elliott TB. Minimum inhibitory concentrations of herbal essential oils and monolaurin for gram-positive and gram-negative bacteria. Mol Cell Biochem. 2005 Apr;272(1-2):29-34.
Wu B, Wu LJ, Zhang L, Kim CS. Studies on the antibacterial chemical constituents of Senecio ca nnabif olius Less. Journal of Shenyang Pharmaceutical University. 2004 Sep;121(15), 1341.

Kelmanson JE, Jäger AK, van Staden J. Zulu medicinal plants with antibacterial activity. J Ethnopharmacol. 2000 Mar;69(3):241-6.

de Boer HJ, Kool A, Broberg A, Mziray WR, Hedberg I, Levenfors JJ. Anti-fungal and anti-bacterial activity of some herbal remedies from Tanzania. J Ethnopharmacol. 2005 Jan 15;96(3):461-9.

Geyid A, Abebe D, Debella A, Makonnen Z, Aberra F, Teka F, Kebede T, Urga K, Yersaw K, Biza T, Mariam BH, Guta M. Screening of some medicinal plants of Ethiopia for their anti-microbial properties and chemical profiles. J Ethnopharmacol. 2005 Mar 21;97(3):421-7.

Kumarasamy Y, Cox PJ, Jaspars M, Nahar L, Sarker SD. Screening seeds of Scottish plants for antibacterial activity. J Ethnopharmacol. 2002 Nov;83(1-2):73-7.

Bussmann RW, Malca-García G, Glenn A, Sharon D, Chait G, Díaz D, Pourmand K, Jonat B, Somogy S, Guardado G, Aguirre C, Chan R, Meyer K, Kuhlman A, Townesmith A, Effio-Carbajal J, Frías-Fernandez F, Benito M. Minimum inhibitory concentrations of medicinal plants used in Northern Peru as antibacterial remedies. J Ethnopharmacol. 2010 Oct 28;132(1):101-8.

Li R,Yan HF,Yu SL. In vitro antibacterial test of Lanqin Injection, Journal of Traditional Chinese Veterinary Medicine, 2006 6.

Luo HY, Kuang ZY, Huang YL, Sun DM, Huang SW, Li JT. Comparison of in vitro antibacterial tests of Scutellaria baicalensis particles and decoction. Chinese Journal of Clinical Rational Drug Use. 2011 Jan, 4(1).

Chen GH,Wu T,Huang QS,Chang GF,Zhong RS,Yu Q. Study Antibacterial Effects by Compared Baicalin and Baicalen sis Extracting Solution with Anti - MRSA IgY. Journal of Yichun College.2009 Apr 131(12).

Lin JQ,Kuang ZY,Huang SW,Luo HY. Observations from in vitro antibacterial tests on Forsythia suspense particles and decoction. CHINA FOREIGN MEDICAL TREATMENT, 2011,10.

Zhang CJ,He XB, Du FK, Liu AP,Li HY. Antibacterial Activity of Eight Tibetan Medicinal Plants in Vitro. Chin JMAP, 2010 February, Vol.27 No.2

Fu RQ, Meng DS, Lu LC. Antibacterial Effects of 21 Extracts of Chinese Herbal Medicine on MRSA. China Pharmacy 2011, 22(43).

Zuo GY,Wang GC,Wu GL,Zuo GY,Wang GC,Xu GL, et al. Screen of anti-MRSA activity from 30 Chinese herbage medicine extracts. Chin JMAP, 2006 August 23(4).

Yang HX,Ma QY,Zheng ZF. Study on antimicrobial activities of the extract of seven kinds of Chinese herbs rich in alkaloid. Journal of Zhengzhou University (Medical Sciences) 2004 Sep 39(5).

Yang SW.Comparative Study on the Antibacterial Activity of 32 kinds of Chinese Herbal Medicine. Journal of Anhui Agri. Sci. 2011，39(3) : 1361 — 1362，1366
