# Supplementary material for: What Does It Take to Synergistically Combine Sub-Potent Natural Products into Drug-Level Potent Combinations?
Source: PLoS One. 2012 Nov 28;7(11):e49969. doi: 10.1371/journal.pone.0049969 (PMC3509152; doi:10.1371/journal.pone.0049969)
Supplement: Table S7 — Targets and potency-enhancing synergistic molecular modes in 3 fully or partially sub-potent natural product combinations with group potencies improved to drug levels. (PDF) [file pone.0049969.s007.pdf]

Supplementary Table S7 Targets and potency-enhancing synergistic molecular modes in 3 fully or partially sub-potent natural product combinations with group potencies improved to drug levels

Ingredient	Role in Combination	Dose Reduction Index	Target, Therapeutic Effect or Response (reference in Pubmed ID)	Effect type	Potency-Enhancing Synergistic Modes (reference in Pubmed ID)	Type of Synergism
**Combination 1**						
**Tetraarsenic tetrasulfide (1.1uM)**	**Principal therapeutic component**	**6.88**	degraded the PML-RAR oncoprotein leading to anticancer effect (18344322), which may also hinder the possible antagonistic effect of RAR on TGF-alpha induced growth inhibition (12527889)	Anticancer, growth inhibition, via RAR	Indirubin blocked VEGFR2-mediated JAK/STAT3 signaling (21207415), which partially hindered RAR-STAT3 crosstalk and its promotion of apoptosis resistance (14959844) and transcription activation (15044588), thereby enhancing tetraarsenic tetrasulfide's anticancer effect	Complementary action
					Tanshinone IIA reduced and antagonized androgen receptor (AR) (22175694, 22281759, 21997969) to hinders its effect on the upregulation of RAR (12069693), thereby adding on tetraarsenic tetrasulfide's action in reducing PML-RAR	Complementary action

			down-regulated CDK2 in NB4 and NB4-R2 cells (18344322)	Anticancer, cell cycle regulation	Indirubin inhibited and down-regulated CDK2 (18344322) to complement tetraarsenic tetrasulfide's action in reducing CDK2	Complementary action
			upregulated RING-type E3 ligase c-CBL, leading to degradation of BCR-ABL (21118980)	Anti-leukemia, growth inhibition		
			tetraarsenic tetrasulfide tranported into tumor cell by AQP9 (18344322)	Intracellular bioavailability	Indirubin upregulated APQ9 (18344322) to enhance Tetraarsenic tetrasulfide's bioavailability	Intracellular bioavailability enhancement
					Tanshinone IIA (T) upregulated APQ9 (18344322) to enhance Tetraarsenic tetrasulfide's bioavailability	Intracellular bioavailability enhancement
			Reduction in RAR alpha may lead to P53 downregulation and Bcl-2 upregulation, thereby coutering anticancer effect (10675490)	Counteractive action against anticancer effect	Tanshinone IIA's activation of p53 signaling (21997969) may help against thie counteractive action	Anti-counteractive action
Indirubin (>3uM)	Cooperative	>9.38	inhibited and down-regulated CDK2, leading to anticancer effect (18344322)	Anticancer, cell cycle regulation	Tetraarsenic tetrasulfide reduced CDK2 (18344322), thereby complementing indirubin's action on CDK2	Complementary action
			inhibited GSK-3, thereby blocked its effect on tumor proliferation and migration (21697283)	Anticancer, growth inhibition		

			inhibited angiogenesis via blocking VEGFR2-mediated JAK/STAT3 signaling (21207415), which also partially hindered RAR-STAT3 crosstalk and its action on apoptosis resistance (14959844) and transcription activation (15044588), leading to anticancer effect	Anticancer, growth and angiogenesis inhibition, via RAR partner and additional growth and angiogenesis signaling		
			activated AhR (20951181), and AhR activation may lead to the activation of RAR alpha in the absence of ligand, thereby countering anticancer effect (16480812)	Counteractive action against anticancer effect, via RAR regulator	Tetraarsenic tetrasulfide degraded the PML-RAR oncoprotein (18344322), which may patially alleviate the conteractive action	Anti-counteractive action
Tanshinone IIA (>3uM)	Cooperative	>9.38	increased Bax/Bcl-2 ratio and caspase 3, decreased Bcl-2, mitochondrial membrane potential, MMPs and CD31, leading to anticancer apoptosis effects (21472292, 22002472, 22126901)	Anticancer, apoptosis		
			activated p53 signaling to promote anticancer effect (21997969)	Anticancer, cell cycle regulation, apoptosis		
			reduced survivin, ERCC1 and LRP via upregulation of phospho-P38, leading to enhanced apoptosis and anticancer effect (21165580)	Anticancer, apoptosis		

			reduced HER2, NF-κBp65 and LC3-II, leading to anticancer effect particularly against breast cancer (22246196), NF-κBp65 downregulation further hindered the effect of the binding of NF-κBp65 and RAR alpha on transcriptional regulaiton (17451432), which further contributes the ingredient's anticancer effects	Anticancer, apoptosis, growth inhibition, via RAR regulator and additional growth and survival signaling		
			reduced and antagonized androgen receptor (AR) and induced apoptosis, leading to anticancer effect against prostate cancer (22175694, 22281759, 21997969)	Anticancer, growth inhibition		
			upregulated phospho-P38 (21165580), which may help directing RAR alpha to its target promoters (19078967) and to subsequently cooperate with other cancer proteins for effective transcriptional activity in certain cancers (20080953), thereby countering anticancer effect	Counteractive action against anticancer effect	Tetraarsenic tetrasulfide degraded the PML-RAR oncoprotein (18344322), which may patially alleviate the conteractive action	Anti-counteractive action
			increased efflux transporters, which may help effluxing the ingredient (a Pgp substrate), thereby lowering its bioavailiability (17504222, 20821829)	Efflux-mediated multidrug resistance	Indirubin may inhibit certain efflux pump (20380543) to reduce the efflux of Tanshinone IIA	Intracellular bioavailability enhancement

**Combination 2**						
**Theaflavin (0.943ug/mL)**	**Principal therapeutic component**	**9.33**	Rotavirus activated JNK and p38 signaling pathways for enhanced viral replication (16928761), theaflavin reduced JNK and P38 phosphorelation (21184129, 22111069), which hinders viral replication and leads to virus neutralisation	Antiviral, against two of the 4 redundant viral replication regulators	The four ingredients target 4 redundant viral relication regulators, leading to strong synergistic antiviral activity, such activity is further enhanced by athways that mediate viral survival, growth and cell entry	Complementary action against redundant regulators
theaflavin-3-monogallate (251.39ug/mL)	Cooperative	2489	Rotavirus activated Cox2 to mediate viral infection at a postbinding step (15331705) probably including viral replication (17555580), theaflavin-3-monogallate and theaflavin-3'-monogallate mixture downregulated Cox2 (11103814), which hinders viral replication and leads to virus neutralisation	Antiviral, against one of the 4 redundent viral replication regulators		
theaflavin-3'-monogallate (5.07ug/mL)	Cooperative	50.2	Rotavirus activated Cox2 to mediate viral infection at a postbinding step (15331705) probably including viral replication (17555580), theaflavin-3-monogallate and theaflavin-3'-monogallate mixture downregulated Cox2 (11103814), which hinders viral replication and	Antiviral, against one of the 4 redundent viral replication regulators		

			leads to virus neutralisation			
theaflavin-3,3' digallate (5.51ug/mL)	Cooperative	54.6	Rotavirus activated ERK signaling pathways for enhanced viral replication (17689685), theaflavin-3,3' digallate reduced ERK phosphorelation (11511526), which hinders viral replication and leads to virus neutralisation	Antiviral, against one of the 4 redundent viral replication regulators		
			Rotavirus activated NFkB and AkT signaling pathways to supress virus-induced cellular apoptosis and facilitate viral growth (20392855), theaflavin-3,3'-digallate blocked NFkB activation (16880762) thereby hindered viral growth	Antiviral, against viral survival and growth		
All four ingredients			Rotavirus's entry into cells is partly facilitated by integrin, and rotavirus replication upregulated alpha2beta1 and beta2 integrins via activation of PI3K pathways, leading to further enhanced viral entry (17942548), theaflavins educed PI3K and pAkT (14743383), thereby reduced the	Antiviral, against viral entry		

			enhancement of viral entry			
			Possible Antivriral Effect			
theaflavin-3,3' digallate (5.51ug/mL)			Rotavirus's entry into cells is partly facilitated by haemagglutinin (15165605), theaflavin-3,3' digallate inhibited haemagglutinin of influenza virus (8215301), if it also inhibits haemagglutinin of rotavirus, theaflavin-3,3' digallate may hinder the viral entry process leading to virak neutralisation	Antiviral, against viral entry		
**Combination 3**						
**wedelolactone (0.8uM)**	**Principal therapeutic component**	**63.5**	Potent androgen receptor (AR) antagonist (IC50 0.2uM) (17942463)	Anticancer, growth inhibitin, via AR	indole-3-carboxylaldehyde's assumed AR downregulation (17942463) complements wedelolactone's AR antagonism, leading to synergism	Complementary action
					luteolin reduced AR expression in dose and time dependent manner (18008333), which complements wedelolactone's AR antagonism, leading	Complementary action

					to synergism	
					luteolin inhibited c-Src activities (20215519), which hinders c-Src mediatedenhancement of AR activity and AR transactivation function (21135112, 18223692), thereby complementing wedelolactone's AR antagonism, leading to synergism	Complementary action
					luteolin downregulated FGF1R signaling (22269172) to hinder its crosstalk with AR and stimulation of AR activity (21465482), which complements wedelolactone's AR antagonism, leading to synergism	Complementary action
					luteolin inhibited topoisomerase II (19149659) to hinder AR and topoisomerase II beta binding mediated oncogenic rearrangement and DNA repair (20601956. 21385925), thereby complementing wedelolactone's AR antagonism, leading to synergism	Complementary action

					apigenin inhibited CK2 (21871133), CK2 inhibition lead to AR downregulation in prostate cancer cells (17044081), thereby complementing wedelolactone's AR antagonism, leading to synergism	Complementary action
					apigenin reduced EGFR and HER2 expression (21196218) to hinder EGFR and HER2 mediated AR activation and prostate cancer progression (19318561), thereby complementing wedelolactone's AR antagonism, leading to synergism	Complementary action
					apigenin inhibited NF-κB activation (21142820) to hinder its promotion of AR expression in prostate cancer cells (18701501, 19628766), thereby complmenting wedelolactone's AR antagonism, leading to synergism	Complementary action
					apigenin activated P53 (22227579), which helps downregulating AR in prostate cancer (18084622)	Complementary action
					luteolin and apigenin each suppressed Akt activation (20655656, 22084167), which hinders AkT-mediated AR upregulation in prostate cancer (21317204), thereby complementing wedelolactone's AR antagonism, leading	Complementary action

					to synergism	
					luteolin and apigenin each reduced CDK6 activity (20655656, 16648554), which hinders CDK6-mediated enhancement of AR transcriptional activity in prostate cancer cells (15790678) to complement AR antagonism, leading to synergism	Complementary action
					luteolin and apigenin each inhibited GSK-3β (IC50 1.5 and 1.9uM) (21443429), GSK-3β inhibition helps AR export from cell nucleus thereby diminishing its effects (21980429), which complements AR antagonism, leading to synergism	Complementary action
					luteolin and apigenin each inhibited HDAC (IC50 50-100 and 20-40 uM) (21074525, 22006862) to hinder HDAC's facilitating action on AR function in hormone-sensitive and castrate-resistant prostate cancer (19176386), thereby complementing wedelolactone's AR antagonism, leading to synergism	Complementary action

			inhibited the activity of DNA topoisomerase Iiα independent of AR (21315506)	Anticancer, apoptosis	.	
			inhibited IKK leading to reduced activation of AkT and NFkB (21704149)	Anticancer, growth inhibition and apoptosis	.	
			inhibited trypsin (12722155), trypsin may have tumor suppressive activity (14583448) and this activity may be hindered by wedelolactone	Counteractive action against anticancer effect	apigenin activated P53 (22227579), which helps downregulating AR in prostate cancer (18084622)	Complementary action
indole-3-carboxylaldehyde (656uM)	Cooperative	15238	indole-3-carboxylaldehyde's structural analog indole-3-carbinol (I3C) forms DIM under acidic conditions; with a more stable and more potent anticancer activity, I3C and DIM antagonized androgen binding to AR and down-regulated AR in PCa cells (17942463). It has been speculated that indole-3-carboxylaldehyde may have similar activities (17942463)	Anticancer, growth inhibition, likely via AR down-regulation and antagonism	.	
luteolin (1.72uM)	Cooperative	3.13	AR antagonist (IC50 2.4uM) (17942463)	Anticancer, growth inhibition, via AR antagonism	indole-3-carboxylaldehyde's assumed AR downregulation (17942463) complements luteolin's AR antagonism, leading to synergism	Complementary action

			reduced AR expression in dose and time dependent manner (18008333), which complements its AR antagonist activity	Anticancer, growth inhibition, via AR reduction	.	
			suppressed Akt phosphorylation and activation (20655656), AR levels are upregulated by Akt in prostate cancer (21317204) and AR induces prostate cancer cell proliferation through mTOR activation (16885382), luteolin thus hinders AkT-mediated proliferation and survival, and helps containing AR levels	Anticancer, growth inhibition and apoptosis, via AR regulator and additional proliferation and survial signaling	.	
			reduced CDK4/6 activity (20655656), CDK6 associates with AR and enhances its transcriptional activity in prostate cancer cells (15790678), luteolin may thus hinders CDK4/6 mediated cell-cycle progression and AR expression	Anticancer, growth inhibition and cell cycle regulation, via dual AR and cell cycle regulator	.	
			inhibited c-Src activities (20215519), which hinders c-Src mediated growth signaling (18045060) and enhancement of AR activity and AR transactivation function (21135112, 18223692)	Anticancer, growth inhibition, via AR regulator and additional proliferation signaling	.	

			GSK-3β inhibitor (IC50 1.5uM) (21443429), GSK-3β inhibition helps AR export from cell nucleus thereby diminishing its effects (21980429), which complements AR antagonism	Anticancer, growth inhibition, via AR regulator	.	
			HDAC inhibitor (IC50 50-100uM) (21074525), which hinders HDAC's transcription regulation (19383284) and facilitating action on AR function in hormone-sensitive and castrate-resistant prostate cancer (19176386), thereby complementing AR antagonism	Anticancer, growth inhibition, via AR regulator and additional growth regulation	.	
			downregulated FGF1R signaling (22269172), which hinders its growth and survival signaling (19183230) and its crosstalk with AR and stimulation of AR activity (21465482)	Anticancer, growth inhibition, via AR regulator and additional growth and survival regulation	.	
			inhibited topoisomerases I and II (19149659), which hinders AR and topoisomerase II beta binding mediated oncogenic rearrangement and DNA repair (20601956. 21385925)	Anticancer, growth and survival inhibition, via AR partner and additional growth and survival regulation	.	

			activated AMPK (21468539), AMPK activation reduced growth of prostate cancer cells (19347029)	Anticancer, growth inhibition	.	
			inhibited proteasome (22292765), which may induce apoptosis (18347166)	Anticancer, apoptosis	.	
			downregulated Cyclin D1 (20655656), AR is strongly suppressed by cyclin D1 (21212260), luteolin may thus both hinder cell cycle and reduce AR suppression	Anticancer cell cycle regulation	.	
				Counteractive action against anticancer effect	.	
			activated P38 (22073986, 21762691), P38 activation may facilitate androgen-independent AR activation (19151763), which counters luteolin's AR antagonistic effect	Counteractive action against anticancer effect	.	
apigenin (3.02uM)	Cooperative	250	AR antagonist (IC50 9.8uM) (17942463)	Anticancer, growth inhibition, via AR antagonism	indole-3-carboxylaldehyde's assumed AR downregulation (17942463) complements apigenin's AR antagonism, leading to synergism	Complementary action

					luteolin reduced AR expression in dose and time dependent manner (18008333), which complements apigenin's AR antagonism, leading to synergism	Complementary action
			inhibited CK2 (21871133), CK2 inhibition lead to AR downregulation in prostate cancer cells (17044081), thereby complementing its AR antagonism activity	Anticancer, growth inhibition, via AR reduction		
			reduced EGFR and HER2 expression (21196218) to hinder EGFR and HER2 mediated AR activation and prostate cancer progression (19318561) and HER2 signaling in prostate cancer (15769631)	Anticancer, growth inhibition, via AR regulator and additional growth signaling inhibition		
			inactivated Akt (22084167), AR levels are upregulated by Akt in prostate cancer (21317204) and AR induces prostate cancer cell proliferation through mTOR activation (16885382), luteolin thus hinders AkT-mediated proliferation and survival, and helps containing AR levels	Anticancer, growth inhibition and apoptosis, via AR regulator and additional proliferation and survial signaling		

			reduced CDK2/4/6 activity (16648554), CDK6 associates with AR and enhances its transcriptional activity in prostate cancer cells (15790678), apigenin may thus hinders CDK4/6 mediated cell-cycle progression and AR expression	Anticancer, growth inhibition and cell cycle regulation, via dual AR and cell cycle regulator		
			GSK-3β inhibitor (IC50 1.9uM) (21443429), GSK-3β inhibition helps AR export from cell nucleus thereby diminishing its effects (21980429), which complements AR antagonism	Anticancer, growth inhibition, via AR regulator		
			HDAC inhibitor (IC50 20-40uM) (22006862), which hinders HDAC's transcription regulation (19383284) and facilitating action on AR function in hormone-sensitive and castrate-resistant prostate cancer (19176386), thereby complementing AR antagonism	Anticancer, growth inhibition, via AR regulator and additional growth regulation		
			inhibited NF-κB activation (21142820) to hinder its promotion of AR expression in prostate cancer cells (18701501, 19628766), thereby complmenting its AR antagonistic effect	Anticancer, growth inhibition, via AR regulator		

			activated P53 (22227579), which enhances P53 mediated tumor supressive activity in prostate cancer (21227058[), and helps downregulating AR in prostate cancer (18084622) and compensating for the reduced P53 activation due to AR downregulation	Anticancer, growth inhibition, via AR regulator and additional tumor suppression activity		
			downregulated Cox-2 expression (20691240) to hinder Cox-2's promotion of prostate cancer progression (12386924)	Anticancer, growth inhibition		
			increased Bax/Bcl-2 ratio, caspase 3 and cytochrome C, decreased Bcl-2, leading to anticancer apoptosis effects (20937639)	Anticancer, apoptosis		
			activated AMPK (21538580), AMPK activation reduced growth of prostate cancer cells (19347029)	Anticancer, growth inhibition		
			upregulated leptin receptor to induce apoptosis (21550230)	Anticancer, apoptosis		
			inhibited proteasome (22292765), which may induce apoptosis (18347166)			
			induced Hsp27 phosphorelation (21364669), Hsp27 mediated repression of AR function in prostate	Anticancer, growth inhibition, via AR regulator		

			cancer cells (19767773), but promoted IGF1R survival signaling in prostate cancer (20197463), which both complements and counters AR antagonistic activity			
				Counteractive action against anticancer effect		
			enhanced P38 phosphorelation (21615506), P38 activation may facilitate androgen-independent AR activation (19151763), which counters apigenin's AR antagonistic effect	Counteractive action against anticancer effect		
