# Supplementary material for: What Does It Take to Synergistically Combine Sub-Potent Natural Products into Drug-Level Potent Combinations?
Source: PLoS One. 2012 Nov 28;7(11):e49969. doi: 10.1371/journal.pone.0049969 (PMC3509152; doi:10.1371/journal.pone.0049969)
Supplement: Table S8 — Targets and potency-enhancing molecular interaction modes in 2 fully sub-potent natural product combinations with potencies of the principal component increased by >100 fold. (PDF) [file pone.0049969.s008.pdf]

Supplementary Table S8 Targets and potency-enhancing molecular interaction modes in 2 fully sub-potent natural product combinations with potencies of the principal component increased by >100 fold

Ingredient	Role in Combination	Dose Reduction Index	Target, Therapeutic Effect or Response (reference in Pubmed ID)	Effect type	Potency-Enhancing Synergistic Modes (reference in Pubmed ID)	Synergism Type
**Combination 1**						
**aescin (316ug/mL)**	**Principal therapeutic component**	158	disrupted membrane after metabolism by glycosidases (1171670), leading to haemolysis (21968386)	haemolysis	thymol affected cell membrane structure and enhanced permeability by generating asymmetries and membrane tensions (21660740), thereby facilitating the membrane insertion or crossing of aescin and its subsequent meabolism by glycosidases located in the internal side of membrane (15340929)	Intracellular bioavailability enhancement
thymol	sensitizer		affected cell membrane structure and enhanced permeability by generating asymmetries and membrane tensions (21660740)	permeability enhancement	.	
**Combination 2**						
***n*-butylidenephthalide (44.59ug/mL)**	**Principal therapeutic**	343	induced orphan nuclear receptor Nur77 to promote apoptosis (18577687, 21365711)	anticancer, apoptosis	.	

	**component**					
			suppresed human telomerase reverse transcriptase to restrict tumor growth (21553143)	anticancer, growth control	.	
			inhibited angiogenesis partly by activating p38 and ERK (21327473)	anticancer, anti-angiogenesis	.	
			induced Nur77 rexpression (18577687, 21365711) may lead to enhanced NFkB activity to reduce apoptosis (16082387), and to induce human telomerase reverse transcriptase for promoting tumor growth (15226182)	counteractive action against anticancer effect	z-ligustilide inhibited NFkB (20581853) to counter this counteractive action	anti-counteractive action
			promoted PI3K-Nurf2 crosstalk to enhance tumor survival signaling	counteractive action against anticancer effect	.	
			reduced P53 to reduce apoptosis (21398513)	counteractive action against anticancer effect	.	
senkyunolide A (10.4ug/mL)	Cooperative	347	anticancer mechanism unreported			
z-ligustilide (11.52ug/mL)	Cooperative	1.92	anticancer mechanism unreported			
