# Supplementary material for: What Does It Take to Synergistically Combine Sub-Potent Natural Products into Drug-Level Potent Combinations?
Source: PLoS One. 2012 Nov 28;7(11):e49969. doi: 10.1371/journal.pone.0049969 (PMC3509152; doi:10.1371/journal.pone.0049969)
Supplement: Table S9 — Targets and potency-enhancing molecular interaction modes in 9 fully sub-potent natural product combinations with potencies of the principal component increased by 10–100 fold. (PDF) [file pone.0049969.s009.pdf]

Supplementary Table S9 Targets and potency-enhancing molecular interaction modes in 9 fully sub-potent natural product combinations with potencies of the principal component increased by 10-100 fold

Ingredient	Role in Combination	Dose Reduction Index	Target, Therapeutic Effect or Response (reference in Pubmed ID)	Effect type	Potency-Enhancing Synergistic Modes (reference in Pubmed ID)	Synergism Type
**Combination 1**						
**Quillaja saponins (157ug/mL)**	**Principal therapeutic component**	14.3	induced haemolysis by inserting into and forming pores in membrane, and alterring Ca-K and Ca-Mn ATPase activities, and these activities are enhanced by its metabolism by glycosidases (19915999)	haemolysis	affected cell membrane structure and enhanced permeability of anticancer agents by generating asymmetries and membrane tensions (21660740), thereby facilitating membrane insertion or crossing of Quillaja saponins and their subsequent meabolism by glycosidases located in the internal side of membrane (15340929)	Intracellular bioavailability enhancement
thymol	sensitizer		affected cell membrane structure and enhanced permeability by generating asymmetries and membrane tensions (21660740)	permeability enhancement	.	
**Combination 2**						

**Salicylaldehyde (141ug/mL)**	**Principal therapeutic component**	>26	inhibited fungal antioxidant system proteins cytosolic superoxide dismutase, mitochondrial SOD and glutathione reductase, thereby producing antifungal effect (20803256)	antifungal	Linalool increased ROS species in certain cells by (19428344), which complements Salicylaldehyde's inhibition of fungal antioxidant system	complementary action
Linalool (281ug/mL)	Cooperative	>78	inhibited mitochondrial complexes and increased ROS species in certain cells by (19428344), both inhibition of mitochondrial function and ROS production may contribute to antifungal activity (16834605, 20803256)	antifungal		
**Combination 3**						
**berberine (256ug/mL)**	**Principal therapeutic component**	16	inhibited microbial division protein FtsZ to produce antimicrobial effect (18275156, 21060782)	antimicrobial		
			effluxed by a multidrug pump (10677479)	Efflux-mediated multidrug resistance	5'-methoxyhydnocarpin inhibited the mutidrug pump, thereby potentiated berberine's	Intracellular bioavailability enhancement

					antimicrobial activity (10677479)	
5'-methoxyhydnocarpin (10ug/mL)	sensitizer			potentiation		
**Combination 4**						
Linoleic acid (1mg/mL)	co-therapeuti c ingredient	20	inhibited bacterial enoyl-acyl carrier protein reductase FabI involved in fatty acid synthesis, thereby producing antibacterial effect (16146629, 21862391)	Antibacterial		
			effluxed by a farAB-encoded bacterial efflux pump (10447892)	Efflux-mediated multidrug resistance	Combination of Linoleic acid and Oleic acid inhibited bacterial efflux (21194895)	Intracellular bioavailability enhancement
			resisted by bacterial cell wall-anchored proteins SasF and SssF	Counteractive action		
Oleic acid (1mg/mL)	co-therapeuti c ingredient	20	inhibited bacterial enoyl-acyl carrier protein reductase FabI involved in fatty acid synthesis, thereby producing antibacterial effect	Antibacterial		

			(16146629, 21862391)			
			effluxed by a farAB-encoded bacterial efflux pump (10447892)	Efflux-mediated multidrug resistance	Combination of Linoleic acid and Oleic acid inhibited bacterial efflux (21194895)	Intracellular bioavailability enhancement
**Combination 5**						
**eriodictyol (0.8mg/mL)**	**Principal therapeutic component**	16.7	produced prooxidative DNA damage effect (19941260), which may contribute to antimicrobial activity	Antimicrobial	hesperetin has higher bioavailability (20447374) and is converted into eriodictyol in microbial culture by microbial enzymes (21873058), thereby enhancing the bioavailability of eriodictyol	Intracellular bioavailability enhancement
hesperetin (1mg/mL)	Cooperative	3.33	reduced reducing the activity of bacterial enzymes (18812032), which may contribute to antimicrobial activity	Antimicrobial		
			interacted with membrane better, leading to higher bioavailability inside cells (20447374)	Bioavailability		
**Combination 6**						

**eriodictyol (0.8mg/mL)**	**Principal therapeutic component**	16.7	produced prooxidative DNA damage effect (19941260), which may contribute to antimicrobial activity	Antimicrobial	Naringenin has higher bioavailability (2753859, 7603409, 8132524) and is converted into eriodictyol in microbial culture by microbial enzymes (21299115), thereby enhancing the bioavailability of eriodictyol	Intracellular bioavailability enhancement
Naringenin (1mg/mL)	Cooperative	2.5	inhibited VacA vacuolation (15770537) to hinder the release of nutrients necessary for microbial growth and survival (12814772), leading to antimicrobial activity	Antimicrobial		
			interacted with membrane better (2753859), leading to higher bioavailability inside cells (7603409, 8132524)	Bioavailability		
**Combination 7**						
**Berberine (500ug/mL)**	**Principal therapeutic component**	16	inhibited microbial division protein FtsZ to produce antimicrobial effect (18275156, 21060782)	antimicrobial		

			effluxed by a multidrug pump (10677479)	Efflux-mediated multidrug resistance	biochanin A inhibited the mutidrug pump, thereby potentiated berberine's antimicrobial activity (12952418)	Intracellular bioavailability enhancement
biochanin A (>312.5ug/mL)	Cooperative	>31.3	inhibited microbial growth (20335979, 21328137)	antimicrobial		
**Combination 8**						
**Berberine (500ug/mL)**	**Principal therapeutic component**	16	inhibited microbial division protein FtsZ to produce antimicrobial effect (18275156, 21060782)	antimicrobial		
			effluxed by a multidrug pump (10677479)	Efflux-mediated multidrug resistance	Genistein inhibited the mutidrug pump, thereby potentiated berberine's antimicrobial activity (12952418)	Intracellular bioavailability enhancement
Genistein (100ug/mL)	Cooperative	10	inhibited global synthesis of DNA, RNA and proteins, leading to antimicrobial activity (16328542)	Antimicrobial		
			stabilized covalent topoisomerase II-DNA cleavage complex, which may contribute to its	Antimicrobial		

			antimicrobial effect (14738897)			
**Combination 9**						
**Berberine (500ug/mL)**	**Principal therapeutic component**	16	inhibited microbial division protein FtsZ to produce antimicrobial effect (18275156, 21060782)	antimicrobial		
			effluxed by a multidrug pump (10677479)	Efflux-mediated multidrug resistance	Orobol inhibited the mutidrug pump, thereby potentiated berberine's antimicrobial activity (12952418)	Intracellular bioavailability enhancement
Orobol	sensitizer		Orobol inhibited mutidrug pump (12952418)	potentiation		
