# Supplementary material for: What Does It Take to Synergistically Combine Sub-Potent Natural Products into Drug-Level Potent Combinations?
Source: PLoS One. 2012 Nov 28;7(11):e49969. doi: 10.1371/journal.pone.0049969 (PMC3509152; doi:10.1371/journal.pone.0049969)
Supplement: Table S10 — Targets and potency-enhancing molecular interaction modes in 5 fully sub-potent natural product combinations with potencies of a non-principal component increased by 10–100 fold. (PDF) [file pone.0049969.s010.pdf]

Supplementary Table S10 Targets and potency-enhancing molecular interaction modes in 5 fully sub-potent natural product combinations with potencies of a non-principal component increased by 10-100 fold

Ingredient	Role in Combination	Dose Reduction Index	Target, Therapeutic Effect or Response (reference in Pubmed ID)	Effect type	Potency-Enhancing Synergistic Modes (reference in Pubmed ID)	Synergism Type
**Combination 1**						
**Vanillin (0.6mg/mL)**	**Principal therapeutic ingredient**	8	inhibited CYP53A15 to produce antifungal effect (18505250)	Antifungal	(+/-)-pinoresinol caused damage to fungal plasma membrane(20657496) to enhance vanillin's transport across fungal membrane (15868144)	Intracellular bioavailability enhancement
			polymerized by laccase lacA to reduce its antifungal effect	Counteractive action		
			catabolized by vanillin dehydrogenase vdh (22057861)	Counteractive action		
4-hydroxy-3-methoxycinnamaldehyde (0.4mg/mL)	Cooperative	2	antifungal mechanism unreported			
(+/-)-pinoresinol (1mg/mL)	Cooperative	10	caused damage to	Antifungal		

			fungal plasma membrane to produce antifungal effect (20657496)			
**Combination 2**						
**Vanillin (0.6mg/mL)**	**Principal therapeutic ingredient**	3	inhibited CYP53A15 to produce antifungal effect (18505250)	Antifungal	Scopoletin inhibited fungal efflux pumps (15826040)	Intracellular bioavailability enhancement
			polymerized by laccase lacA to reduce its antifungal effect	Counteractive action		
			catabolized by vanillin dehydrogenase vdh (22057861)	Counteractive action	Scopoletin inhibited fungal oxidation of vanillin to enhance its bioavailability (15826040)	Intracellular bioavailability enhancement
4-Hydroxy-3-methoxycinnamaldehyde (0.4mg/mL)	Cooperative	4	antifungal mechanism unreported			
Scopoletin (1.5mg/mL)	Cooperative	18.8	hindered fungi survival or germination, inhibited detoxification enzymes (15826040)	Antifungal		
**Combination 3**						

**berberine (125ug/mL)**	**Principal therapeutic ingredient**	4.2	inhibited microbial division protein FtsZ to produce antimicrobial effect (18275156, 21060782)	antimicrobial		
			effluxed by a multidrug pump (10677479)	Efflux-mediated multidrug resistance	chrysosplenol-D inhibited the mutidrug pump, thereby potentiated berberine's antimicrobial activity (12494348)	Intracellular bioavailability enhancement
chrysosplenol-D (250ug/mL)	Cooperative	10	antimicrobial mechanism unreported			
**Combination 4**						
**berberine (125ug/mL)**	**Principal therapeutic ingredient**	4.2	inhibited microbial division protein FtsZ to produce antimicrobial effect (18275156, 21060782)	antimicrobial		
			effluxed by a multidrug pump (10677479)	Efflux-mediated multidrug resistance	chrysoplenetin inhibited the mutidrug pump, thereby potentiated berberine's antimicrobial activity (12494348)	Intracellular bioavailability enhancement

chrysoplenetin (250ug/mL)	Cooperative	40	antimicrobial mechanism unreported			
**Combination 5**						
**curcumin (3.1uM)**	**Principal therapeutic ingredient**	3.1	downregulated Notch1 and Bcl-xL to inactivate NFkB, thereby promoting growth inhibition and apoptosis (16628653)	Anticancer, growth inhibition, apoptosis	isoflavone inhibited Notch, NFkB and AkT, and activated P53(22200028) to complement curcumin's action on Notch1 and Bcl-xL (16628653), thereby further promoting apoptosis	Complementary action
			activated P38, thereby downregulating Bcl2, survivin and AkT signaling to promote apoptosis (19676105)	Anticancer, apoptosis	isoflavone inhibited Notch, NFkB and AkT, and activated P53(22200028) to complement curcumin's action on Bcl2, survivin and AkT (19676105), thereby further promoting apoptosis	Complementary action
			inhibited AKT-mTOR pathway to promote anticancer effect (21450334)	Anticancer, growth inhibition		
isoflavone (183uM)	Cooperative	18.3	inhibited Notch, NFkB and AkT, and activated P53 to promote	Anticancer, apoptosis		

			apoptosis (22200028)			
